# The Huntington disease protein accelerates breast tumour development and metastasis through ErbB2/HER2 signalling

**DOI:** 10.1002/emmm.201201546

**Published:** 2013-01-09

**Authors:** Cristovão Moreira Sousa, John Russel McGuire, Morgane Sonia Thion, David Gentien, Pierre de la Grange, Sophie Tezenas du Montcel, Anne Vincent-Salomon, Alexandra Durr, Sandrine Humbert

**Affiliations:** 1 Institut CurieParis, France; 2 CNRS UMR 3306Orsay, France; 3 INSERM U1005Orsay, France; 4 Department of Translational ResearchParis, France; 5 GenoSplice Technology, Institut Universitaire d'hématologieParis, France; 6 AP-HP, Charles-Foix Clinical Research Unit, Department of Biostatistics and Medical Informatics, Hôpital de la Salpêtrière, University Pierre et Marie CurieParis, France; 7 ER4, Modelling in Clinical Research, University Pierre et Marie CurieParis, France; 8 Department of PathologyParis, France; 9 INSERM U830Paris, France; 10 Département de Génétique et Cytogénétique, Centre de Recherche de l'Institut du Cerveau et de la Moelle épinière, University Pierre et Marie Curie Pierre, UMR-S975Paris, France; 11 INSERM U975, Département de Génétique et CytogénétiqueParis, France; 12 CNRS UMR 7225, Département de Génétique et CytogénétiqueParis, France; 13 AP-HP, Département de Génétique et Cytogénétique, Hôpital de la SalpêtrièreParis, France

**Keywords:** breast cancer, dynamin, huntingtin, migration, polyglutamine

## Abstract

In Huntington disease (HD), polyglutamine expansion in the huntingtin protein causes specific neuronal death. The consequences of the presence of mutant huntingtin in other tissues are less well understood. Here we propose that mutant huntingtin influences breast cancer progression. Indeed, we show that mammary tumours appear earlier in mouse breast cancer models expressing mutant huntingtin as compared to control mice expressing wild-type huntingtin. Tumours bearing mutant huntingtin have a modified gene expression pattern that reflects enhanced aggressiveness with the overexpression of genes favouring invasion and metastasis. In agreement, mutant huntingtin accelerates epithelial to mesenchymal transition and enhances cell motility and invasion. Also, lung metastasis is higher in HD conditions than in control mice. Finally, we report that in HD, the dynamin dependent endocytosis of the ErbB2/HER2 receptor tyrosine kinase is reduced. This leads to its accumulation and to subsequent increases in cell motility and proliferation. Our study may thus have important implications for both cancer and HD.

## INTRODUCTION

The bulk of interest in the huntingtin protein has centred on the fact that, when mutated, huntingtin causes Huntington's disease (HD), a devastating neurodegenerative disorder. The mutation is an abnormally expanded polyglutamine (polyQ) stretch in the N-terminus of the protein. Given the adult onset and dysfunction and death of adult neurons that characterizes HD, most studies have focused on the toxic effects elicited by polyQ-huntingtin in post-mitotic neurons. However, HD is also associated with peripheral manifestations including weight loss and muscle wasting (Sassone et al, 2009; van der Burg et al, 2009). These symptoms may not be linked only to secondary manifestations of neuronal dysfunctions but also to the presence of polyQ-huntingtin in other dysfunctioning tissues. Indeed, the protein is ubiquitous, with high levels produced by the brain and outside the nervous system (Trottier et al, 1995). At the subcellular level, huntingtin is found both in the cytoplasm and in the nucleus (Hoogeveen et al, 1993; Kegel et al, 2002; Trottier et al, 1995). It associates with various organelles and structures, such as clathrin-coated vesicles, endosomal and endoplasmic compartments, mitochondria and microtubules. Consistent with this subcellular localization, huntingtin interacts with proteins involved in gene expression, intracellular transport, intracellular signalling and metabolism and thus appears to be involved in various cellular functions (Harjes & Wanker, 2003; Li & Li, 2004). Indeed, it is established that huntingtin plays critical roles in transcription, endocytosis, microtubule-based transport of organelles and mitosis in both neuronal and non-neuronal cells (Gauthier et al, 2004; Godin et al, 2010; Zuccato et al, 2010).

The presence of huntingtin in several tissues and its involvement in fundamental biological processes strongly suggest that huntingtin could be essential outside the brain. This is further underlined by the indispensability of huntingtin as revealed by the early embryonic lethality at day 7.5 of the complete knock-out of the huntingtin gene in mouse (Duyao et al, 1995; Nasir et al, 1995; White et al, 1997; Zeitlin et al, 1995). A partial depletion of huntingtin specifically showed its requirement for the generation of the three lineages that derived from the epiblast and lead to the formation of mesoderm, endoderm and ectoderm during gastrulation (Woda et al, 2005). Finally, huntingtin is required for the generation and expansion of haematopoietic cells in mouse and zebrafish (Lumsden et al, 2007; Metzler et al, 2000).

PolyQ-huntingtin induces death of neurons in the brain via distinct but complementary pathways including deregulation of apoptosis and/or autophagy, altered transcription, metabolism and cellular stress responses (Borrell-Pages et al, 2006; Zuccato et al, 2010). These disturbances were originally mostly attributed to the gain of new toxic functions of polyQ-huntingtin. However, there is growing evidence that loss of the normal functions of wild-type huntingtin could act concomitantly and synergistically with the gain of new toxic functions. For example, huntingtin function in the microtubule (MT)-based transport of brain derived neurotrophic factor (BDNF) vesicles is lost in HD, leading to a decreased trophic support provided by the cortical neurons to the striatal neurons (Gauthier et al, 2004). Thus, mutant huntingtin affects biological processes shared by all cells in the organism and this may impact on the homeostasis of the tissues where it is expressed.

Similarly, given the broad expression and basic cellular functions of wild-type huntingtin, it seems likely that mutant huntingtin may affect the aetiology of other diseases. Here we investigated whether mutant huntingtin could influence the progression of breast cancer after we found the protein to be expressed in both normal mammary epithelia and tumours. We demonstrate that mutant huntingtin accelerates tumourigenesis in two mouse breast cancer models, increases epithelial–mesenchymal transition (EMT) of cancer cells and favours lung metastasis in mice. Further analyses support that this occurs, at least in part, through the hyperactivation of the ErbB2/HER2 pathway. We thus propose a link between molecular pathways underlying neurodegeneration, cancer tumourigenesis and metastasis through mutant huntingtin.

## RESULTS

### Mutant huntingtin is expressed in human breast tumours

While research on HD has mostly focused on neurological symptoms, we investigated whether breast cancer could be influenced by the expression of mutant huntingtin. We identified 12 HD patients with breast cancer (Supporting Information Fig S1A). An inverse correlation between the length of the abnormal CAG expansion and the age at symptom onset in HD has been documented (Zoghbi & Orr, 2000). We also found a relationship between earlier ages of breast cancer onset and longer CAG repeats (Supporting Information Fig S1B; Pearson correlation coefficient = −0.58, *p*-value = 0.04).

We had access to cancer biopsies from HD patients and used immunohistochemistry with an antibody recognizing both the wild-type and mutant forms of huntingtin (Supporting Information Fig S1C). Positive staining was observed in normal residual tissue and invasive cells. We also used a mutant specific antibody (Supporting Information Fig S1D) and found mutant huntingtin in normal residual tissue and invasive cells with a strong nuclear staining. Thus, mutant huntingtin is expressed in breast tumours where it may influence cancer progression.

### PolyQ-huntingtin promotes mammary carcinogenesis

**Figure 1:**
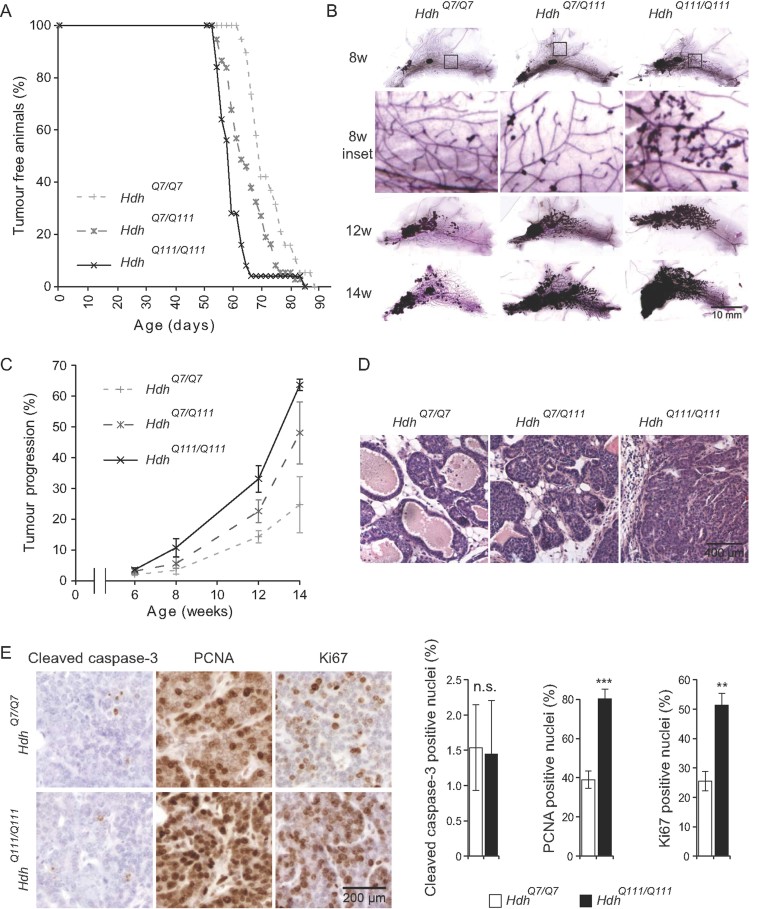
Oncogene-induced mammary tumours develop faster in HD mice Tumour-free survival curves of MMTV-PyVT/*Hdh*^*Q7/Q7*^ (*Hdh*^*Q7/Q7*^*; t*_50_ = 65 ± 1 days; *n* = 19), MMTV-PyVT/*Hdh*^*Q7/Q111*^ (*Hdh*^*Q7/Q111*^; *t*_50_ = 62 ± 4 days; *n* = 37) and MMTV-PyVT/*Hdh*^*Q111/Q111*^ (*Hdh*^*Q111/Q111*^; *t*_50_ = 52 ± 2 days; *n* = 25) mice. Kaplan–Meier Analysis, Logrank test: *p*-value < 0.0001.Whole mount carmine aluminium staining of MMTV-PyVT/*Hdh*^*Q7/Q7*^, MMTV-PyVT/*Hdh*^*Q7/Q111*^ and MMTV-PyVT/*Hdh*^*Q111/Q111*^ abdominal mammary glands at 8, 12 and 14 weeks.Percentage of tumoural tissue evaluated on whole mount carmine aluminium staining (at least *n* = 3 mice per genotype and per time point). At 14 weeks, MMTV-PyVT/*Hdh*^*Q7/Q7*^ *versus* MMTV-PyVT/*Hdh*^*Q7/Q111*^: *p*-value = 0.0186; MMTV-PyVT/*Hdh*^*Q7/Q7*^ *versus* MMTV-PyVT/*Hdh*^*Q111/Q111*^: *p*-value = 0.0024; MMTV-PyVT/*Hdh*^*Q7/Q111*^ *versus* MMTV-PyVT/Hdh^*Q111/Q111*^: *p*-value = 0.1558.Hematoxylin and eosin staining of sections from MMTV-PyVT/*Hdh*^*Q7/Q7*^, MMTV-PyVT/*Hdh*^*Q7/Q111*^ and MMTV-PyVT/*Hdh*^*Q111/Q111*^ tumours (4th mammary gland, 14 weeks).Immunohistochemical staining of sections from MMTV-PyVT/*Hdh*^*Q7/Q7*^ and MMTV-PyVT/*Hdh*^*Q111/Q111*^ tumours (4th mammary gland, 14 weeks) with antibodies against cleaved caspase-3, PCNA and Ki67. The graphs represent the quantitative assessments of the percentage of cleaved caspase-3 (*p*-value = 0.3583), PCNA (***p-value = 0.0010) and Ki67 (**p-value = 0.0080) positive cells (three tumours per genotype, at least 1000 cells scored per condition). n.s., not significant.

We then generated mice that express the activated polyomavirus middle T antigen (PyVT) oncogene and polyQ-huntingtin. Expression of PyVT under the control of the mouse mammary tumour virus (MMTV) promoter induces mammary adenocarcinoma formation (Guy et al, 1992). MMTV-PyVT mice were crossed with an HD mouse model, the *Hdh*^*Q111/Q111*^ mouse line which carries an abnormal 111 CAG repeat expansion in the huntingtin gene encoding an abnormally expanded polyQ stretch in huntingtin (Wheeler et al, 1999). HD behavioural and motor phenotypes arise much later than PyVT-induced cancer in these mice (Menalled et al, 2009). Compared with MMTV-PyVT mice expressing wild-type huntingtin (MMTV-PyVT/*Hdh*^*Q7/Q7*^), tumours appeared earlier in MMTV-PyVT/*Hdh*^*Q111/Q111*^ mice (Fig 1A). Heterozygous MMTV-PyVT/*Hdh*^*Q7/Q111*^ mice exhibited an intermediate phenotype. Examination of whole-mount mammary glands from virgin female MMTV-PyVT/*Hdh*^*Q111/Q111*^ mice at 8, 12 and 14 weeks (Fig 1B) revealed larger mammary adenocarcinomas than in the huntingtin heterozygous condition, themselves larger than those produced in the wild-type background at the same age. From these stainings, we evaluated the tumour progression by determining the percentage of the mammary gland composed of tumoural tissue at different time points (Fig 1C). Tumour progression was increased in MMTV-PyVT/*Hdh*^*Q111/Q111*^ mice as compared to MMTV-PyVT/*Hdh*^*Q7/Q111*^ and MMTV-PyVT/*Hdh*^*Q7/Q7*^ mice. These results were further confirmed by haematoxylin and eosin staining analysis (4th mammary gland at 14 weeks of age; Fig 1D). Mammary tumours in the wild-type mice were more differentiated than those in the polyQ situation. In the heterozygous situation, the situation was intermediate.

We tested whether the effect on tumour growth could result from changes in apoptosis and cellular proliferation by immunohistochemistry. Apoptosis detected by cleaved caspase-3 immunohistochemistry was similar in MMTV-PyVT/*Hdh*^*Q111/Q111*^ mammary tumour as compared to control (Fig 1E). In contrast, the protein levels of proliferation markers proliferating cell nuclear antigen protein (PCNA) and Ki67 were markedly higher in MMTV-PyVT/*Hdh*^*Q111/Q111*^ tumours.

Similar results were obtained when breeding HD mice with the MMTV-ErbB2 mouse breast cancer model (neu/HER2; Supporting Information Fig S2A and B; Muller et al, 1988). Thus, the presence of polyQ-huntingtin accelerates cell proliferation and mammary carcinogenesis in two breast cancer models.

### PolyQ-huntingtin induces gene expression changes in PyVT mammary tumours

**Figure 2:**
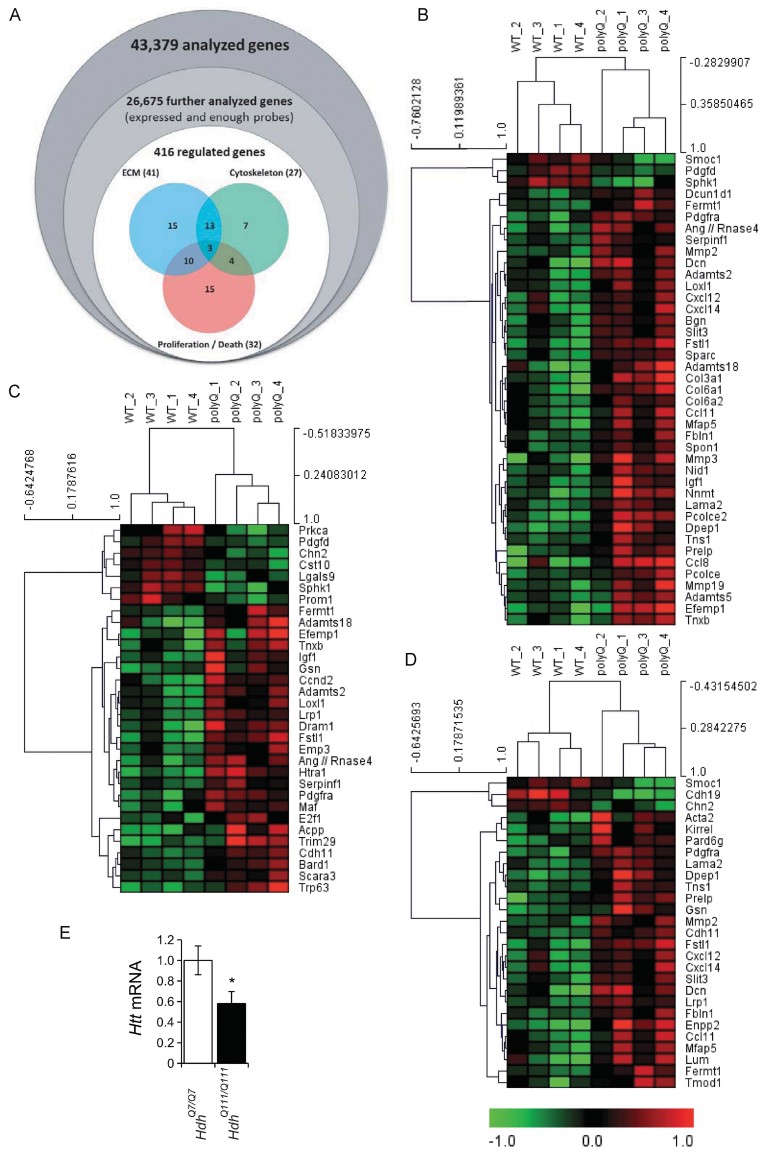
PolyQ-huntingtin causes differential gene expression in tumours **A.** Venn diagram for genes found to be regulated using Mouse Exon 1.0 ST Affymetrix array chips in 4 MMTV-PyVT/*Hdh*^*Q111/Q111*^ *versus* 4 MMTV-PyVT/*Hdh*^*Q7/Q7*^ tumours (6 weeks after detection), which met the criteria of a *p*-value < 0.05 and a fold-change of more than 1.5.**B–D.** Hierarchical clustering for ECM remodelling (**B**), cytoskeleton associated (**C**), and proliferation/cell death genes (**D**). WT: MMTV-PyVT/*Hdh*^*Q7/Q7*^ tumours; polyQ: MMTV-PyVT/*Hdh*^*Q111/Q111*^ tumours.**E.** mRNA levels of huntingtin as determined by quantitative PCR analysis in 4 MMTV-PyVT/*Hdh*^*Q7/Q7*^ and 3 MMTV-PyVT/*Hdh*^*Q111/Q111*^ tumours (*p-value = 0.0165).

To decipher the molecular events that lead to the increased tumourigenesis associated with polyQ-huntingtin expression, we examined the gene expression patterns of MMTV-PyVT/*Hdh*^*Q7/Q7*^ and MMTV-PyVT/*Hdh*^*Q111/Q111*^ mammary tumours using Affymetrix Mouse Exon 1.0 ST microarrays (Fig 2). Out of 43,379 genes analysed in four samples of each type of tumour, 416 genes were found to be differentially regulated with fold differences of at least 1.5 (*p*-value < 0.05), and 171 of these encoded a known protein (Fig 2A and Supporting Information Table S1). Most of the genes affected by polyQ-huntingtin expression (73%) were up-regulated in the tumours expressing polyQ-huntingtin compared to their expression levels in the wild-type tumours (Supporting Information Fig S3A and Table S1). Hierarchical clustering analysis of regulated genes confirmed that the tumour samples could be assigned to two primary clusters of tumours (Fig. 2 and Supporting Information Fig S3A). The left group of the dendogram contained the control tumour samples and the right group the HD tumour samples, with replicates in each cluster demonstrating substantial expression pattern homogeneity.

We confirmed the results generated by the gene microarrays for a subset of genes involved in relevant pathways (Supporting Information Fig S3B). The differences in the mRNA levels of cadherin 11, BRCA1-associated RING domain protein (BARD-1), matrix metalloproteinase 3 (MMP3), cyclin D2 were reflected by corresponding differences in the protein level as revealed by immunoblotting. Remarkably, mutant huntingtin mRNA (FC = −1.56; *p*-value = 4.7 × 10^−4^) were lower than those of wild-type huntingtin. Analysis of the levels of huntingtin transcripts by quantitative real-time RT-PCR in MMTV-PyVT/*Hdh*^*Q7/Q7*^ and MMTV-PyVT/*Hdh*^*Q111/Q111*^ breast tumours confirmed this observation (Fig 2E). Furthermore, the decreased levels of mutant huntingtin transcripts were accompanied by a decrease of mutant huntingtin protein levels as compared to the wild-type situation (Supporting Information Fig S4A), suggesting that concomitantly to the presence of the abnormal polyQ-huntingtin, reduced levels of the wild-type protein could also participate in the increased tumourigenesis in HD.

We then performed unsupervised analysis and pathway enrichment studies using all Gene Ontology (GO) terms (Supporting Information Table S2). The GO terms immune response (GO:0006955), adhesion (GO:0022610; GO:0007155), extracellular matrix organization (GO:0030198) and locomotion (GO:0040011) appeared dysregulated with *p*-values smaller than 10^−4^. All of these pathways are known to participate in tumour progression. In agreement with the earlier appearance of the tumours expressing mutant huntingtin as compared to the one expressing wild-type huntingtin, the biological processes of several GO categories related to cell death, apoptosis and proliferation were also affected (*p*-values smaller than 5 × 10^−2^). We confirmed these observations using hierarchical clustering and found changes in the levels of 67 mRNAs from genes associated with such functions as extracellular matrix remodelling, cytoskeleton organization and cellular death and proliferation (Fig 2B–D).

Thus the genetic signature of tumours expressing polyQ-huntingtin may correlate with their earlier appearance as compared to the one of tumours expressing wild-type huntingtin. Futhermore, this signature of overexpressed invasion and metastasis genes suggests enhanced aggressiveness of HD tumours.

### PolyQ-huntingtin increases EMT and pulmonary metastasis

Microarray data revealed that mesenchymal-associated genes and genes encoding extracellular matrix ECM remodelling proteins associated with EMT were up-regulated in the polyQ-huntingtin-expressing tumours as compared to control tumours, including the genes MMP2 (FC = 1.66; *p*-value = 3.92 × 10^−3^) and MMP3 (FC = 2.47; *p*-value = 1.58 × 10^−2^; Supporting Information Table S1). We also compared our microarray data to a multi-cancer stage-associated gene expression signature enriched in EMT markers (Cheng et al, 2012; Kim et al, 2010). Among the 64 genes corresponding to the top 100 probe sets of the signature, we found 31% of them to be significantly upregulated in HD conditions (Supporting Information Table S3). This suggested that EMT could be accelerated in HD during tumour progression.

**Figure 3:**
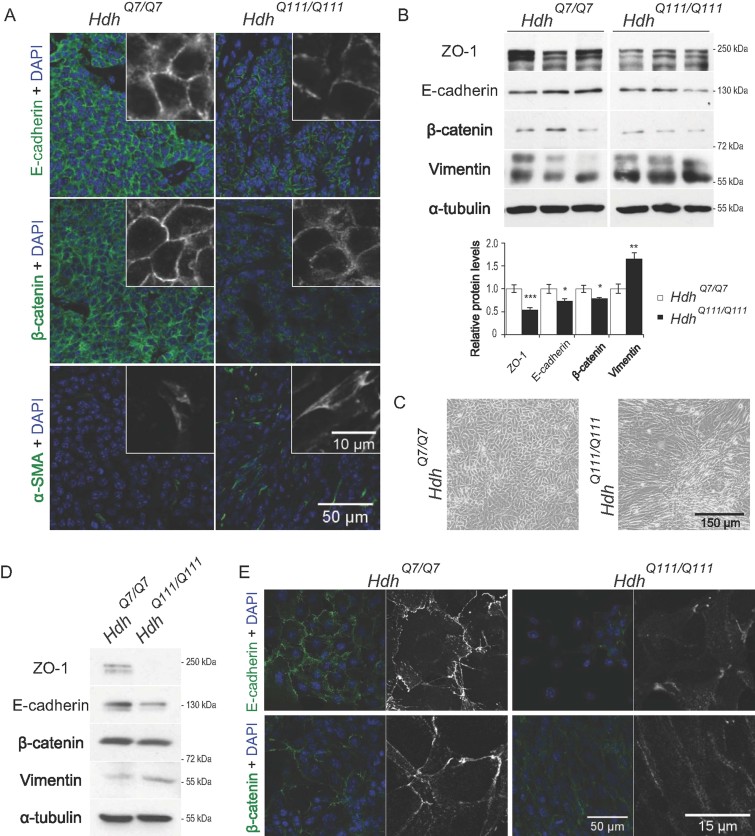
PolyQ-huntingtin accelerates EMT Immunostaining of sections from MMTV-PyVT/*Hdh*^*Q7/Q7*^ (*Hdh*^*Q7/Q7*^) and MMTV-PyVT/*Hdh*^*Q111/Q111*^ (*Hdh*^*Q111/Q111*^) tumours (originating from the 3rd mammary gland, 14 weeks) for endogenous E-cadherin, β-catenin and α-SMA.Immunoblotting of total extracts from MMTV-PyVT/*Hdh*^*Q7/Q7*^ and MMTV-PyVT/*Hdh*^*Q111/Q111*^ tumours (originating from the 3rd mammary gland, 14 weeks, *n* = 6 tumours per genotype, two independent immunoblotting) for the presence of ZO-1, E-cadherin, β-catenin, vimentin and α-tubulin. ZO-1: ***p-value = 0.0007; E-cadherin: *p-value = 0.0295; β-catenin: *p-value = 0.0238; vimentin: **p-value = 0.0039.Differential interfering contrast images of cells from MMTV-PyVT/*Hdh*^*Q7/Q7*^ and MMTV-PyVT/*Hdh*^*Q111/Q111*^ dissociated tumours.Immunoblotting of extracts from PyVT/*Hdh*^*Q7/Q7*^ and PyVT/*Hdh*^*Q111/Q111*^ cells as in (**B**).PyVT/*Hdh*^*Q7/Q7*^ and PyVT/*Hdh*^*Q111/Q111*^ cells are immunostained for endogenous E-cadherin and β-catenin.

To test this hypothesis, we analysed primary tumour sections by immunohistochemistry (Fig 3A). Lowered levels of the cell–cell adhesion proteins E-cadherin and β-catenin were observed in the MMTV-PyVT/*Hdh*^*Q111/Q111*^ tumours, while the mesenchymal marker α-smooth muscle actin (α-SMA) was increased. We then analysed extracts from MMTV-PyVT/*Hdh*^*Q7/Q7*^ and MMTV-PyVT/*Hdh*^*Q111/Q111*^ tumours by immunoblotting (Fig 3B). The MMTV-PyVT/*Hdh*^*Q111/Q111*^ tumours had lower levels of the tight junction protein zonula occludens 1 (ZO1), E-cadherin and β-catenin, and an increased level of the mesenchymal marker vimentin compared to MMTV-PyVT/*Hdh*^*Q7/Q7*^ tumours. Similarly, E-cadherin, β-catenin and vimentin levels were affected in MMTV-ErbB2/*Hdh*^*Q111/Q111*^ tumours as compared to MMTV-ErbB2/*Hdh*^*Q7/Q7*^ tumours (Supporting Information Fig S2C). We next derived primary tumour cells from the MMTV-PyVT/*Hdh*^*Q7/Q7*^ and MMTV-PyVT/*Hdh*^*Q111/Q111*^ tumours (PyVT/*Hdh*^*Q7/Q7*^ and PyVT/*Hdh*^*Q111/Q111*^, respectively). Doubling-time measurements showed no significant difference between wild-type (PyVT/*Hdh*^*Q7/Q7*^) and polyQ-huntingtin-expressing cells (PyVT/*Hdh*^*Q111/Q111*^) (12.38 ± 0.25 h and 12.65 ± 0.22 h, respectively, PLSD Fisher test *p*-value = 0.4357). However, further confirming the microarray data, polyQ-huntingtin expression led to a mesenchymal-like phenotype in the PyVT/*Hdh*^*Q111/Q111*^ cells, which became scattered and elongated compared to phenotype of the PyVT/*Hdh*^*Q7/Q7*^ cells (Fig 3C). Also, the observations done on tumours with respect to the levels of ZO1, E-cadherin, β-catenin and vimentin were confirmed in an immunoblot analysis of extracts from PyVT/*Hdh*^*Q7/Q7*^ and PyVT/*Hdh*^*Q111/Q111*^ tumour cells (Fig 3D). Immunostaining confirmed that E-cadherin and β-catenin production were substantially lower in polyQ-huntingtin-expressing cells (Fig 3E). Furthermore, β-catenin exhibited membrane localization in PyVT/*Hdh*^*Q7/Q7*^ cells and diffuse cytoplasmic localization in PyVT/*Hdh*^*Q111/Q111*^ cells, which is consistent with decreased cellular adhesion. In summary, mutant huntingtin expression in primary tumour tissue and in tumour-derived cells affects the levels of known cell adhesion markers and mesenchymal markers. Furthermore, when mutant huntingtin is expressed, tumour cells in culture adopt an altered morphology resembling a mesenchymal phenotype.

**Figure 4:**
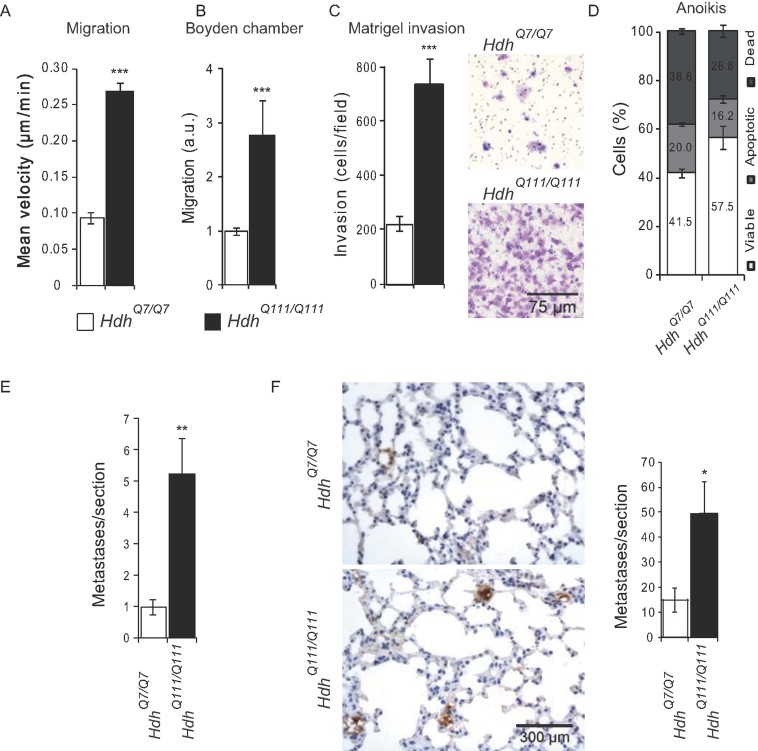
PolyQ-huntingtin promotes mammary cancer cell motility, resistance to cell death and distal metastases in the lung Random migration assays of PyVT/*Hdh*^*Q7/Q7*^ (*Hdh*^*Q7/Q7*^*)* or PyVT/*Hdh*^*Q111/Q111*^ (*Hdh*^*Q111/Q111*^) cells (3 independent primary cultures; at least 100 cells recorded). ***p-value < 0.0001.Boyden chambers assays for PyVT/*Hdh*^*Q7/Q7*^ and PyVT/*Hdh*^*Q111/Q111*^ cells (at least four independent primary cultures in duplicate per genotype). ***p-value < 0.0001.Boyden chambers with matrigel invasion assays for PyVT/*Hdh*^*Q7/Q7*^ and PyVT/*Hdh*^*Q111/Q111*^ cells (at least seven independent primary cultures in duplicate per genotype). Representative micrographs of invasion filter membranes after violet staining (*t* = 48 h) are shown. ***p-value < 0.0001.PyVT/*Hdh*^*Q7/Q7*^ and PyVT/*Hdh*^*Q111/Q111*^ cells were cultured in suspension, stained for annexin V and propidium iodide, and analysed by a flow cytometry analysis (four independent experiment, at least four independent primary cultures per genotype). Live, apoptotic and dead cell populations are quantified (live cells, *p*-value = 0.0099; apoptotic cells, *p*-value = 0.0835; dead cells, *p*-value = 0.0169).Lungs from 12 weeks MMTV-PyVT/*Hdh*^*Q7/Q7*^ and MMTV-PyVT/*Hdh*^*Q111/Q111*^ mice are immunostained with anti-PyVT antibodies. The mean number of metastatic lesions per section is shown (*n* = 5 lungs per genotype; **p-value = 0.0060).Lungs from mice grafted with MMTV-PyVT/*Hdh*^*Q7/Q7*^ and MMTV-PyVT/*Hdh*^*Q111/Q111*^ tumours immunostained with anti-PyVT antibodies. The mean number of metastatic lesions per section is shown (MMTV-PyVT/*Hdh*^*Q7/Q7*^: *n* = 5 lungs; MMTV-PyVT/*Hdh*^*Q111/Q111*^; *n* = 4 lungs; *p-value = 0.0266). A second experiment gave similar results.

We then tested whether mutant huntingtin played a role in cell motility, which is a functional marker of EMT. We performed random cell migration assays with PyVT/*Hdh*^*Q7/Q7*^ and PyVT*/Hdh*^*Q111/Q111*^ primary tumour cells (Fig 4A). Cells expressing polyQ-huntingtin moved faster than the corresponding control cells. We also assessed the directed cell migration capacity of the two cell types using Boyden chamber assays, in which PyVT/*Hdh*^*Q111/Q111*^ cells transmigrated faster in response to serum than the PyVT/*Hdh*^*Q7/Q7*^ cells (Fig 4B). To compare the invasiveness of PyVT/*Hdh*^*Q7/Q7*^ and PyVT/*Hdh*^*Q111/Q111*^ cells, we used Boyden chambers containing a layer of ECM proteins on top of the membrane (Fig 4C) and found that PyVT/*Hdh*^*Q111/Q111*^ cells were more invasive than PyVT/*Hdh*^*Q7/Q7*^ cells. Finally, we assessed cell viability in suspension cultures. PyVT/*Hdh*^*Q111/Q111*^ suspension cultures contained more live cells than PyVT/*Hdh*^*Q7/Q7*^ suspension cultures as measured by annexin V and propidium iodide (PI) staining and flow cytometric analysis (Fig 4D). In contrast, the percentage of apoptotic and dead cells were lower in the cells that expressed polyQ-huntingtin. Taken together these results show that polyQ-huntingtin expression in cancer cells is associated with enhanced migratory and invasive behaviours, and an elevated resistance to anoikis.

We then assessed whether EMT acceleration in polyQ-huntingtin expressing cells could influence metastasis by analysing lung metastasis in MMTV-PyVT/*Hdh*^*Q7/Q7*^ and MMTV-PyVT/*Hdh*^*Q111/Q111*^ mice (Fig 4E). In the HD context, metastasis was increased as compared to the wild-type situation. To exclude the possible effect of differential primary tumour growth on the lung metastasis analysis, we also grafted primary solid-tumour isolates from MMTV-PyVT/*Hdh*^*Q7/Q7*^ and MMTV-PyVT/*Hdh*^*Q111/Q111*^ mice into immunodeficient mice (Fig 4F). We found expression of polyQ-huntingtin in the engrafted tumour to increase the incidence of lung metastases as revealed by immunostaining of lung sections with an antibody directed against PyVT. Lung metastases were also more prevalent in immunodeficient mice engrafted with MMTV-ErbB2/*Hdh*^*Q111/Q111*^ tumours as compared to MMTV-ErbB2/*Hdh*^*Q7/Q7*^ tumours (Supporting Information Fig S2D). Thus, expression of polyQ-huntingtin in tumour cells leads to EMT and increased tumour metastasis.

### PolyQ-huntingtin leads to membrane accumulation of epidermal growth factor receptor ErbB2/HER2

**Figure 5:**
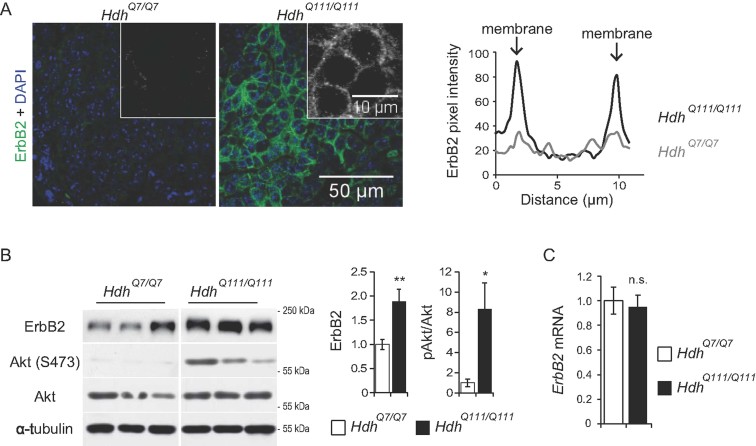
PolyQ-huntingtin promotes membrane ErbB2/HER2 accumulation and sustained signalling Immunostaining of sections from MMTV-PyVT/*Hdh*^*Q7/Q7*^ (*Hdh*^*Q7/Q7*^) and MMTV-PyVT/*Hdh*^*Q111/Q111*^ (*Hdh*^*Q111/Q111*^) tumours (originating from the 3rd mammary gland, 14 weeks) for the presence of ErbB2. Representative linescan analysis of the distribution of ErbB2 in wild-type and polyQ-huntingtin expressing cells within the tumour sections are shown.Immunoblotting of total extracts from MMTV-PyVT/*Hdh*^*Q7/Q7*^ and MMTV-PyVT/*Hdh*^*Q111/Q111*^ tumours (originating from the 3rd mammary gland, 14 weeks) for ErbB2, phosphorylated Akt at serine 473 (Akt(S473)), total Akt and α-tubulin (at least *n* = 5 tumours per genotype, two independent immunoblotting; ErbB2/tubulin: **p-value = 0.0057; Akt(S473)/Akt: *p-value = 0.0328).mRNA levels of ErbB2 in MMTV-PyVT/*Hdh*^*Q7/Q7*^ (*n* = 4) and MMTV-PyVT/*Hdh*^*Q111/Q111*^ (*n* = 3) tumours as determined by quantitative PCR analysis (*p*-value = 0.7441; n.s., not significant).

How does mutant huntingtin enhance tumour aggressiveness? One possible molecular mechanism underlying the progression from normal breast epithelia to invasive cancer cells involves the accumulation of ErbB receptors (Roepstorff et al, 2008). We evaluated the levels of ErbB2 (rat neu or human HER2) in MMTV-PyVT/*Hdh*^*Q7/Q7*^ and MMTV-PyVT/*Hdh*^*Q111/Q111*^ tumours by immunohistochemistry and found a marked increase of ErbB2 in polyQ-expressing tumours as compared to the control tumours (Fig 5A, left panels). The increase was specifically observed at the plasma membrane as revealed by linescan analysis (Fig 5A, right). Furthermore, it was confirmed by immunoblotting and corresponding quantification (Fig 5B). In heterozygous MMTV-PyVT/*Hdh*^*Q7/Q111*^ mice, the accumulation of ErbB2 was intermediate (Supporting Information Fig S4B). We then analysed the levels of total and activated Akt – a well described downstream target of ErbB2 – in control and polyQ-huntingtin tumours. We observed a statistically significant activation of this pathway as detected by the increase in the ratio of the active *versus* the total level of Akt in MMTV-PyVT/*Hdh*^*Q111/Q111*^ compared to MMTV-PyVT/*Hdh*^*Q7/Q7*^ tumours (Fig 5B). Thus polyQ-huntingtin leads to ErbB2 accumulation and Akt activation.

Our microarray data revealed no obvious difference in ErbB2 mRNA levels in wild-type *versus* polyQ-huntingtin mammary tumours (FC = −1.19; *p*-value = 8.57 × 10^−2^). Further analysis of the levels of ErbB2 transcripts by quantitative real-time RT-PCR in MMTV-PyVT/*Hdh*^*Q7/Q7*^ and MMTV-PyVT/*Hdh*^*Q111/Q111*^ breast tumours confirmed this observation (Fig 5C). Thus, it appears that ErbB2 accumulates at the membrane and the accumulation occurs at the posttranscriptional level.

### PolyQ-huntingtin interferes with HER2/ErbB2 dynamin dependent endocytosis

**Figure 6:**
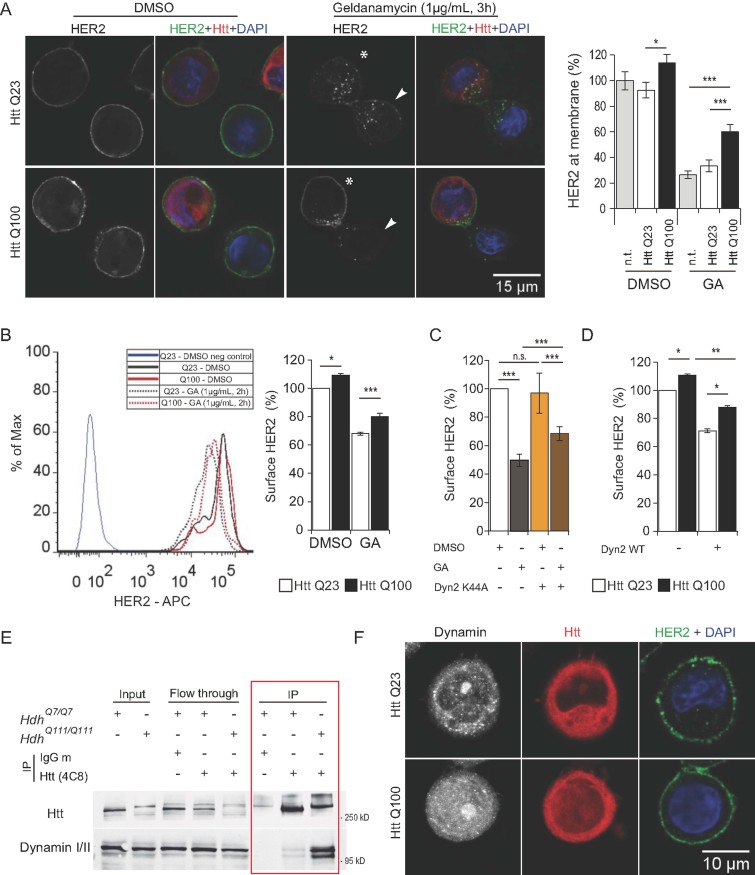
PolyQ-huntingtin inhibits ErbB2/HER2 endocytosis through a dynamin dependent mechanism **A,B.** Human SKBr3 cells are transfected with plasmids encoding full-length wild-type (pARIS-mCherry-httQ23, Htt Q23) and polyQ-huntingtin (pARIS-mCherry-httQ100, Htt Q100) and treated with Geldanamycin (GA) as indicated. n.t.: not transfected. (**A**) Cells are stained for HER2 and huntingtin (Htt). HER2 intensity at the membrane is quantified before and after treatment (at least *n* = 24 cells analysed per condition, three independent experiments). Htt Q23 DMSO *vs.* Htt Q100 DMSO: *p-value = 0.0204; n.t. GA *vs.* Htt Q100 GA: ***p-value < 0.0001; Htt Q23 GA *versus* Htt Q100 GA: ***p-value = 0.0006. (**B**) Cells are fixed, immunostained with the antibody recognizing the extracellular part of HER2 and analysed by flow cytometry. A representative flow cytometry profile is shown (left). The graphs in B, C and D represent mean HER2-APC fluorescence level (surface HER2; three independent experiments, 10,000 cells analysed per condition and experiment). Htt Q23 DMSO *versus* Htt Q100 DMSO: *p-value = 0.0141; Htt Q23 GA *versus* Htt Q100 GA: ***p-value = 0.0009.**C.** SKBr3 cells are transfected with a construct expressing a GTPase-defective mutant K44A dynamin (Dyn2 K44A), treated with Geldanamycin as indicated and analysed by flow cytometry. (Three independent experiments, 10,000 cells analysed per condition and experiment). DMSO *versus* GA, ***p-value < 0.0001; DMSO *versus* Dyn2 K44A DMSO, *p*-value > 0.9999; GA *versus* Dyn2 K44A GA, ***p-value < 0.0001; Dyn2 K44A DMSO *versus* Dyn2 K44A GA, ***p-value = 0.0002. n.s., not significant.**D.** SKBr3 cells are transfected with pARIS-mCherry-httQ23, pARIS-mCherry-httQ100 and a construct encoding wild-type dynamin (Dyn2 WT) and analysed by flow cytometry (three independent experiments, 10,000 cells analysed per condition and experiment). Htt Q23 *versus* Htt Q100, *p-value = 0.0491; Htt Q100 *versus* Htt Q100 + Dyn2 WT, **p-value = 0.0016; Htt Q23 + Dyn2 WT *versus* Htt Q100 + Dyn2 WT, *p-value = 0.0136.**E.** Huntingtin and dynamin interact in a polyQ-dependent manner. Huntingtin immunoprecipitation experiments were performed on cellular extracts from MMTV-PyVT/*Hdh*^*Q7/Q7*^ (*Hdh*^*Q7/Q7*^) and MMTV-PyVT/*Hdh*^*Q111/Q111*^ (*Hdh*^*Q111/Q111*^) tumours. Immunoprecipitation with mouse IgG (IgG m) is used as a control.**F.** SKBr3 cells are transfected with plasmids encoding full-length wild-type (pARIS-mCherry-httQ23) and polyQ-huntingtin (pARIS-mCherry-httQ100) and stained for huntingtin (Htt) and dynamin.

Huntingtin is involved in intracellular trafficking and endocytosis, and polyQ-huntingtin impairs these functions (Caviston et al, 2007; Gauthier et al, 2004; Velier et al, 1998). Given the increase in ErbB2 at the plasma membrane in the presence of polyQ-huntingtin, we wondered whether polyQ-huntingtin would influence ErbB2 internalization. We induced ErbB2 internalization by inhibiting the specific regulator of ErbB2 stability Hsp90, using Geldanamycin (Citri et al, 2004). To address this question, we used as a model system the human SKBr3 breast cancer cell line, which as it was derived from a HER2-positive tumour, expresses high levels of HER2 (Fig 6A). In these cells, HER2 is mainly localized at the plasma membrane. We transfected SKBr3 cells with full-length wild-type (pARIS-mCherry-httQ23, Htt Q23) and polyQ-huntingtin (pARIS-mCherry-httQ100, Htt Q100; Pardo et al, 2010). Upon Geldanamycin treatment of SKBr3 cells, the staining of HER2 at the plasma membrane was markedly lower in cells expressing exogenous huntingtin of normal CAG length (Fig 6A; upper panel, star) and in non-transfected cells (arrowhead). In contrast, the decrease of HER2 staining at the membrane triggered by Geldanamycin treatment was much less efficient in the presence of exogenously expressed polyQ-huntingtin (compare transfected cell – bottom panel, star – with non polyQ-huntingtin transfected cell – arrowhead). We then specifically addressed the effect of mutant huntingtin on internalization by examining levels of HER2 at the cell surface by flow cytometry analysis (Fig 6B). SKBr3 cells expressing wild-type and mutant huntingtin were treated with Geldanamycin and immunostained with an antibody recognizing the extracellular part of HER2 prior to flow cytometry. As expected (Figs 5 and 6A), in the absence of Geldanamycin treatment, HER2 accumulated at the SKBr3 cell surface when mutant huntingtin was expressed as compared with control cells. Upon Geldanamycin treatment, the internalization of HER2 in cells expressing Htt Q23 was greater than in cells expressing Htt Q100 (Fig 6B). These results indicate that polyQ-huntingtin interferes with HER2 internalization.

Geldanamycin was reported to induce endocytosis-mediated degradation of ErbB2 through a dynamin-dependent mechanism (Pedersen et al, 2008). Indeed, we confirmed that expressing a GTPase-defective mutant K44A dynamin 2 (Dyn2 K44A) in SKBr3 cells treated with Geldanamycin partially blocked Geldanamicin-induced down-regulation of HER2 from the plasma membrane (Fig 6C). We thus asked whether the effect of mutant huntingtin on HER2 endocytosis could be dynamin-dependent. We co-expressed wild-type dynamin with wild-type or mutant huntingtin in SKBr3 cells and analysed HER2 at the cell surface by flow cytometry (Fig 6D). Dynamin led to the decrease of HER2 at the cell surface and this effect was partially blocked in cells expressing mutant huntingtin, suggesting that the mutant huntingtin blockage of HER2 internalization is dynamin-dependent.

What is the underlying mechanism by which huntingtin interferes with dynamin? Wild-type huntingtin was shown to interact with dynamin by yeast two hybrid (Kaltenbach et al, 2007). We investigated whether huntingtin could interact with dynamin in primary tumours cells and the possible consequence of the abnormal polyQ expansion in mutant huntingtin. We carried out immunoprecipitation experiments with an anti-huntingtin antibody (Fig 6E). Dynamin interacted with endogenous huntingtin and the interaction was greatly enhanced when huntingtin contained an abnormally expanded polyQ stretch. Finally, we analysed the subcellular localization of these proteins by confocal microscopy. In SKBr3 cells expressing wild-type huntingtin, dynamin had a characteristic localization near the plasma membrane (Fig 6F). In the mutant situation, this localization where dynamin is known to act in endocytosis, was lost with dynamin being dispersed throughout the cytoplasm. We conclude that the stronger interaction of mutant huntingtin and dynamin leads to a redistribution of dynamin and a subsequent decreased endocytosis of HER2/ErbB2.

### Trastuzumab inhibits polyQ-huntingtin induced HER2 accumulation and downstream effects

**Figure 7:**
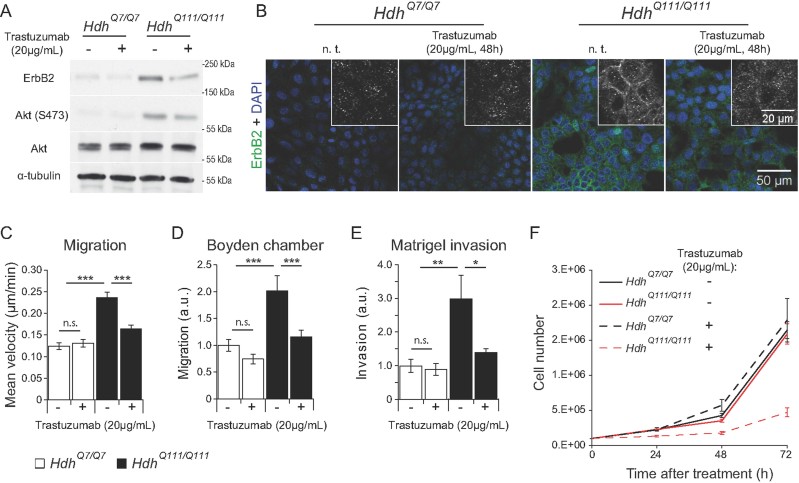
Trastuzumab inhibits polyQ-huntingtin induced HER2 accumulation and downstream effects on cell motility **A.** Immunoblotting of total extracts of PyVT/*Hdh*^*Q7/Q7*^ (*Hdh*^*Q7/Q7*^*)* and PyVT/*Hdh*^*Q111/Q111*^ (*Hdh*^*Q111/Q111*^) cells treated with Trastuzumab for ErbB2, phosphorylated Akt at serine 473 (Akt(S473)), total Akt and α-tubulin.**B.** PyVT/*Hdh*^*Q7/Q7*^ and PyVT/*Hdh*^*Q111/Q111*^ cells treated with Trastuzumab are immunostained for endogenous ErbB2. n.t.: not treated.**C–E.** Random migration (**C**), Boyden chambers (**D**) and Boyden matrigel invasion (**E**) assays for PyVT/*Hdh*^*Q7/Q7*^ or PyVT/*Hdh*^*Q111/Q111*^ cells treated with Trastuzumab. Random migration (three independent primary cultures, 118 cells recorded per condition; *Hdh*^*Q7/Q7*^ *vs. Hdh*^*Q7/Q7*^ Trastuzumab, *p*-value = 0.6326; *Hdh*^*Q7/Q7*^ *vs. Hdh*^*Q111/Q111*^, ***p-value < 0.0001; *Hdh*^*Q111/Q111*^ *vs. Hdh*^*Q111/Q111*^ Trastuzumab, ***p-value < 0.0001). Boyden chambers (three independent primary culture in duplicate per genotype; *Hdh*^*Q7/Q7*^ *vs. Hdh*^*Q7/Q7*^ Trastuzumab, *p*-value = 0.2682; *Hdh*^*Q7/Q7*^ *vs. Hdh*^*Q111/Q111*^, ***p-value = 0.0001; *Hdh*^*Q111/Q111*^ *vs. Hdh*^*Q111/Q111*^ Trastuzumab, ***p-value = 0.0007) and Boyden matrigel invasion (three independent primary cultures in duplicate per genotype; *Hdh*^*Q7/Q7*^ *vs. Hdh*^*Q7/Q7*^ Trastuzumab, *p*-value = 0.7599; Hdh^Q7/Q7^ *vs. Hdh*^*Q111/Q111*^, **p-value = 0.0012; *Hdh*^*Q111/Q111*^ *vs. Hdh*^*Q111/Q111*^ Trastuzumab, *p-value = 0.0101). n.s.: not significant.**F.** Cell number of PyVT/*Hdh*^*Q7/Q7*^ and PyVT/*Hdh*^*Q111/Q111*^ cells treated with Trastuzumab for 24, 48 and 72 h (at least *n* = 3 primary culture per genotype in three independent experiments; at 72 h: *Hdh*^*Q7/Q7*^ *vs. Hdh*^*Q111/Q111*^, *p*-value = 0.8391; *Hdh*^*Q7/Q7*^ Trastuzumab *vs. Hdh*^*Q111/Q111*^, *p*-value = 0.4605; *Hdh*^*Q7/Q7*^ *vs. Hdh*^*Q7/Q7*^ Trastuzumab, *p*-value = 0.5909; *Hdh*^*Q111/Q111*^ *vs. Hdh*^*Q111/Q111*^ Trastuzumab, *p*-value = 0.0002).

We then aimed to unequivocally address whether HER2 accumulation would explain the more aggressive phenotypes of HD tumours cells as compared to a control situation. For this purpose, we treated PyVT/*Hdh*^*Q7/Q7*^ and PyVT/*Hdh*^*Q111/Q111*^ cells with 20 µg/ml Trastuzumab, a monoclonal antibody targeting HER2/ErbB2. PolyQ-huntingtin led to ErbB2 accumulation in PyVT*/Hdh*^*Q111/Q111*^ primary tumour cells that was inhibited when cells were treated with Trastuzumab (Fig 7A; PyVT/*Hdh*^*Q111/Q111*^ normalized to 100% and PyVT/*Hdh*^*Q111/Q111*^/Trastuzumab: 41% ± 14%, four independent immunoblotting experiments of at least three samples, PLSD Fisher test *p* < 0.05). In particular, immunostainings specifically revealed a decrease of cell surface ErbB2 in PyVT*/Hdh*^*Q111/Q111*^ primary tumour cells upon Trastuzumab treatment (Fig 7B). Furthermore, Trastuzumab treatment was accompanied by an inhibition of ErbB2 triggered signalling in polyQ-huntingtin-expressing tumour cells. Indeed, the overactivation of Akt observed in PyVT/*Hdh*^*Q111/Q111*^ cells was decreased upon Trastuzumab treatment (Fig 7A; PyVT/*Hdh*^*Q111/Q111*^ normalized to 100% and PyVT/*Hdh*^*Q111/Q111*^/Trastuzumab: 79% ± 6%, four independent immunoblotting experiments of at least three samples, PLSD Fisher test *p* < 0.05).

We addressed the effect of Trastuzumab in functional assays. Trastuzumab had no effect on the migration of wild-type cells while it decreased the mean velocity of motile PyVT/*Hdh*^*Q111/Q111*^ cells (Fig 7C). Similarly, Trastuzumab decreased the directed cell migration capacity and invasiveness of PyVT/*Hdh*^*Q111/Q111*^ cells (Boyden chamber assays, Fig 7D; matrigel invasion, Fig 7E). In these assays, Trastuzumab had no effect on PyVT/*Hdh*^*Q7/Q7*^ cells behaviour. These data show that the Trastuzumab-induced reduction of ErbB2 levels in PyVT/*Hdh*^*Q111/Q111*^ cells counteracts the effect of polyQ-huntingtin on migration and invasiveness.

Finally, we tested the impact of Trastuzumab treatment on cell growth. Cells were treated for 24, 48 and 72 h with 20 µg/ml Trastuzumab. Trastuzumab did not affect cell number in PyVT/*Hdh*^*Q7/Q7*^ cells (Fig 7F). In sharp contrast, the same treatment resulted in a 49% decrease in PyVT/*Hdh*^*Q111/Q111*^ cell number after 24–48 h and a 70% decrease after 72 h. Thus, PyVT/*Hdh*^*Q111/Q111*^ tumour cells appear to be hypersensitive to Trastuzumab as compared to PyVT/*Hdh*^*Q7/Q7*^ cells.

## DISCUSSION

PolyQ-huntingtin is known to induce the activation of the apoptotic machinery in cellular and mouse models and to cause neuronal death (Zuccato et al, 2010). Increased apoptosis is also detected in human HD brains. In contrast, wild-type huntingtin protects against cell death induced by different stressors including polyQ-huntingtin itself, and has anti-apoptotic properties. We suggest here that the reported huntingtin functions depend on the cell type and the cell context being studied as we found that mutant huntingtin conferred resistance to anoikis to cancer cells while it renders them more sensitive to Trastuzumab treatment. Furthermore, expression of polyQ-huntingtin in mammary tumour cells changed their cell fate, as these cells were more prone to adopt a mesenchymal phenotype. Previous studies showed that polyQ-huntingtin interferes with neurogenesis. For instance, Simpson et al observed less hippocampal differentiation in a full-length polyQ-huntingtin mouse model of HD (Simpson et al, 2011). More generally, huntingtin is a positive transcriptional regulator of genes involved in neuronal maintenance, a function that is lost when huntingtin is mutated (Zuccato et al, 2003). Thus, huntingtin stands as a key regulator of the balance between cell differentiation, survival and death in normal, neoplastic and neurodegenerative conditions.

Here, we found a hyper-activation of the Akt pathway triggered by ErbB2/HER2. The Akt protein kinase is at the crossroads of essential cellular processes such as cell growth and survival, proliferation and migration (Brunet et al, 2001; Dillon & Muller, 2010). Akt is activated by IGF-1/phosphatidyl inositol–3-kinase (PI3K) signalling, and deregulation of this signalling contributes to cancer (Castaneda et al, 2010; Dillon & Muller, 2010). In particular, hyper-activation of this pathway is observed in breast cancer and consequently affects cell proliferation, migration, invasion and metastasis. In HD, IGF-1 signalling, Akt activity and subsequent phosphorylation of huntingtin at serine 421 are deregulated as HD progresses (Colin et al, 2005; Gines et al, 2003; Mochel et al, 2007; Pouladi et al, 2010; Saleh et al, 2009; Warby et al, 2005). Mutant huntingtin is a direct target of Akt. Upon IGF-1 activation, polyQ-huntingtin is phosphorylated at serine 421 by Akt (Humbert et al, 2002) and this phosphorylation event restores the capacity of polyQ-huntingtin to stimulate vesicular transport along microtubules, as does the wild-type protein (Zala et al, 2008). Thus, Akt-mediated phosphorylation of mutant huntingtin completely inhibits its toxicity in cellular and mouse models of HD (Humbert et al, 2002; Pardo et al, 2006). Future experiments should test whether polyQ-huntingtin could also be one of the downstream targets of Akt in cancer that mediates its effect on tumour formation, cell motility and invasiveness.

The ErbB2/HER2 transmembrane tyrosine kinase is overexpressed in a number of human cancers (Roepstorff et al, 2008). ErbB2/HER2 has no known ligands but is the preferred dimerization partner for the other family members. Two models have been proposed for ErbB2/HER2 endocytosis [reviewed in (Roepstorff et al, 2008; Sorkin & Goh, 2008)]. ErbB2/HER2 dimers were shown to be endocytosed and mostly recycled back to the membrane for reactivation. Alternatively, ErbB2/HER2 dimers were reported to be retained to the cell surface as ErbB2/HER2 is endocytosis impaired. The Hsp90 chaperone machinery is a regulator of ErbB2/HER2 stability and inhibition of this regulation by Geldanamycin allows investigation into the status of ErbB2/HER2 at the membrane and its internalization (Citri et al, 2004). Austin and colleagues reported that Geldanamycin enhances specifically the degradative sorting in endosomes with no effect on the initial endocytosis from the plasma membrane (Austin et al, 2004). In contrast, another study showed that Geldanamycin-induced downregulation of ErbB2/HER2 is dynamin- and clathrin-dependent, and independent of proteasomal activity (Pedersen et al, 2008). In our experimental conditions, the HER2 accumulation in HD conditions was dynamin-dependent in non-stimulated and Geldanamycin-treated SKBr3 cells. Huntingtin is distributed primarily in the cytoplasm but is also found in membrane fractions; it also localizes to endosomes and plasma membranes and interacts with proteins involved in membrane trafficking (DiFiglia et al, 1995; Kegel et al, 2005; Velier et al, 1998). We now provide a direct link between huntingtin and endocytosis by confirming that endogenously expressed huntingtin binds directly to dynamin (Kaltenbach et al, 2007). In HD, the binding is enhanced thereby displacing dynamin. The effect of mutant huntingtin could thus occur at the level of endocytosis leading to HER2 accumulation at the plasma membrane. As HER2 is constitutively available for dimerization with all other family members, this accumulation is sufficient to prolong downstream signalling and promote migration. Indeed, polyQ-huntingtin-induced cell migration is inhibited by Trastuzumab. Furthermore, confirming the central role of HER2 in polyQ-mediated effects, PyVT/*Hdh*^*Q111/Q111*^ tumour cells are hypersensitive to Trastuzumab as compared to PyVT/*Hdh*^*Q7/Q7*^ cells.

The ErbB receptor tyrosine kinases induce several signalling pathways that play central roles during development, in adults, and during disease processes (Eccles, 2011; Yarden & Sliwkowski, 2001). The receptors form homodimers and heterodimers and the way they assemble constitutes one layer of regulation of the network. Indeed, a specific dimer triggers a specific cellular response ranging from apoptosis to proliferation and cell adhesion to metastasis. Several reports have linked mutant huntingtin to abnormal epidermal growth factor receptor (EGFR/ErbB1) signalling. Mutant huntingtin inhibits the activation of Akt induced by epidermal growth factor stimulation in rat pheochromocytoma cells (Song et al, 2002). While huntingtin and EGFR are found in the same complex (Liu et al, 1997), the mechanisms by which mutant huntingtin induced the defects observed remain to be shown. In *Drosophila*, the polyQ-huntingtin-induced accumulation of the single *Drosophila* glutamate transporter dEATT1 is regulated by EGFR (Lievens et al, 2005). In their model, the authors expressed short huntingtin fragments that form massive aggregation that could physically block EGFR signalling at several levels. Future experiments should thus further elucidate whether huntingtin interferes with the dynamin-dependent endocytosis of one or several ErbB receptors. This does not exclude the possibility that, as previously suggested (Liu et al, 1997), huntingtin could also directly bind to these receptors with an altered binding when huntingtin is mutated.

Our data were somewhat unexpected since two studies showed an overall decreased risk of cancer in patients with HD (Ji et al, 2012; Sorensen et al, 1999). At the molecular level, the authors speculated that their findings would be linked to the apoptotic properties of polyQ-huntingtin. While the incidence of cancer is lower, we propose here that the progression could be enhanced in HD. We also found an inverse correlation between the length of the abnormal CAG expansion and the age of breast cancer onset, suggesting that the CAG length in huntingtin may be a prognostic factor. In the study from Ji and collaborators (Ji et al, 2012), the CAG repeat length was not available. Instead, the authors used age at diagnosis as a surrogate and did not find a relation with risk of cancer. Nevertheless, we describe here polyQ-induced abnormalities in HER2 endocytosis in breast cancer cells with consequences on their motility and metastatic behaviour. This may apply only to a subset of cancer types, and this could be the case for breast cancer initiated by HER2 accumulation. Together, our data should not only trigger epidemiological studies on breast cancer in the HD population but may also sensitize clinicians and HD families to specific follow-up that will positively affect the quality of life of HD patients.

Finally, there is an urgent need for an effective treatment for HD, that will either slow down or halt the progression of neuronal dysfunction and degeneration. The development of such therapies is based on a sound understanding of the aetiology and pathogenesis of this disease. Our study leads us to propose that understanding polyQ-huntingtin-induced changes in peripheral tissues could give valuable insight into several pathways involved in neurodegenerative and cancer conditions, including those regulating the ErbB receptor tyrosine kinases family.

## MATERIALS AND METHODS

### Antibodies and DNA constructs

The antibodies used in this study are as follows: huntingtin (PQ-1C2-as, 1C2, Euromedex; HU-4C8-As, 4C8, Euromedex and D7F7, Cell Signaling Technologies); cleaved caspase-3 (9661S, Cell Signaling Technologies); PCNA (PC10, Santa Cruz); Ki67 (NCL-ki67p, Leica); E-cadherin (610182, BD Transduction Laboratories); β-Catenin (ab6302, Abcam); α-SMA (A5228, Sigma); ZO-1 (610966, BD Transduction Laboratories); vimentin (V6630, Sigma); α-tubulin (T6199, Sigma); PyVT (sc-53481, Santa Cruz); HER2 (Ab2428, Abcam); phospho-Akt (4058, Cell Signaling Technologies); Akt (9272, Cell Signaling Technologies); cadherin 11 (ab52891, Abcam); MMP3 (ab53015, Abcam); BARD1 (sc-11438, Santa Cruz); cyclin D2 (ab3085, Abcam); HER2-APC (340554, BD biosciences); dynamin I (sc-12724 m, Santa Cruz); dynamin I/II (2342, Cell Signaling Technologies); IgG mouse (12-371, Upstate biotechnologies). Secondary antibodies were anti-mouse/rabbit/rat antibodies conjugated to Alexa 488, Alexa 555 and Cy5 (immunofluorescence) or to HRP (immunoblotting; Invitrogen).

pARIS-mCherry-httQ23 (Htt Q23) and pARIS-mCherry-httQ100 (Htt Q100) were described in (Pardo et al, 2010). Plasmids encoding wild-type dynamin 2 (Dyn2 WT-GFP) and a GTPase-defective mutant K44A dynamin 2 (Dyn2 K44A-GFP) are a gift of S. Miserey-Lenkei (Institut Curie).

### Immunoprecipitation

Immunoprecipitation experiments were performed on primary PyVT tumour cells. Briefly cells were lysed in IP lysis buffer (50 mM Tris–HCl, pH 7.5; 137 mM NaCl; 1% Triton X-100; 10 mM MgCl_2_; 10% glycerol; 1% protease inhibitor cocktail, Sigma). One milligram of total protein extract in a total volume of 500 µl was incubated with 6 µg of 4C8 or IgG mouse antibody for 2 h followed by 1 h of incubation with 30 µl of protein A/G beads mix. Beads are washed three times in lysis buffer and then eluted in 50 µl of 1× laemelli buffer by heating at 95°C for 10 min.

### Boyden chamber assays

Matrigel invasion assays were performed in matrigel Boyden chambers (354483, BD Biosciences). 3 × 10^4^ serum-starved cells were loaded in media containing 0.1% serum in the insert while the well was filled with media containing 10% serum. After 18 h, chambers were fixed and stained in 0.5% crystal violet (in 20% methanol). Images were taken using a light microscope and cells were counted. For Boyden chambers assays (353097, BD Biosciences), 5 × 10^3^ cells were loaded per insert, chambers were fixed 6 h after.

### Carmine aluminium whole mount staining

For whole mount analysis, the fourth pairs of mammary gland fat pads were dissected from female mice. Fat pads were spread on slides and fixed overnight in a methacarn solution containing 60% methanol, 30% chloroform and 10% acetic acid. Samples were then washed in 100% methanol before rehydrating and subsequent overnight staining in carmine alum solution (StemCell Technologies). Samples were dehydrated, de-stained in xylene and scanned using an Epson Perfection 3200 scanner.

### Cell culture

Cells were cultured in humidified incubators at 37°C and 5% CO_2_. SkBR3 cells (ATCC) were maintained in McCoy's 5A, GlutaMAX media (Gibco) supplemented with 10% foetal calf serum, 100 units/ml penicillin/streptomycin (Gibco). Cells were treated when indicated with Geldanamycin (Sigma) and Trastuzumab (Herceptin®, Roche). Primary cells from MMTV-PyVT/*Hdh*^*Q111/Q111*^ or MMTV-PyVT/*Hdh*^*Q7/Q7*^ tumours were cultured as described (Dangles-Marie et al, 2007) maintained at 8% CO_2_ in DMEM supplemented with 10% foetal calf serum, 10 mmol/L HEPES buffer (Sigma), 1 mmol/L Sodium Pyruvate (Sigma), 100 units/ml penicillin/streptomycin (Gibco), 250 ng/ml Fungizone (Gibco). At least three different cell lines per genotype were generated from independent tumours. For the first three passages 200 µg/ml Gentamicin (Gibco) was added to the media. Transient transfections were performed using lipofectamine LTX (Invitrogen) according to supplier recommended procedures.

### Cell motility

Random migration experiments were carried out in primary tumour cells. Cells in plastic six-well plates were imaged over at least 6 h using an inverted fluorescent 2D Leica DM IRB microscope with photometric CoolSNAP fx camera in a chamber with controlled temperature and CO_2_ conditions and a moving stage. Dividing cells were excluded from the analysis. To access the effect of Trastuzumab on cell motility, cells where imaged 12 h after treatment with 20 µg/ml of Trastuzumab (Herceptin®, Roche). The cell tracking plug-in of ImageJ software (http://rsbweb.nih.gov/ij/plugins/track/track.html) was used to calculate parameters of cell motility.

### Flow cytometric analysis

For anoikis experiments, 5 × 10^5^ cells were cultured in suspension for 16 h (37°C; 5% CO_2_). Cells were stained with annexin V-FITC and/or propidium iodide (556547, BD Biosciences). Cytometric analysis was performed on FACsCalibur (Becton Dickinson). Fifteen thousand events were analysed using CellQuest (Becton Dickinson) and FlowJo software.

To study the levels of ErbB2 at the membrane SkBR3 cells were transfected with different constructs. Twenty-four hours after transfection cells were harvested and stained with HER2-APC antibody according to manufacturer instructions. Cytometric analysis was performed on FACSAriaIII (BD Biosciences), 10,000 events per condition were analysed using FlowJo software. Mean-APC fluorescence levels were quantified in cells expressing either Htt Q23 or Q100 (mCherry positive, gated 10^3^ to 10^5^) and/or dynamin 2 constructs (FITC positive, gated 10^4^ to 10^5^). Histograms were created using FlowJo software.

### Immunofluorescence

Cells were fixed in cold methanol and stained for the presence of E-cadherin (1:500), β-catenin (1:1000), HER2 (1:100) and dynamin (1:50) as indicated. For microscopic analysis of mammary tumours, tumours were dissected and fixed in AFA (75% ethanol, 5% glacial acetic acid and 20% of 4% paraformaldehyde in PBS 1×) for 2–3 h at RT. Tumour samples were cryoprotected in 30% sucrose (in PBS 1×), included in occipitocervicothoracic (OCT) and stored at −80°C until processing. Samples were processed for immunofluorescence imaging by cryostat sectioning (8 µm) and adhering to Thermo Scientific Superfrost glass slides. Slides were then stained for the presence of β-Catenin (1:200), E-Cadherin (1:2000), α-SMA (1:500) and ErbB2 (1:100). Briefly, slides were washed in PBST (PBS1×, 0.001% tween20); de-masked in citrate buffer 10 mM pH6.0 (90°C, 20 min), blocked in 5% goat serum; stained overnight at 4°C; washed in PBST and incubated with Alexa fluorescent secondary antibodies (RT, 30 min). Slides were washed in PBST and mounted in Fluoroshield with DAPI. Imaging was carried out using a Leica TCS-SP5 confocal microscope with 40× or 63× objective lens.

### Mice

FVB/N-Tg(MMTV-PyVT)634Mul/J and FVB-Tg(MMTV-Erbb2)NK1Mul/J mice were obtained from The Jackson Laboratory. *Hdh*^*Q111/Q111*^ mouse line (CD1 background) have been described earlier (Wheeler et al, 1999). For tumour-free survival assays, mice were surveyed for tumour appearance by palpation. Spontaneous tumours dissected in small pieces (8 mm^3^) were grafted in the right flank of 6-week-old nude mice (Charles river laboratories: Crl:Nu(Ico)-Foxn1 nu; Morton & Houghton, 2007). Tumour growth was followed and mice were sacrificed before the largest tumour reached 10% of the body weight. Tumoural progression was determined on mice sacrificed at 6, 8, 12 and 14 weeks of age. Abdominal mammary glands were dissected, stained with carmine aluminium and percentage of tumoural area was evaluated using ImageJ software. Metastasis was analysed on 12-week-old mice dissected lungs. Entire organs were included in paraffin and sectioned (7 µm sections with 100 µm interval; covering the entire lung). The number of metastasis was counted after PyVT staining. Experimental procedures were performed in accordance with the recommendations of the European Community (86/609/EEC) and the French National Committee (87/848) for care.

**The paper explained**

PROBLEM:

Huntington disease (HD) is a severe neurodegenerative disorder caused by an abnormal polyglutamine expansion (polyQ) in the huntingtin protein. Given the adult onset and the characteristic neuronal dysfunction and death of adult neurons in HD, most studies have focused on the toxic pathways activated by polyQ-huntingtin in post-mitotic neurons. However, over the last few years, emerging evidence point out that HD is also associated with peripheral manifestations. These symptoms may not be only secondary to the neuronal dysfunctions but linked to the presence of polyQ-huntingtin in the dysfunctioning tissues. Indeed, the protein is ubiquitous and plays critical roles in fundamental biological processes shared by all cells in the organism.

RESULTS:

Here, we investigated whether mutant huntingtin could influence the progression of breast cancer after we found it to be expressed in the mammary epithelium and tumours. We show that mutant huntingtin accelerates tumourigenesis in two mouse breast cancer models, increases epithelial–mesenchymal transition of cancer cells and thus favours lung metastasis in mice. We then report that in HD, the dynamin dependent endocytosis of ErbB2/HER2 receptor tyrosine kinase is reduced leading to its accumulation and, to a subsequent increase in cell motility and proliferation.

IMPACT:

Our findings may have direct implications for the follow-up and care of HD patients and positively affect their quality of life. Furthermore, there is an urgent need for an effective treatment for HD. Our study demonstrates that understanding polyQ-huntingtin-induced dysfunctions in peripheral tissues gives unexpected but valuable insight into pathways involved in neurodegenerative and cancer conditions. Finally, we propose huntingtin to be a general regulator of the balance between cell differentiation, survival and death in normal, neoplastic and neurodegenerative conditions.

### RNA extraction, microarray and real time PCR

Total RNA was isolated from frozen mammary tumours using miRNeasy mini kit (Qiagen). One hundred nanograms of total RNA was amplified with Ambion® WT Expression Kit. cRNA products were monitored using the Nanodrop (Thermo, France) and the Agilent Bioanalyzer. Affymetrix Mouse Exon 1.0 ST arrays were hybridized according to Affymetrix (Santa Clara, USA) labelling and hybridization procedures. Microarrays were hybridized, washed and scanned using Affymetrix instruments. Total RNAs RIN values were between 8.1 and 9.3. Microarrays were hybridized with 4.7 µg of labelled DNA. Raw data are controlled with Expression console (Affymetrix).

RNA samples were retrotranscribed using the First-Strand cDNA Synthesis Kit (Invitrogen). cDNAs were diluted 1:10 and submitted to RT-PCR with 7900HT Fast real time PCR system (Applied biosystems) using power SYBR Green PCR Master mix (Applied biosystems) with the following *htt* (5′-CTCAGAAGTGCAGGCCTTACCT-3′, 5′-GATTCCTCCGGTCTTTTGCTT-3′ and 5′-CTCAGAAGTGCAGGCCTTACCT-3′, 5′-GATTCCTCCGGTCTTTTGCTT-3′; Benn et al, 2008) and *ErbB2* (5′-GAAACCGGACCTCTCCTACA-3′, 5′-CGGAYCYYCYGTCTCCTTCG-3′; Landis et al, 2006) oligonucleotide pairs. *β-actin* (5′-AGGTGACAGCATTGCTTCTG-3′, 5′-GCTGCCTCAACACCTCAAC-3′) and *hprt* (5′-GCTGGTGAAAAGGACCTCT-3′, 5′-CACAGGACTAGAACACCTGC-3′) genes were used as internal controls. Fold changes were calculated using the ddCT method.

### Microarray data analysis

Dataset analysis and visualization were made using EASANA® (GenoSplice technology, http://www.genosplice.com), based on the GenoSplice's FAST DB® annotations. Exon Array data were normalized using quantile normalization. Background correction was made by using the antigenomic probes. Only probes targeting exons annotated from FAST DB® transcripts were selected. Among these selected probes, bad-quality probes (*e.g.* probes labelled by Affymetrix as ‘cross-hybridizing’) and probes with too low intensity signal compared to antigenomic background probes with the same GC content were removed from the analysis. The *p*-values were not corrected for multiple comparisons. Probes with a DABG *p*-value ≤0.05 in at least half of the arrays were considered for statistical analysis. Only genes expressed in at least one compared condition were analysed. To be considered as expressed, the DABG *p*-value had to be ≤0.05 for at least half of the gene probes. Unpaired Student *t*-test was performed to compare gene intensities in the different biological replicates. Genes were considered significantly regulated when fold-change ≥1.5 and *p*-value ≤0.05. GEO accession number: GSE28685.

### Hierarchical clustering

The distance from the gene signal in a given sample to the corresponding average in the eight samples was calculated for each regulated gene. Corresponding values were displayed and clusterized with MeV4.6.2 from The Institute of Genome Research using Pearson correlation and average linkage clustering.

### Statistical analyses

Statview 4.5 software (SAS Institute, Cary, NC) was used for statistical analyses. Data are expressed as means ± SE. Groups were compared by ANOVA followed by unpaired *t* tests or by Fisher's PLSD *post hocs* analyses for multiple comparisons. For Fig 1A, a Kaplan–Meier Analysis was performed. Complete statistical analyses are available in the Supporting Information.

For more detailed Materials and Methods see the Supporting Information.

## Supplementary material

As a service to our authors and readers, this journal provides supporting information supplied by the authors. Such materials are peer reviewed and may be re-organized for online delivery, but are not copy-edited or typeset. Technical support issues arising from supporting information (other than missing files) should be addressed to the authors.
