# Supplementary material for: The Huntington disease protein accelerates breast tumour development and metastasis through ErbB2/HER2 signalling
Source: EMBO Mol Med. 2013 Jan 9;5(2):309–25. doi: 10.1002/emmm.201201546 (PMC3569645; doi:10.1002/emmm.201201546)
Supplement: Supplementary file 1 [file emmm0005-0309-SD1.pdf]

EMBO Molecular Medicine

The Huntington disease protein accelerates breast tumor development and metastasis through ErbB2/HER2 signalling

Cristovão Moreira Sousa, John Russel McGuire, Morgane Sonia Thion, David Gentien, Pierre de la Grange, Sophie Tezenas du Montcel, Anne Vincent-Salomon, Alexandra Durr, Sandrine Humbert

Corresponding author: Sandrine Humbert, Institut Curie

Review timeline:

Submission date:	07 May 2012
Editorial Decision:	11 June 2012
Additional Author Correspondence:	26 June 2012
Additional Editorial Correspondence:	27 June 2012
Additional Author Correspondence:	27 June 2012
Revision received:	21 September 2012
Editorial Decision:	25 October 2012
Appeal Received:	26 October 2012
Editorial Decision:	08 November 2012
Additional Author Correspondence:	12 November 2012
Additional Editorial Correspondence:	13 November 2012
Revision received:	14 November 2012
Editorial Decision:	16 November 2012
Revision received:	19 November 2012
Accepted:	21 November 2012

Transaction Report:

(Note: With the exception of the correction of typographical or spelling errors that could be a source of ambiguity, letters and reports are not edited. The original formatting of letters and referee reports may not be reflected in this compilation.)

1st Editorial Decision 11 June 2012

Thank you for the submission of your manuscript to EMBO Molecular Medicine. We have now heard back from the three referees whom we asked to evaluate your manuscript. Although the referees find the study to be of potential interest, they also raise a number of concerns about the limited mechanistic insight and clinical relevance.

As you will see from the reports below, Referees #1 and #3 are particularly concerned with the limited mechanistic insght and together with Referee #2, require added data and details (both at the text and technical levels) to clarify some issues. It also becomes apparent that the previous results from different studies should be better discussed.

Given these evaluations, I would like to give you the opportunity to revise your manuscript, with the understanding that all the referees' concerns must be fully addressed and that acceptance of the manuscript would entail a second round of review. Please note that it is EMBO Molecular Medicine policy to allow only a single round of revision and that, as acceptance or rejection of the manuscript will depend on another round of review, your responses should be as complete as possible.

Revised manuscripts should be submitted within three months of a request for revision; they will

otherwise be treated as new submissions, except under exceptional circumstances in which a short extension is obtained from the editor. Also, the length of the revised manuscript may not exceed 60,000 characters (including spaces) and, including figures, the paper must ultimately fit onto optimally ten pages of the journal. You may consider including any peripheral data (but not methods in their entirety) in the form of Supplementary information.

I look forward to seeing a revised form of your manuscript as soon as possible.

Yours sincerely,

Editor
EMBO Molecular Medicine

***** Reviewer's comments *****

Referee #1 (Comments on Novelty/Model System):

The data is not convincing at all and there are contradictions inherent in the animal studies
The sample size is not convincing and controls are missing in some studies.

Referee #1 (Other Remarks):

In this study the investigators examined the role of ployQ huntingtin protein in breast cancer. Although the transgenic mouse model of polyQ htt has led to some interesting findings, the results are preliminary and the mechanism by which htt affects tumorigenesiis in the mammary gland has not been well addressed.

Specific comments

1. The data that links polyQ htt with breast cancer in humans is weak. Does breast cancer occur at a higher frequency in HD patients? The sample size in current study (n=12) is too small. It appears that htt staining is much weaker in tumors than in normal samples (fig 1). This is in contradiction with the animal study, in which forced expression of polyQ htt increases the onset of breast tumor.

2. In the study of the mouse model of htt in breast cancer, the data in fig 2A shows the effect of htt on the onset of breast cancer induced by PyVT. The study should also include characterization of the progression of the tumor (size) and metastasis. It is not clear how many mice were used in the study or if the differences are statistically significant (fig 2A).

3. The finding of the increase of HER2 expression in htt tumors is of great potential interest. However, it is not clear if up regulation of HER2 is also found in human HD patients who have developed breast cancer (only 3 of the 12 patients were confirmed to have HER2+ tumors). In addition, it would be of interest to show if HER2 plays a role in the malignant phenotype associated with htt, such as migration and invasion (the measurement of random migration as shown in Fig 6 is not sufficient and not convincing). Does anti-HER2 antibody inhibit tumor onset in the transgenic model?

4. In the microarray study, the expression pattern of the EMT genes should be shown.

5. The mechanism by which htt affects HER2 levels should be explored in more detail. Fig 6c the study of her2 internalization by immnufluorescence is not convincing. The investigators should perform flow cytometry analysis of Her2 levels on the cell surface using an antibody specific for the ectodemain of her2. The study using the Hsp90 inhibitor shows the effect on stability of Her2; however this is not a direct measurement of internalization.

The investigators propose that polyQ htt stabilize Her2 by preventing internalization and degradation of HER2Interestingly, the anti-HER2 antibody can effectively reduce HER2 levels in the ployQ htt tumors. The investigators should address the possible mechanism for this.

6. The data shown in fig 6 do not show whether the anti-her2 antibody affect signalling in polyQ htt tumors, contrary to what is claimed in the text.

Referee #2 (Comments on Novelty/Model System):

Using a variety of approaches, and two mouse models for breast cancer that have been crossed to a knock-in mouse model for Huntington's disease, the authors have provided a comprehensive analysis of the effect of mutant huntingtin expression on the progression of breast cancer tumorigenesis. Their finding that metastasis is enhanced is novel, and is not what most would have predicted based on past analyses of cancer incidence in Huntington's disease patients. This past work showed that cancer incidence was significantly decreased in Huntington's disease. If Sousa et al.'s work is taken at face value, their findings are very important for both physicians and those involved in understanding pathogenic mechanisms in Huntington's disease. The use of two in vivo, and various in vitro models (e.g. matrigel cultures and cell mobility assays) is appropriate for the study.

Referee #2 (Other Remarks):

In this study by Sousa et al., the authors find that in 12 HD patients with breast cancer, progression of the cancer was enhanced. To explore the molecular mechanism(s) responsible for this observation, the authors investigated tumorigenesis in two mouse models for breast cancer that have been crossed with a knock-in mouse model for Huntington's disease. Similar to what was observed in the HD patients with breast cancer, tumorigenesis in the mice expressing mutant htt was accelerated and the epithelial-mesenchymal transition of the cancer cells, a phenotype that can contribute to lung metastasis in the mice, was enhanced. Additional studies in the mouse models provided evidence that the accerlerated progression of the breast cancer was due, in part, to hyperactivation of the ErbB2/HER2 signalling pathway.

These studies represent the first attempt to understand how mutant huntingtin expression could influence cancer progression. The majority of studies investigating mutant huntingtin pathogenesis have focused on the central nervous system, despite the fact that huntingtin is expressed throughout the body. Sousa et al.'s results with regard to mutant huntingtin and cancer progression are in stark contrast with the results of a study by Sorensen et al., 1999, and a more recent study by Ji et al., 2012 that suggested that the overall incidence of cancer was lower in Huntington's disease patients, and in patients with other expanded polyglutamine disorders. Although the mechanism(s) responsible for these findings was never explored further, the authors suggested that the effect of mutant huntingtin on cancer risk could be due to its apoptosis-promoting activity, and its ability to upregulate p53 expression. Interesting, Sousa et al. find an inverse correlation between the length of the expanded polyglutamine stretch and the age of breast cancer onset. A similar analysis in the Ji et al. study was not performed because CAG repeat length was not available for all patients, and instead, age-at-onset of Huntington's disease symptoms was used as a surrogate. When this parameter was used, the authors could find no correlation between HD age-at-onset and cancer incidence. Nevertheless, Sousa et al. demonstrate convincingly that the observed alterations in HER2 signalling caused by mutant huntingtin expression can affect both tumor cell mobility and metastasis. These findings are important for the field, as they can inform physicians about the necessity for follow-up in Huntington's disease patients with cancers, and they also provide new clues about deficits in signalling pathways, and potential developmental alterations (due to changes in the EMT) that could occur in individuals at risk for Huntington's disease.

There are, however, a few minor concerns, which if addressed, could improve the manuscript.

1. In the discussion, the authors state that their results neither confirm nor invalidate the Sorensen et al., 1999 study. This is a fair statement, but the authors should explore a comparison of their results with Sorensen et al. further in the discussion, as this issue will likely interest many readers. In addition, the authors should cite the recent Ji et al. study (The Lancet, published online April 12, 2012, DOI:10.1016/S1470-2045(12)70132-8) in their manuscript. Interestingly, Ji et al. find that the incidence of benign tumors is also decreased in Huntington's disease patients. If taken at face value, this result would suggest that differences in metastasis likely do not contribute to a reduced incidence of tumorigenesis in Huntington's disease patients, a conclusion that would be compatible with Sousa et al.'s study, and is also compatible with the idea that although cancer incidence may be

© EMBO

reduced, progression may be enhanced in some cancers.

2. To confirm their gene microarray studies, the authors assessed the mRNA and protein levels for a subset of genes, including Hdh. The authors state that mutant huntingtin mRNA was lower than that for wild-type huntingtin mRNA, but it is difficult to find this data in Fig. S2. It would help the reader to show this in a plot, especially because it is not clear that the western data for mutant huntingtin levels can be taken at face value. The authors apparently used the 4C8 antibody whose eptiope is huntingtin amino acids 443-457 (Cong et al., 2005, Hybridoma, 24(5):231-5.). Do the authors know if this antibody recognizes huntingtin phosphorylated at S421 or acetylated at K444 equally as well as un-modified huntingtin? If not, the signal obtained with this antibody may not represent all the huntingtin that is present. Other antibodies that are insensitive to post-translational modifications could be used (e.g. PW0595, anti-N2-17 from ENZO). Alternatively, a recent study by Di Pardo et al., PNAS, 2012, 109(9):3528-3533 describes SDS-PAGE and western blot transfer conditions that increase substantially the amount of mutant huntingtin that can be detected in protein extracts.

Referee #3 (Comments on Novelty/Model System):

This paper is interesting and important, if the comments are well responded. Model systems used were adequate.

Referee #3 (Other Remarks):

In this study, the authors tested the effect of huntingtin gene mutation on the progression of breast cancer. First, it was suggested that breast cancer phenotypes are worse in HD patients compared to non-HD population. Then, the authors examined the genetic interaction between huntingtin mutation and two different models of breast cancer and found that homozygotes for huntingtin mutation were associated with more aggressive phenotypes of mammary tumors. Accumulation of ErbB2 in mutant huntingtin-containing tumors was proposed as a potential mechanism for the aggressiveness. Overall, this study includes some interesting observations related to peripheral effects of the huntingtin mutation. Even though the mechanism remains largely unclear, the results of model animal experiments seem to show the genetic effect of huntingtin mutations on the aggressiveness of breast tumors. However, the reviewer has some cconcerns on the manuscript.

Major points
- Even though two aspects of huntingtin mutation, loss of normal function and gain of toxic function, are emphasized throughout the text, it is still unclear whether the accelerated phenotypes in HdhQ111/Q111 tumors are explained by gain or loss of function of huntingtin (or both). Whereas knock-in animals are accurate genetic models of HD, it is difficult to separate the effects of gain and loss of function in these models, especially when comparing wild type and mutant homozygotes. In the text, the authors seem to presume the gain of function mechanism, which has not been directly examined. Only the experiments in Fig. 6C partially addressed this question, though the output was HER2 internalization but not the aggressive phenotypes of cancer cells. The reviewer requests the authors to compare the cellular phenotypes with or without RNAi-mediated knockdown of huntingtin in both HdhQ7/Q7 and HdhQ111/Q111 tumors.
- While the authors propose ErbB2/HER2 elevation as a mechanism of exacerbation of breast cancer from the animal study, only 3 out of 6 examined HD patients have been confirmed to show the HER2+ phenotype (Fig. 1A).Given the small number of sample size, it is difficult to conclude at this moment that breast cancer in human HD is exacerbated though a HER2-related pathway. One concern is that this pathway is only relevant to the tumors of the HdhQ111/Q111 mice. At least, it should be tested whether the heterozygous HdhQ7/Q111 tumors show elevated ErbB2 compared to HdhQ7/Q7 mice.

Minor points
- How many HD cases were examined to find the patients with breast cancer? The incidence is high?
- Fig 1B: The authors used rank order for both axes. If they use exact numbers of the repeat and age, is there significant correlation?

- Fig.1C. the reviewer agrees that this staining pattern is suggestive of the expression of huntingtin probably including mutant one. However, 4C8 antibody recognizes both wild type and mutant huntingtin and it is difficult to distinguish their expression patterns. It is suggested to modify the sentence (page 7, line 1-2), or examine the sections with antibody that specifically recognizes mutant, but not normal, huntingtin.
- The authors found no obvious increase in ErbB2 mRNA levels in wt and polyQ-htt mammary tumors. However, microarray data is not sufficient to exclude the effect of transcriptional level. qRT-PCR should be done for confirming this point.
- Microarray analysis. Were the p-values corrected for multiple comparisons or not? In either case, this point should be mentioned in the Materials and Methods.
- In figure 6D, Immunofluorescent data of HER2, E-cad, beta-catenin in Trastuzumab-treated cells should be added.
- In figure 6F, why there is no increase even in HdfQ7/Q7 cells? Does Trastuzumab suppress cell growth of these cells?

Additional Author Correspondence 26 June 2012

We thank you for giving us the opportunity to send a substantially revised version of our manuscript EMM-2012-01546 entitled ´ Huntington disease protein accelerates breast tumor development and metastasis through ErbB2/HER2 signalling[a]. However, I am writing to you after carefully reading your letter and the comments of the referees on our study to ask your opinion about a specific point raised by one of the reviewer.

Indeed, I do have a concern regarding one proposed experiment: While we will provide the exerimental data for the questions raised in point 3 of reviewer 1, in the last lane of his concern the reviewer asks: "Does anti-HER2 antibody inhibit tumor onset in the transgenic model". This is clearly something we would have aimed to perform during our study, however, there are several technical issues. Indeed, while herceptin/trastuzumab has been widely used to interfere with tumor progression in xenografts experiments, to our knowledge its use in a PyVT-based transgenic mouse models has rarely (never?) been reported. Thus, such experiments would need several tests. We would need to evaluate the duration of the treatment (we anticipate it to be longer than in grafts experiments), then the toxicity of the treatment. We may have to use other molecules than trastuzumab. Indeed, trastuzumab corresponds to the humanized form of the antibody, that is tolerated by immunodeficient mice (as used in the graft experiments) but may not apply to transgenic mice. For each of these points, we will need to generate the adequate number of mice by crossing our different mouse models. This will definitely take more than the 3 month length of the review process (we anticipate 12 month minimum) rendering it beyond the scope of the current study.
Apart from this, we will be able to answer to all of the concerns by additional experiments or clarifications. In particular we will:

1) Provide mechanistic information on how mutant huntingtin affects Her2 levels. We started to address this point during the review process and find that mutant huntingtin interferes with dynamin-dependent Her2 internalization. We will further describe this mechanism using flow cytometry and cell biology approaches.
2) Add the additional controls required. This includes tumor progression and metastasis in the PyVT model, huntingtin RNA levels and huntingtin protein levels using several antibodies in tumors, detailed analysis of the effect of herceptin on signalling and invasion.
3) Discuss the Ji et al. study (Lancet). We particularly apologize for this. At the time of the submission (May), we missed this publication and had access to it only during the review process.

In conclusion, we strongly believe that addressing the above mentionned points should strengthen our manuscript and bring solid data on the influence of mutant huntingtin during the progression of breast cancer.

I would be happy to have your opinion about the issue of treating the transgenic mice with herceptin.

Additional Editorial Correspondence 27 June 2012

Thank you for your e-mail.

I do understand your concern regarding the time it would take to appropriately answer point 3 of Referee 1. May I suggest that you explain this issue in writing in the point-by-point response to the reviewers. In addition, you may try to improve figure 6 in providing a more convincing evidence for the effect of Trastuzumab on cell migration in order to at least partially answer this reviewer's concern.

As for the rest of the issues, and your point 1 in particular, increasing the mechanistic insights as suggested by the referees is particularly important and together with addressing the rest of the issues as you suggested, will certainly improve your manuscript.

I am looking forward to a revised form of your article.

Yours sincerely,

Editor
EMBO Molecular Medicine

Additional Author Correspondence 27 June 2012

Thank you for your comments.

We will provide convincing evidence for the effect of Trastuzumab on cell migration and invasion. Furthermore, we are focusing most of our efforts to increase the mechanistic insights of our study.

We believe, that this should definitely improve our manuscript.

1st Revision - authors' response 21 September 2012

Referee #1

Comments on Novelty/Model System:

The data is not convincing at all and there are contradictions inherent in the animal studies. The sample size is not convincing and controls are missing in some studies.

Other Remarks:
In this study the investigators examined the role of ployQ huntingtin protein in breast cancer. Although the transgenic mouse model of polyQ htt has led to some interesting findings, the results are preliminary and the mechanism by which htt affects tumourigenesis in the mammary gland has not been well addressed.

Specific comments

1. The data that links polyQ htt with breast cancer in humans is weak. Does breast cancer occur at a higher frequency in HD patients? The sample size in current study (n=12) is too small.
The initial goal of this study was not to establish the incidence of breast cancer in HD but to define whether polyQ huntingtin can affect cancer progression. Indeed, two studies show that the overall

incidence of cancer is lower in HD patients (Ji et al, 2012; Sorensen et al, 1999). We are now discussing this point in detail p. 20 of the revised manuscript.
Our data show a relationship between earlier ages of breast cancer onset and longer CAG repeats ($r_{spearman}$= -0.62, p-value = 0.03)(Fig 1B). We also find that mutant huntingtin is expressed in human breast tumors. Furthermore, we provide compelling evidence that mutant huntingtin influences cancer cell motility and metastasis. Together, this study should trigger epidemiological studies on breast cancer in the HD population.
In conclusion, while the overall incidence of cancer is lower in HD patients, we can propose from our data that cancer progression may be enhanced in HD. Mutant huntingtin could have opposite effects on tumour appearance and EMT. This could be correlated with the CAG expansion. Finally, the increased severity may apply to some cancers only: for instance, this could be the case of breast cancer initiated by a HER2 accumulation.
For the referee interest: we have started a clinical study aiming at systematically investigating cancer in HD patients, in particular breast and ovarian cancer. The major goal of this study is not to analyze the incidence (this was examined in the above cited studies), rather we propose to examine whether the type and severity of cancer developed by HD patients are modified compared to the non HD population. We will have data on the size of the CAG expansion and will investigate whether there is a relation between the expansion size and the risk/severity of breast cancer.

It appears that htt staining is much weaker in tumours than in normal samples (fig 1).
In HD case 1, huntingtin staining appears weaker in tumours than in normal samples. However, the sample size for the immunohistochemistry in human samples is very small and it is thus difficult to conclude. Up to now, there was no data reporting the presence of mutant huntingtin (and wild-type huntingtin) in breast. The goal of Fig 1C was to report the expression in human HD tissues of huntingtin in mammary normal residual tissue and tumoural tissue. We used an antibody that recognizes both wild-type and mutant huntingtin. We have now completed these data by using a mutant specific antibody (as requested by referee 3, minor point 3). In particular, we confirmed that in the samples analysed, mutant huntingtin is enriched in the nucleus (Fig 1D).
We are not trying to draw any conclusions regarding the relative expression level and localization of wild-type and mutant huntingtin in normal versus tumoural tissues based on the analysis of such limited number of samples. We simply conclude that mutant huntingtin is expressed in breast tumours. This warrants the interest of studying its influence during breast cancer progression.

This is in contradiction with the animal study, in which forced expression of polyQ htt increases the onset of breast tumour.
Regarding the HD mouse model used, we feel that there is a misunderstanding: this model does not correspond to a forced expression of polyQ huntingtin in a particular tissue. Rather, the $Hdh^{Q111/Q111}$ mouse line carries an abnormal 111 CAG repeat expansion in the huntingtin gene encoding an abnormally expanded polyQ stretch in huntingtin (Wheeler et al, 1999). This mouse line was created by replacing the wild-type exon 1 of mouse huntingtin with a human mutant exon with the mouse endogenous *htt* promoter. Mutant huntingtin is therefore expressed in appropriate tissues (as the wild-type is) and at endogenous levels. Thus, it is not appropriate to say that there is a forced expression of polyQ huntingtin. In fact, as revealed by microarrays and immunoblotting experiments (text p. 9; Fig 3E; Fig S3A), the levels of mutant huntingtin are even lower than the wild-type huntingtin levels.
As stated above and suggested by the reviewer him/herself, as we can not draw conclusions about the levels/localization of mutant huntingtin in the human samples, the observations can not be in contradiction with the mouse data.

2. In the study of the mouse model of htt in breast cancer, the data in fig 2A shows the effect of htt on the onset of breast cancer induced by PyVT. The study should also include characterization of the progression of the tumour (size) and metastasis.
As requested by the reviewer, we have included the characterization of tumour progression (Fig 2C) and metastasis (Fig 5E). In the PyVT breast cancer model and in particular in the genetic background used, tumours initiate at several sites rendering the analysis of tumour size per se not reliable. Instead, we evaluated tumour progression as the tumoural area/total mammary gland area (percentage of tumoural tissue; at least n=3 per genotype). We now clearly show that tumour progression and metastasis are increased in MMTV-PyVT/$Hdh^{Q111/Q111}$ mice as compared to MMTV-PyVT/$Hdh^{Q7/Q7}$ mice.

It is not clear how many mice were used in the study or if the differences are statistically significant (fig 2A).
The number of mice used as well as the detailed statistical analyses were described in the supporting information of the previous version of the manuscript. We now have included the number of mice used and statistical significance of the experiments (p values) in the figure legends. The complete statistical analyses are in the supporting information.
In particular for figure 2A: Tumor free survival data are from paired littermates from twelve different litters. MMTV-PyVT/*Hdh*$^{Q7/Q7}$: 19 mice; MMTV-PyVT/*Hdh*$^{Q7/Q111}$: 37 mice; MMTV-PyVT/*Hdh*$^{Q111/Q111}$: 25 mice. Kaplan-Meier Analysis, Logrank Test: p-value < 0.0001 Chi-2 = 22.60.

3. The finding of the increase of HER2 expression in htt tumours is of great potential interest. However, it is not clear if up regulation of HER2 is also found in human HD patients who have developed breast cancer (only 3 of the 12 patients were confirmed to have HER2+ tumours).
The HER2 status was established by immunohistochemistry following the American Society of Clinical Oncology recommendations as documented in (Wolff et al, 2007). We had access to the phenotypic characterization of the tumour for 6 patients, three of them were HER2-positive (ErbB2/neu).
We did not have the molecular information for the other HD patients. We acknowledge that the sampling is too small. A complete study of the HER2 status in HD/breast cancer requires further investigation. It is the topic of an on-going study (involving several HD clinical centres in France).

In addition, it would be of interest to show if HER2 plays a role in the malignant phenotype associated with htt, such as migration and invasion (the measurement of random migration as shown in Fig 6 is not sufficient and not convincing).
In the new Fig 8C, 8D and 8E, we show that targeting HER2/ErbB2 using Trastuzumab reduces the increased random migration, directed migration (Boyden chambers) and invasion associated with mutant huntingtin.

Does anti-HER2 antibody inhibit tumour onset in the transgenic model?
This is clearly something we aimed to perform during our study. However, there are major technical issues.
While herceptin/Trastuzumab has been widely used to interfere with tumour progression in xenograft experiments, its use in a PyVT-based transgenic mouse models has never (to our knowledge) been reported. Thus, such experiments would need several tests. We would need to evaluate the duration of the treatment (we anticipate it to be longer than in graft experiments), then the toxicity of the treatment. We may have to use other molecules than Trastuzumab. Trastuzumab corresponds to the humanized form of the antibody, that is tolerated by immunodeficient mice (as used in the graft experiments) but may not apply to transgenic mice. For each of these points, we would need to generate the adequate number of mice by crossing our different mouse models.
More importantly, to address the effect of Trastuzumab on tumour onset (as requested by the referee) one would have to treat young mice (4 weeks, given the severity of our model). This may be associated with toxic effects. The other option would be to assess the effect of Trastuzumab on tumour growth, when all the tumours reach the same size. However, the PyVT breast cancer model is not appropriate for this, as tumours initiate at several sites.
Together, we believe that assessing the effect of Trastuzumab in the transgenic model or more generally *in vivo* in a HD situation is beyond the scope of the current study.

4. In the microarray study, the expression pattern of the EMT genes should be shown.
As requested, we have included a new table, Table S3. We compared our microarray data to a multi-cancer stage associated gene expression signature enriched in EMT markers (Cheng et al, 2012; Kim et al, 2010). Among the 64 genes corresponding to the top 100 probe sets of the signature, we found 31% of them to be significantly up regulated in HD conditions.

5. The mechanism by which htt affects HER2 levels should be explored in more detail.
Fig 6c the study of her2 internalization by immunofluorescence is not convincing. The investigators should perform flow cytometry analysis of Her2 levels on the cell surface using an antibody specific for the ectodemain of her2. The study using the Hsp90 inhibitor shows the effect on stability of Her2; however this is not a direct measurement of internalization.

The investigators propose that polyQ-htt stabilize Her2 by preventing internalization and degradation of HER2. Interestingly, the anti-HER2 antibody can effectively reduce HER2 levels in the polyQ htt tumours. The investigators should address the possible mechanism for this.
In the revised version of the manuscript, we explored the underlying mechanisms by which huntingtin affects HER2 levels. We used a combination of different approaches. As discussed previously, we take advantage of the SKBr3 cell line, a widely used system in which HER2 internalization can be induced using Geldanamycin. As suggested by the reviewer, we also used flow cytometry to analyse HER2 levels at the cell surface. Furthermore, we performed immunostaining and immunoprecipitation experiments (Fig 7 of the revised manuscript; corresponding text pages 12 to 15). We now show that in HD, HER2/ErbB2 accumulates at the cell surface in HD conditions and polyQ-huntingtin interferes with its dynamin-dependent endocytosis. This occurs through the abnormally strong interaction of huntingtin to dynamin when huntingtin contains the polyQ mutation.

Briefly, we first showed that the decrease of HER2 staining at the membrane triggered by Geldanamycin treatment is less efficient in the presence of polyQ-huntingtin than when wild-type huntingtin is expressed (Fig 7A). We then specifically addressed the effect of mutant huntingtin on internalization by flow cytometry analysis of HER2 levels at the cell surface. Upon Geldanamycin treatment, the internalization of HER2 in wild-type cells is greater than in HD cells (Fig 7B).

We then confirmed a previous study showing that Geldanamycin induces endocytosis-based degradation of ErbB2 through a dynamin dependent mechanism (Pedersen et al, 2008)(Fig 7C) and asked whether the effect of mutant huntingtin could be dynamin dependent. By co-expressing wild-type dynamin with wild-type and mutant huntingtin, we observed that dynamin leads to a decrease of HER2 at the cell surface and this effect is partially blocked in cells expressing mutant huntingtin (Fig 7D).

Huntingtin was shown to interact with dynamin by yeast two hybrid (Kaltenbach et al, 2007). We found that indeed dynamin interacts with huntingtin in cancer cells, and the interaction is greatly enhanced when huntingtin contains an abnormally expanded polyQ stretch (Fig 7E). At the subcellular level, in the mutant situation the characteristic localization of dynamin near the plasma membrane is lost with dynamin being dispersed throughout the cytoplasm (Fig 7F). We conclude that the stronger interaction of mutant huntingtin and dynamin leads to a redistribution of dynamin and a subsequent decreased endocytosis of HER2/ErbB2.

6. The data shown in fig 6 do not show whether the anti-her2 antibody affect signalling in polyQ htt tumours, contrary to what is claimed in the text.
We apologize for the initial overstatement. We now show in Fig 8A that polyQ-huntingtin leads to ErbB2 accumulation and Akt activation that are inhibited when cells are treated with Trastuzumab. Trastuzumab inhibits polyQ-huntingtin induced HER2 accumulation (PyVT/$Hdh^{Q111/Q111}$: 100% and PyVT/$Hdh^{Q111/Q111}$/Trastuzumab: 41% ± 14%, 4 independent immunoblotting experiments of at least 3 samples, PLSD Fisher test $p < 0.05$) and Akt signalling (PyVT/$Hdh^{Q111/Q111}$: 100% and PyVT/$Hdh^{Q111/Q111}$/Trastuzumab: 79% ± 6%, 4 independent immunoblotting experiments of at least 3 samples, PLSD Fisher test $p < 0.05$). Furthermore, as requested by the reviewer (see point 3 addressed in Fig 8C, 8D and 8E), we also demonstrated that Trastuzumab blocks downstream cellular events (cell motility, invasion) induced by mutant huntingtin.

Referee #2

Comments on Novelty/Model System:

Using a variety of approaches, and two mouse models for breast cancer that have been crossed to a knock-in mouse model for Huntington's disease, the authors have provided a comprehensive analysis of the effect of mutant huntingtin expression on the progression of breast cancer tumourigenesis. Their finding that metastasis is enhanced is novel, and is not what most would have predicted based on past analyses of cancer incidence in Huntington's disease patients. This past work showed that cancer incidence was significantly decreased in Huntington's disease. If Sousa et al.'s work is taken at face value, their findings are very important for both physicians and those involved in understanding pathogenic mechanisms in Huntington's disease. The use of two in vivo, and various in vitro models (e.g. matrigel cultures and cell mobility assays) is appropriate for the study.

Other Remarks:

In this study by Sousa et al., the authors find that in 12 HD patients with breast cancer, progression of the cancer was enhanced. To explore the molecular mechanism(s) responsible for this observation, the authors investigated tumourigenesis in two mouse models for breast cancer that have been crossed with a knock-in mouse model for Huntington's disease. Similar to what was observed in the HD patients with breast cancer, tumourigenesis in the mice expressing mutant htt was accelerated and the epithelial-mesenchymal transition of the cancer cells, a phenotype that can contribute to lung metastasis in the mice, was enhanced. Additional studies in the mouse models provided evidence that the accelerated progression of the breast cancer was due, in part, to hyperactivation of the ErbB2/HER2 signalling pathway.
These studies represent the first attempt to understand how mutant huntingtin expression could influence cancer progression. The majority of studies investigating mutant huntingtin pathogenesis have focused on the central nervous system, despite the fact that huntingtin is expressed throughout the body. Sousa et al.'s results with regard to mutant huntingtin and cancer progression are in stark contrast with the results of a study by Sorensen et al., 1999, and a more recent study by Ji et al., 2012 that suggested that the overall incidence of cancer was lower in Huntington's disease patients, and in patients with other expanded polyglutamine disorders. Although the mechanism(s) responsible for these findings was never explored further, the authors suggested that the effect of mutant huntingtin on cancer risk could be due to its apoptosis-promoting activity, and its ability to up regulate p53 expression. Interesting, Sousa et al. find an inverse correlation between the length of the expanded polyglutamine stretch and the age of breast cancer onset. A similar analysis in the Ji et al. study was not performed because CAG repeat length was not available for all patients, and instead, age-at-onset of Huntington's disease symptoms was used as a surrogate. When this parameter was used, the authors could find no correlation between HD age-at-onset and cancer incidence.
Nevertheless, Sousa et al. demonstrate convincingly that the observed alterations in HER2 signalling caused by mutant huntingtin expression can affect both tumour cell mobility and metastasis. These findings are important for the field, as they can inform physicians about the necessity for follow-up in Huntington's disease patients with cancers, and they also provide new clues about deficits in signalling pathways, and potential developmental alterations (due to changes in the EMT) that could occur in individuals at risk for Huntington's disease. There are, however, a few minor concerns, which if addressed, could improve the manuscript.

1. In the discussion, the authors state that their results neither confirm nor invalidate the Sorensen et al., 1999 study. This is a fair statement, but the authors should explore a comparison of their results with Sorensen et al. further in the discussion, as this issue will likely interest many readers. In addition, the authors should cite the recent Ji et al. study (The Lancet, published online April 12, 2012, DOI:10.1016/S1470-2045(12)70132-8) in their manuscript. Interestingly, Ji et al. find that the incidence of benign tumours is also decreased in Huntington's disease patients. If taken at face value, this result would suggest that differences in metastasis likely do not contribute to a reduced incidence of tumourigenesis in Huntington's disease patients, a conclusion that would be compatible with Sousa et al.'s study, and is also compatible with the idea that although cancer incidence may be reduced, progression may be enhanced in some cancers.
We particularly apologize for not citing the study of (Ji et al, 2012). At the time of the initial submission (May), we missed this publication and had access to it only during the review process.
We are now discussing this study (together with the study by (Sorensen et al, 1999)) p. 20 of the revised manuscript. Given the small size of our human study, we can not make definite statements. However, while the overall incidence of cancer is lower in HD patients, we can propose from our data that cancer progression may be enhanced in HD. Mutant huntingtin could have opposite effects on tumour appearance and EMT. This could be correlated with the CAG expansion. Finally, the increased severity may apply to some cancers only: for instance, this could be the case of breast cancer initiated by a HER2 accumulation.
For the referee interest: we have started a clinical study aiming at systematically investigating cancer in HD patients, in particular breast and ovarian cancer. The major goal of this study is not to analyze the incidence (this was examined in the above cited studies), rather we propose to examine whether the type and severity of cancer developed by HD patients are modified compared to the non HD population. We will have data on the size of the CAG expansion and will investigate whether there is a relation between the expansion size and the risk/severity of breast cancer.

2. To confirm their gene microarray studies, the authors assessed the mRNA and protein levels for a subset of genes, including Hdh. The authors state that mutant huntingtin mRNA was lower than that for wild-type huntingtin mRNA, but it is difficult to find this data in Fig. S2. It would help the reader to show this in a plot, especially because it is not clear that the western data for mutant huntingtin levels can be taken at face value. The authors apparently used the 4C8 antibody whose epitope is huntingtin amino acids 443-457 (Cong et al., 2005, Hybridoma, 24(5):231-5.). Do the authors know if this antibody recognizes huntingtin phosphorylated at S421 or acetylated at K444 equally as well as un-modified huntingtin? If not, the signal obtained with this antibody may not represent all the huntingtin that is present. Other antibodies that are insensitive to post-translational modifications could be used (e.g. PW0595, anti-N2-17 from ENZO). Alternatively, a recent study by Di Pardo et al., PNAS, 2012, 109(9):3528-3533 describes SDS-PAGE and western blot transfer conditions that increase substantially the amount of mutant huntingtin that can be detected in protein extracts.

We agree with the referee that the 4C8 can behave as a conformational antibody and in fact, in our hands using the 4C8 antibody the amount of mutant huntingtin may vary from 10 to 50% of the wild-type huntingtin depending on the experiments. As suggested by the reviewer, this may be linked not only to the actual level of the mutant protein but also to its phosphorylation or acetylation status. See one representative example in Figure 1 below.

We have performed new immunoblotting (Fig S3A) with a more C-terminal antibody, the D7F7 antibody (the N-terminal antibody cited by the referee does not always give consistent results in our hands). The D7F7 is a monoclonal antibody produced by immunizing rabbits with a synthetic peptide corresponding to residues surrounding proline 1220 of human huntingtin protein. This antibody was not found to vary depending on the phosphorylation status of huntingtin at S421 and S1181/1201 (not shown). D7F7 reveals a decrease of approximately 40% of huntingtin levels in the mutant situation as compared to the wild-type situation.

Furthermore, we have confirmed by quantitative real-time RT–PCR that the levels of huntingtin transcripts are decreased in MMTV-PyVT/$Hdh^{Q111/Q111}$ breast tumours as compared to the wild-type tumours (Fig 3E).

Interestingly, the D7F7 (relative level of wild-type huntingtin=1; polyQ-huntingtin=0.6), the microarrays (relative level of wild-type huntingtin=1; polyQ-huntingtin=0.64) and the RT-PCR data (relative level of wild-type huntingtin=1; polyQ-huntingtin=0.58) are very consistent.

Thus, in the revised version of the manuscript, we are now showing the results obtained with the D7F7 antibody for immunoblotting. Please note that in Fig 7E, we used the 4C8 antibody as it works very well for immunoprecipitation. It may still recognize less polyQ-huntingtin than wild-type huntingtin, however as it still did immunoprecipitate more dynamin in mutant conditions, this does not interfere with our conclusion.

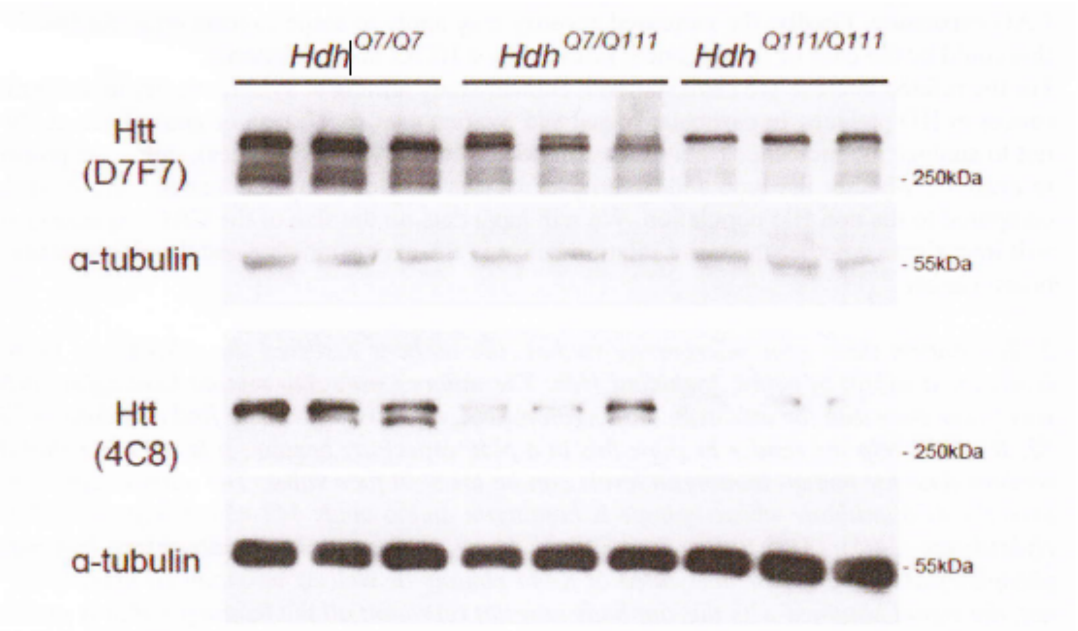

Figure 1 Total extracts from MMTV-PyVT/*Hdh*$^{Q7/Q7}$ (*Hdh*$^{Q7/Q7}$), MMTV-PyVT/*Hdh*$^{Q7/Q111}$ (*Hdh*$^{Q7/Q111}$) and MMTV-PyVT/*Hdh*$^{Q111/Q111}$ (*Hdh*$^{Q111/Q111}$) dissociated tumors. Immunoblotting for huntingtin (D7F7 and 4C8) and α-tubulin.

Referee #3

Comments on Novelty/Model System:

This paper is interesting and important, if the comments are well responded. Model systems used were adequate.

Other Remarks:

In this study, the authors tested the effect of huntingtin gene mutation on the progression of breast cancer. First, it was suggested that breast cancer phenotypes are worse in HD patients compared to non-HD population. Then, the authors examined the genetic interaction between huntingtin mutation and two different models of breast cancer and found that homozygotes for huntingtin mutation were associated with more aggressive phenotypes of mammary tumours. Accumulation of ErbB2 in mutant huntingtin-containing tumours was proposed as a potential mechanism for the aggressiveness.
Overall, this study includes some interesting observations related to peripheral effects of the huntingtin mutation. Even though the mechanism remains largely unclear, the results of model animal experiments seem to show the genetic effect of huntingtin mutations on the aggressiveness of breast tumours. However, the reviewer has some concerns on the manuscript.
Major points:
***1.** Even though two aspects of huntingtin mutation, loss of normal function and gain of toxic function, are emphasized throughout the text, it is still unclear whether the accelerated phenotypes in HdhQ111/Q111 tumours are explained by gain or loss of function of huntingtin (or both). Whereas knock-in animals are accurate genetic models of HD, it is difficult to separate the effects of gain and loss of function in these models, especially when comparing wild type and mutant homozygotes. In the text, the authors seem to presume the gain of function mechanism, which has not been directly examined. Only the experiments in Fig. 6C partially addressed this question, though the output was HER2 internalization but not the aggressive phenotypes of cancer cells. The reviewer requests the authors to compare the cellular phenotypes with or without RNAi-mediated knockdown of huntingtin in both HdhQ7/Q7 and HdhQ111/Q111 tumours.*
As requested, we performed knock-down of huntingtin in wild-type and mutant tumour cells and compared the motility behaviour in transwell assays, ErbB2 accumulation and Akt activation. The results are shown in Fig 2 below.
Removing wild-type huntingtin leads to an increased cell motility that resembles the polyQ situation. Removing mutant huntingtin from mutant cells is similar to the mutant situation and to the loss of huntingtin in wild-type cells. In parallel, ErbB2 levels and Akt activation are increased in wild-type cells with huntingtin knock-down, as well as in both mutant conditions. In the mutant situation, while the effect on migration is not statistically different from the wild-type cells with huntingtin knock-down, the effect on ErbB2 and Akt seems to be stronger. There may be several explanations for this. First, one should keep in mind that, while these different situations lead to the same phenotype, the molecular mechanisms are certainly different leading to different downstream responses (removing huntingtin is obviously not exactly identical to the addition of the mutant protein). Second, the knock-downs in both the wild-type and mutant situations are not complete, thus there is either a remaining amount of wild-type huntingtin (that has a full function) or of mutant huntingtin (that is not able to perform as the wild-type, by a gain of function mechanism).
In conclusion, removing huntingtin or expressing mutant huntingtin leads to a similar phenotype: in this case, we lose the function of huntingtin as a brake of motility. However, the molecular mechanisms may be slightly different: in mutant situation, polyQ-huntingtin interferes with dynamin as the dynamin/huntingtin interaction is enhanced so polyQ huntingtin acts as a dominant-negative form (you lose a function by a gain of function - the stronger interaction of huntingtin/dynamin). In the loss of function situation, huntingtin is absent. If huntingtin acts as a facilitator of dynamin function (at this stage this is a speculation), then dynamin activity would also be reduced (but probably to a different extent).

Thus, we certainly think that this topic is of great interest, not only in the case of this study but more generally for the study of the functions of huntingtin and for the HD field. In fact, we are currently focusing on loss of function/gain of function in motility/metastasis in the laboratory as it needs more work to be completely deciphered. Thus, in the current form of the manuscript we do not emphasize the loss/gain of function aspect. Indeed, we found it difficult to integrate to the current study. We have the impression that it is out of the scope and would be confusing. However, upon request of the referee, we could add the data and discuss this point.

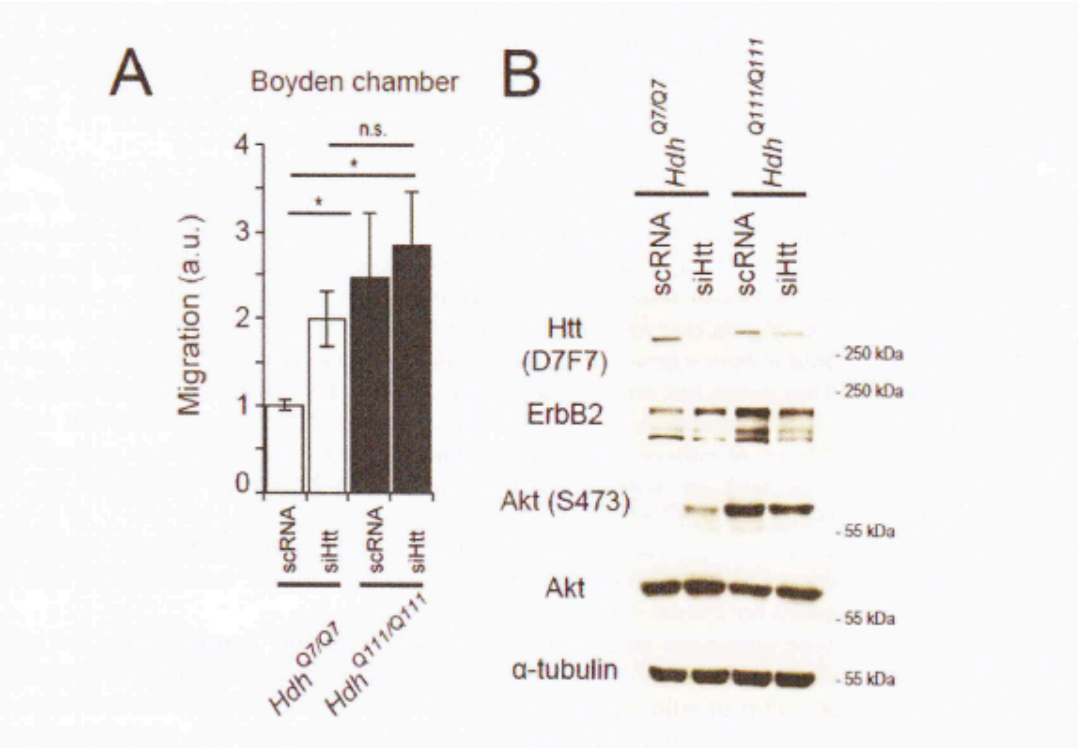

Figure 2 Knock-down of huntingtin in cells from MMTV-PyVT/*Hdh*$^{Q7/Q7}$ (*Hdh*$^{Q7/Q7}$) and MMTV-PyVT/*Hdh*$^{Q111/Q111}$ (*Hdh*$^{Q111/Q111}$) dissociated tumors.
A, Boyden chambers assays. **B**, Immunoblotting for huntingtin, ErbB2, phosphorylated Akt at serine 473 (Akt(S473)), total Akt and α-tubulin.
Statistical analysis: Data are from three independent experiments, 2 independent cell lines per genotype. *Hdh*$^{Q7/Q7}$ vs *Hdh*$^{Q7/Q7}$+siHtt, ANOVA F[11] = 14.893, T test p-value = 0.0027; *Hdh*$^{Q7/Q7}$ vs *Hdh*$^{Q111/Q111}$, ANOVA F[12] = 5.189, T test p-value = 0.0418; *Hdh*$^{Q7/Q7}$ vs *Hdh*$^{Q111/Q111}$+siHtt, ANOVA F[12] = 11.867, T test p-value = 0.049; *Hdh*$^{Q7/Q7}$+siHtt vs *Hdh*$^{Q111/Q111}$, ANOVA F[9] = 0.283, T test p-value = 0.6074; *Hdh*$^{Q7/Q7}$+siHtt vs *Hdh*$^{Q111/Q111}$+siHtt, ANOVA F[9] = 1.280, T test p-value = 0.2871; *Hdh*$^{Q111/Q111}$ vs *Hdh*$^{Q111/Q111}$+siHtt, ANOVA F[10] = 0.150, T test p-value = 0.7069.

***2.** While the authors propose ErbB2/HER2 elevation as a mechanism of exacerbation of breast cancer from the animal study, only 3 out of 6 examined HD patients have been confirmed to show the HER2+ phenotype (Fig. 1A). Given the small number of sample size, it is difficult to conclude at this moment that breast cancer in human HD is exacerbated though a HER2-related pathway. One concern is that this pathway is only relevant to the tumours of the HdhQ111/Q111 mice. At least, it should be tested whether the heterozygous HdhQ7/Q111 tumours show elevated ErbB2 compared to HdhQ7/Q7 mice.*
Indeed, future studies will examine whether breast cancer in HD is exacerbated through a HER2-related pathway. Other pathways could also be involved (see page p. 20).
In any case, as requested by the reviewer, we show that ErbB2 accumulates in breast tumours of heterozygous mice (Fig S3). The accumulation is intermediate compared to the wild-type and homozygous situations.

Minor points
1. How many HD cases were examined to find the patients with breast cancer? The incidence is high?
The patients with breast cancer were found among the patients followed-up at La Pitié-Salpêtrière Hospital/Department of Genetics by Dr. A. Durr. We can not provide a precise number of HD cases that were examined to find them. From this set of data, we can not draw conclusions about the incidence of breast cancer in the HD population.
In fact, we have started a clinical study aiming at systematically investigating cancer in HD patients, in particular breast and ovarian cancer. However, the major goal of this study is not to define the incidence (this was examined in the studies of (Sorensen et al, 1999) and (Ji et al, 2012)), rather we propose to examine whether the type and severity of cancer developed by HD patients are modified compared to the non HD population.

2. Fig 1B: The authors used rank order for both axes. If they use exact numbers of the repeat and age, is there significant correlation?
As suggested, we have changed panel B of Figure 1. Using the numbers of repeat and age, the correlation is significant: Pearson correlation coefficient= -0.58, p-value = 0.04.

3. Fig.1C. the reviewer agrees that this staining pattern is suggestive of the expression of huntingtin probably including mutant one. However, 4C8 antibody recognizes both wild type and mutant huntingtin and it is difficult to distinguish their expression patterns. It is suggested to modify the sentence (page 7, line 1-2), or examine the sections with antibody that specifically recognizes mutant, but not normal, huntingtin.
Indeed, the 4C8 antibody recognizes both the wild-type and mutant form of huntingtin. As suggested, we now provide immunohistochemical stainings using the 1C2 antibody that recognizes mutant but not wild-type huntingtin (Fig 1C). In particular, we observed that in the samples analysed, mutant huntingtin is enriched in the nucleus.
We conclude that mutant huntingtin is expressed in breast tumours. This warrants the interest of studying its influence during breast cancer progression.

4. The authors found no obvious increase in ErbB2 mRNA levels in wt and polyQ-htt mammary tumours. However, microarray data is not sufficient to exclude the effect of transcriptional level. qRT-PCR should be done for confirming this point.
We have confirmed that the levels of ErbB2 transcripts are similar in MMTV-PyVT/$Hdh^{Q7/Q7}$ and MMTV-PyVT/$Hdh^{Q111/Q111}$ breast tumours by quantitative RT–PCR (Fig 6C).

5. Microarray analysis. Were the p-values corrected for multiple comparisons or not? In either case, this point should be mentioned in the Materials and Methods.
The p-values were not corrected for multiple comparisons. This is now mentioned in the Materials and Methods (p. 27).

6. In figure 6D, Immunofluorescent data of HER2, E-cad, beta-catenin in Trastuzumab-treated cells should be added.
We have performed the required immunolabellings. In Fig 8B, in agreement with our data, immunostaining of ErbB2 revealed a decrease of cell surface labelling in PyVT/$Hdh^{Q111/Q111}$ primary tumor cells upon Trastuzumab treatment. We also performed E-cadherin and β-catenin labelling. However, given the size of Fig 8, we decided not to include these images. Furthermore, the stainings are not much informative. Indeed, in the experimental conditions used, given the toxicity of Trastuzumab, cells are cultured at confluence therefore reducing the effect of Trastuzumab on cell junction proteins (see Fig 3 below).

7. In figure 6F, why there is no increase even in HdhQ7/Q7 cells? Does Trastuzumab suppress cell growth of these cells?
To address this point, we have modified Figure 6F (now Fig 8F) and performed a more precise experiment: cells are treated for 24, 48 and 72 h with Trastuzumab, at each time points cells are counted. Trastuzumab does not affect cell growth in PyVT/$Hdh^{Q7/Q7}$ cells whereas it has a striking effect on PyVT/$Hdh^{Q111/Q111}$ cells.

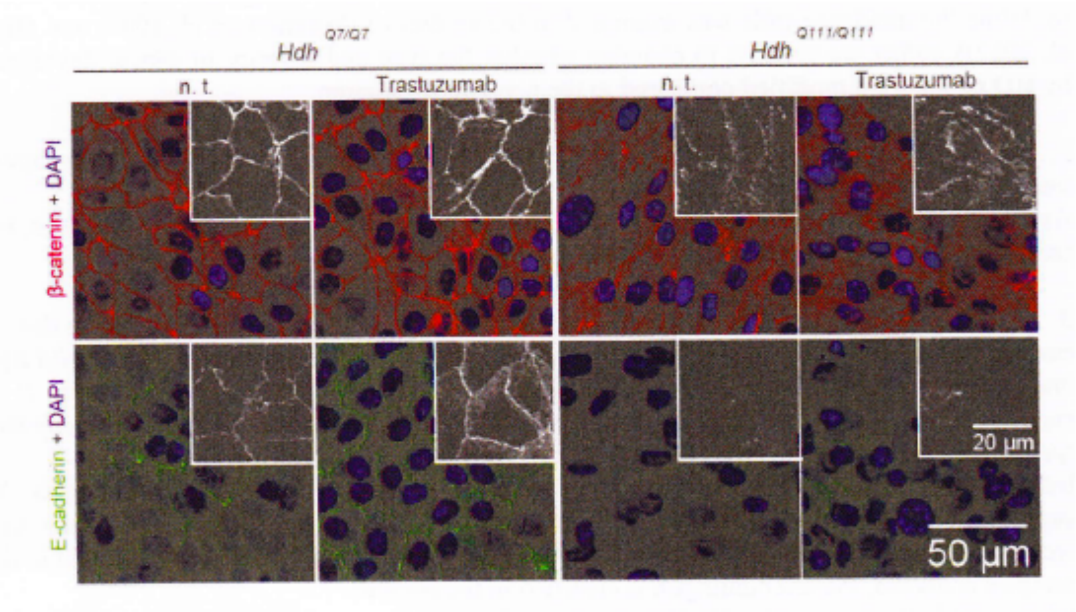

Figure 3 PyVT/*Hdh*$^{Q7/Q7}$ and PyVT/*Hdh*$^{Q111/Q111}$ cells treated with Trastuzumab are immunostained for endogenous β-catenin and E-cadherin as indicated. n.t.: not transfected.

References

Cheng WY, Kandel JJ, Yamashiro DJ, Canoll P, Anastassiou D (2012) A multi-cancer mesenchymal transition gene expression signature is associated with prolonged time to recurrence in glioblastoma. PLoS One 7: e34705

Ji J, Sundquist K, Sundquist J (2012) Cancer incidence in patients with polyglutamine diseases: a population-based study in Sweden. Lancet Oncol 13: 642-648

Kaltenbach LS et al (2007) Huntingtin interacting proteins are genetic modifiers of neurodegeneration. PLoS Genet 3: e82

Kim H, Watkinson J, Varadan V, Anastassiou D (2010) Multi-cancer computational analysis reveals invasion-associated variant of desmoplastic reaction involving INHBA, THBS2 and COL11A1. BMC Med Genomics 3: 51

Pedersen NM, Madshus IH, Haslekas C, Stang E (2008) Geldanamycin-induced down-regulation of ErbB2 from the plasma membrane is clathrin dependent but proteasomal activity independent. Mol Cancer Res 6: 491-500

Sorensen SA, Fenger K, Olsen JH (1999) Significantly lower incidence of cancer among patients with Huntington disease: An apoptotic effect of an expanded polyglutamine tract? Cancer 86: 1342-1346

Wheeler VC et al (1999) Length-dependent gametic CAG repeat instability in the Huntington's disease knock-in mouse. Hum Mol Genet 8: 115-122

Wolff AC et al (2007) American Society of Clinical Oncology/College of American Pathologists guideline recommendations for human epidermal growth factor receptor 2 testing in breast cancer. J Clin Oncol 25: 118-145

2nd Editorial Decision 25 October 2012

Thank you for the submission of your revised manuscript to our editorial offices. We have now received feedback from the two reviewers whom we asked to evaluate your manuscript.

As you will see from the enclosed reports, the Reviewers appreciate that you have performed additional experimentation. Reviewer 1 however, whose expertise lies in HER signalling and breast cancer, feels that major issues remain insufficiently addressed. The Reviewer remains of the opinion that 1) the sample size of the human study remains too small to draw firm conclusions with regard to the involvement of polyQ Htt in human breast cancer and 2) the biochemical evidence for the impact of Htt on HER signalling is based on small effects.

Given this lack of support, especially with respect to the evidence for involvement in human breast cancer, I have no choice but to return the manuscript to you at this stage.

I am very sorry to have to disappoint you on this occasion and sincerely hope that the Reviewer's comments are helpful in your continued work in this area.

Yours sincerely,

Editor
EMBO Molecular Medicine

***** Reviewer's comments *****

Referee #1 (Comments on Novelty/Model System):

This is the revision of a paper I have seen before and did not at that time think worthy of publication.
The work done since that time is of note.

However the paper still lacks unambiguous chemistry . The sample size remains too small.

Referee #1 (Remarks):

I think the authors did a considerable amount of work trying to improve the manuscript. Notably, the biochemistry data has been added to show the effect on signalling and internalization of HER2.

However, a number of major issues remain unsolved.

The small sample size of the human study makes it difficult to interpret the results. Despite the revision, there is little evidence that the polyQ htt protein is involved in human breast cancer. This is largely due the finding of polyQ htt proteins in both normal tissues and tumors. The finding that the polyQ htt protein is enriched in the nuclei is interesting. However, this appears to be found in just one case and the localization pattern in mouse cells is clearly different.

The biochemistry data generally show very modest differences. For example there is very little changes in signalling or HER2 cell surface levels. The evidence to support the proposed mechanism remains weak.

Referee #3 (Remarks):

The authors responded almost well to this reviewer's concern in the revised manuscript.

Appeal Received 26 October 2012

I do not understand your decision regarding our manuscript considering your last correspondence and the two main arguments leading to this decision. I therefore wish to make an appeal of your decision.

One point I appreciate about submitting to Embo Press, is to be able to discuss with editors and have helpful feedbacks during the review process.
For this reason, when we got the decision allowing us to revise the manuscript, I discussed with the editor about the points we were going to address in our revised manuscript (please find below our correspondence). The fact that the human sample size was too small has never been an issue for the putative publication of our study in Embo Molecular Medicine and was not requested by reviewer 1 at this stage. If this had been the case, I would not have resubmitted our study to Embo Molecular Medicine as the time frame allowed to revise a manuscript is obviously too short to perform a clinical study. Also, I anticipate that the editor would not have invited us to submit a revised version. In addition, as we discussed previously in the revised manuscript, the size of the sampling is not an argument -contrary to the claim of referee 1- of whether mutant polyQ-huntingtin is or not involved in human breast cancer. In fact, previous epidemiological studies suggest a link between HD and cancer (Ji et al, 2012; Sorensen et al, 1999). Our study demonstrates here both in cellular and mouse models, that HD mutation enhances aggressiveness of breast cancer.

The main point raised in the first round of the review was the weakness of the mechanistic insight. We have completely modified the manuscript and we provide information on how mutant huntingtin affects HER2/ErbB2 levels by a dynamin-dependent pathway. These are new findings that are acknowledged by referee 1 her/himself (and also by referee 3). The argument that the effects are small is just not admissible. Our effects are reproducible and statistically significant in a set of completely independent and complementary approaches (immunohistochemistry, immunoblotting, immunoprecipitation, cell immunostaining, FACS analysis). Furthermore, in experimental biology, "small effects" is not a scientific argument. We show that the dynamin-dependent endocytosis of the ErbB2/HER2 receptor tyrosine kinase is reduced in HD leading to HER2 accumulation. For instance, huntingtin interacts with dynamin with an altered binding when mutated, this is not a small effect: it is a fact!

I understand that reviewer 1 is an expert in the field of HER2, however despite his/her expertise, her/his last arguments (included in your last mail) are clearly showing that he/she is missing some important pathophysiological aspects of the study that are highly relevant for Huntingtonπs disease, a yet incurable devastating inherited neurodegenerative disease. In particular, her/his statement "Despite the revision, there is little evidence that the polyQ htt protein is involved in human breast cancer. This is largely due the finding of polyQ htt proteins in both normal tissues and tumors" is scientifically wrong (as were some of the statements in her/his first review). Why would the fact that mutant huntingtin is expressed in both the tumoral and non-affected tissue precludes its importance in cancer? What we are saying is that when mutant huntingtin is expressed, tumors are more agressive. Here we are investigating breast cancer in an HD genetic context. Importantly and as you may know, HD is due to a somatic mutation. This is completely different from genetic amplification of HER2 in breast tumor as the referee may be used to in her/his field.

I would also like to underline, that given the tone of reviewer 1, it was quite clear from the beginning that he/she would never be enthusiastic about our work. From the correspondence I had with the editor, it was obvious that the editor was aware of this when he/she allowed us to revise our manuscript.

Furthermore, we are surprised as we also addressed the points raised by reviewer 2 that the revised manuscript was not reviewed by him/her.

Although it may be difficult, I would really like to have the editor's opinion about this. I understand what can be the policy for journals in terms of reconsidering their decision. I also take in consideration that you and your colleagues work as a team of editors and support each other decision. However, the case of our manuscript is particular: we are in the context of a revision,

© EMBO

changing editor in the middle of the process is not trivial as upon the first round of the review the decision to consider a revised version of the manuscript despite referee 1 comments was already an editorial issue. I would thus really like you to reconsider your decision in view of the above mentioned arguments.

I would appreciate to discuss these points with you at your convenience. In particular, I can address your two main comments in more details (size of the sampling and "small" effects).

I thank you in advance for your time and consideration.

3rd Editorial Decision 08 November 2012

Thank you for messages asking us to reconsider our recent decision on your manuscript entitled "The Huntington disease protein accelerates breast tumor development and metastasis through ErbB2/HER2 signalling"

I have again discussed the points you raise with the Editorial team members including the Chief Editor. I also sought additional external advice from an expert Editorial Advisory Board Member. The external expert raises concerns similar to those of Reviewer 1: "the human clinical data remain weak, as the numbers are very small. Considering also that grade can be sometimes a little subjective, there is also the possibility of ascertainment bias".

While we agree with Reviewer 1 that the biochemistry on HER signalling and surface expression reveals small effects, we do take your point that small does not necessarily imply non functional. We are sympathetic with the opinion of our external Advisor who thinks that your work, notwithstanding the weakness of the human clinical data, is of interest and may stimulate further work at the interface between HD and cancer.

As a consequence and all considered, we feel that we can reconsider our decision. I thus invite you to submit a revised version in which the human clinical aspect de-emphasised, Fig.1 moved to the supplementary data and the discussion of this data is accompanied by appropriate cautions about its significance.

I hope that this solution is agreeable and very much look forward to receiving your revised version.

Finally, I wish to restate that all editorial decisions are discussed collegially to ensure fair and informed feedback to the Authors. Please consider, however, that no matter how supportive the Editors' might be of a specific manuscript (as in this case) the Reviewers' comments must be appropriately considered in the decisions.

*** PLEASE NOTE *** As part of the EMBO Publications transparent editorial process initiative (see our Editorial at http://onlinelibrary.wiley.com/doi/10.1002/emmm.201000094/full), EMBO Molecular Medicine will publish online a Review Process File to accompany accepted manuscripts. When preparing your letter of response, please be aware that in the event of acceptance, your cover letter/point-by-point document will be included as part of this file, which will be available to the scientific community. More information about this initiative is available in our Instructions to Authors. If you have any questions about this initiative, please contact the editorial office contact@embomolmed.org .

Yours sincerely,

Editor
EMBO Molecular Medicine

Additional Author Correspondence 12 November 2012

Thank you for your mail and for reconsidering your decision.

Indeed, I think that re-writing the human data with even more cautions about its significance is a good option. However, before sending a revised version, I would like to have your opinion. As you suggested I will move the Figure 1 in the supplemental data. However, I would like to propose to keep the IHC 1C and 1D as a new Figure 1 to show that mutant huntingtin is expressed in breast tumors. To my knowledge there is no data available showing the expression of mutant huntingtin in breast tumors. Thus, this would warrant the interest of studying its influence during breast cancer progression in mouse.

I am rewriting the text accompanying the human observations (removing the statements about the grade and molecular classification).

Let me know what you think.

Additional Editorial Correspondence 13 November 2012

I understand your point of view, but I would advise against this option. We invited you to submit a revision of your manuscript in which you tone down the clinical aspect, move Fig.1 to the supplementary section and caution about the significance of the data. To extract panels from former Fig.1 (which was criticised by both the Reviewer and the Advisory board Member) to form a new Fig.1 would sidestep the spirit of our request.

Again, supplementary figures are also important and so as a matter of fact, you are not being asked to drop data from your original revised submission.

I hope this is agreeable?

Yours sincerely,

Editor
EMBO Molecular Medicine

2nd Revision - authors' response 14 November 2012

We are submitting a revised version of our manuscript "The Huntington disease protein accelerates breast tumor development and metastasis through ErbB2/HER2 signalling".

In the revised version, we moved Figure 1 to the supplementary data. We have also modified the text to de-emphasize the human clinical data (see pages 2, 6 and 20 of the revised version).

We believe our study will be of interest to the reader of EMBO Molecular Medicine Journal not only as it may have direct implications for the follow-up and care of HD patients, but also as it provides new information about deficits in cellular functioning and signalling than can occur in HD situation. Furthermore, as suggested by the editorial advisory board member, this work should stimulate further studies at the interface between HD and cancer.

We hope that our study will now be acceptable for publication in the EMBO Molecular Medicine Journal.

We thank you for your time and consideration.

© EMBO

4th Editorial Decision 16 November 2012

Thank you for the submission of your revised manuscript to EMBO Molecular Medicine. I am pleased to inform you that we will be able to accept your manuscript pending the following final amendments:

The description of all reported data that includes statistical testing must state the name of the statistical test used to generate error bars and P values, the number (n) of independent experiments underlying each data point (not replicate measures of one sample), and the actual P value for each test (not merely 'significant' or 'P < 0.05').

Also, please follow the instructions for submission of the revised form as described below where you have not done so before.

Please submit your revised manuscript as soon as possible and in any case within 2 weeks. I look forward to seeing a revised form of your manuscript.

Yours sincerely,

Editor
EMBO Molecular Medicine

Revision received 19 November 2012

We are submitting a revised version of our manuscript "The Huntington disease protein accelerates breast tumor development and metastasis through ErbB2/HER2 signalling".

In the revised version, we included the name of the statistical tests used to generate error bars and P values, the number (n) of independent experiments underlying each data point, and the p-value for each test (except for values below 0.0001 where <0.0001 is indicated). These informations may be found in the Materials and Methods and in the Figure Legends. We also followed the instructions for submission of a revised form as indicated in your last mail.

We hope that our study will now be acceptable for publication in the EMBO Molecular Medicine Journal.

We thank you for your time and consideration.
