# Supplementary material for: The Huntington disease protein accelerates breast tumour development and metastasis through ErbB2/HER2 signalling
Source: EMBO Mol Med. 2013 Jan 9;5(2):309–25. doi: 10.1002/emmm.201201546 (PMC3569645; doi:10.1002/emmm.201201546)
Supplement: Supplementary file 2 [file emmm0005-0309-SD2.pdf]

SUPPORTING INFORMATION, TABLE OF CONTENTS

**Experimental procedures**	**page 2**
**Statistical analysis**	**page 3**
**Legends**	**page 9**
**References**	**page 12**
**Table S1**	**page 13**
**Table S2**	**page 15**
**Table S3**	**page 17**
**Figure S1**	**page 18**
**Figure S2**	**page 19**
**Figure S3**	**page 20**
**Figure S4**	**page 21**

SUPPORTING INFORMATION

Experimental procedures

Histochemistry. Written informed consent for the use of human tissue specimens was given. Human samples were deparaffinised, demasked in Dako EDTA pH 9 antigen retrieval solution (90°C, 30 min) and labelled for the presence of huntingtin (4C8, 1:300; 1C2, 1:10000). Samples were probed using a Vectastain ABC kit (Vector laboratories) and counterstained with hematoxylin. Tumors originating from the fourth mammary glands or from lungs were dissected and fixed in 4% paraformaldehyde. Samples were imbedded in paraffin and the blocks were cut (7 μm) and mounted on glass slides. Slides were deparaffinised, demasked in citrate buffer 10mM pH 6.0 (90°C, 20 min), probed with antibodies against cleaved caspase-3 (1:200), PCNA (1:200), Ki67 (1:500) and PyVT (1:250) and revealed using the Vectastain ABC kit.

Gene Ontology analysis (GO). Biological process enrichment was performed using AmiGO platform version:1.8 (http://amigo.geneontology.org; Carbon S, et al. AmiGO: online access to ontology and annotation data. *Bioinformatics*. 2009; 25(2): 288-9), GO term enrichment function. Microarray regulated genes found with more than 1.5 fold difference and p-value>0.05 recognized by the platform were used as input. Thresholds were set at Max p-value: 0.05 and Min genes: 2. General biological processes (with more than 1,500 genes associated) were excluded from the final results.

Statistical analysis

Statview 4.5 software (SAS Institute, Cary, NC) was used for statistical analyses. Data are expressed as means ± SE.

Figure 1A Tumor free survival data are from paired littermates from twelve different litters. MMTV-PyVT/*Hdh*$^{Q7/Q7}$: 19 mice; MMTV-PyVT/*Hdh*$^{Q7/Q111}$: 37 mice; MMTV-PyVT/*Hdh*$^{Q111/Q111}$: 25 mice. Kaplan-Meier Analysis, Logrank test: p value < 0.0001 Chi-2 = 22.60.

Figure 1C Tumor progression data are from abdominal mammary glands dissected from independent groups of mice scarified at different ages.

At 6 weeks, MMTV-PyVT/*Hdh*$^{Q7/Q7}$: 4 glands, MMTV-PyVT/*Hdh*$^{Q7/Q111}$: 7 glands, MMTV-PyVT/*Hdh*$^{Q111/Q111}$: 3 glands. ANOVA F[11] = 1.500. PLSD Fisher test: MMTV-PyVT/*Hdh*$^{Q7/Q7}$ vs. MMTV-PyVT/*Hdh*$^{Q7/Q111}$: p-value = 0.1867; MMTV-PyVT/*Hdh*$^{Q7/Q7}$ vs. MMTV-PyVT/*Hdh*$^{Q111/Q111}$: p-value = 0.1390; MMTV-PyVT/*Hdh*$^{Q7/Q111}$ vs. MMTV-PyVT/Hdh$^{Q111/Q111}$: p-value = 0.6363.

At 8 weeks, MMTV-PyVT/*Hdh*$^{Q7/Q7}$: 3 glands, MMTV-PyVT/*Hdh*$^{Q7/Q111}$: 3 glands, MMTV-PyVT/*Hdh*$^{Q111/Q111}$: 4 glands. ANOVA F[7] = 3.440. PLSD Fisher test: MMTV-PyVT/*Hdh*$^{Q7/Q7}$ vs. MMTV-PyVT/*Hdh*$^{Q7/Q111}$: p-value = 0.7262; MMTV-PyVT/*Hdh*$^{Q7/Q7}$ vs. MMTV-PyVT/*Hdh*$^{Q111/Q111}$: p-value = 0.0482; MMTV-PyVT/*Hdh*$^{Q7/Q111}$ vs. MMTV-PyVT/Hdh$^{Q111/Q111}$: p-value = 0.0856.

At 12 weeks, MMTV-PyVT/*Hdh*$^{Q7/Q7}$: 4 glands, MMTV-PyVT/*Hdh*$^{Q7/Q111}$: 4 glands, MMTV-PyVT/*Hdh*$^{Q111/Q111}$: 5 glands. ANOVA F[10] = 6.826. PLSD Fisher test: MMTV-PyVT/*Hdh*$^{Q7/Q7}$ vs. MMTV-PyVT/*Hdh*$^{Q7/Q111}$: p-value = 0.1576; MMTV-PyVT/*Hdh*$^{Q7/Q7}$ vs. MMTV-PyVT/*Hdh*$^{Q111/Q111}$: p-value = 0.0044; MMTV-PyVT/*Hdh*$^{Q7/Q111}$ vs. MMTV-PyVT/Hdh$^{Q111/Q111}$: p-value = 0.0671.

At 14 weeks, MMTV-PyVT/*Hdh*$^{Q7/Q7}$: 4 glands, MMTV-PyVT/*Hdh*$^{Q7/Q111}$: 6 glands, MMTV-PyVT/*Hdh*$^{Q111/Q111}$: 4 glands. ANOVA F[11] = 7.912. PLSD Fisher test: MMTV-PyVT/*Hdh*$^{Q7/Q7}$ vs. MMTV-PyVT/*Hdh*$^{Q7/Q111}$: p-value = 0.0186; MMTV-PyVT/*Hdh*$^{Q7/Q7}$ vs. MMTV-PyVT/*Hdh*$^{Q111/Q111}$: p-value = 0.0024; MMTV-PyVT/*Hdh*$^{Q7/Q111}$ vs. MMTV-PyVT/Hdh$^{Q111/Q111}$: p-value = 0.1558.

Figure 1E Percentage of positive nuclei stained for cleaved caspase-3, MMTV-PyVT/*Hdh*$^{Q7/Q7}$: 3 tumors, 13178 cells scored, MMTV-PyVT/*Hdh*$^{Q111/Q111}$: 3 tumors, 16554 cells scored, ANOVA F[4] = 1.075; T test p-value = 0.3583; PCNA, MMTV-PyVT/*Hdh*$^{Q7/Q7}$: 3 tumors, 1858 cells scored, MMTV-PyVT/*Hdh*$^{Q111/Q111}$: 3 tumors, 2177 cells scored, ANOVA F[4] =72.675; T test p-value = 0.0010 and Ki67, MMTV-PyVT/*Hdh*$^{Q7/Q7}$: 3 tumors, 1224 cells scored, MMTV-PyVT/*Hdh*$^{Q111/Q111}$: 3 tumors, 1074 cells scored ANOVA F[4] = 24.005; T test p-value = 0.0080.

Figure 2E *Htt* mRNA levels were quantified from RNA isolated from 4 MMTV-PyVT/*Hdh*$^{Q7/Q7}$ and 3 MMTV-PyVT/*Hdh*$^{Q111/Q111}$ tumors. ANOVA F[5] = 12.542; T test p-value = 0.0165.

Figure 3B MMTV-PyVT/*Hdh*$^{Q7/Q7}$: 6 tumors; MMTV-PyVT/*Hdh*$^{Q111/Q111}$: 6 tumors. Two independent immunoblotting. ZO-1/tubulin ratio: ANOVA F[10] = 23.156, T test p-value = 0.0007. E-cadherin/tubulin ratio: ANOVA F[10] = 6.439, T test p-value = 0.0295. β-catenin/tubulin ratio, ANOVA F[10] = 7.094, T test p-value = 0.0238. Vimentin/tubulin ratio, ANOVA F[10] = 13.963, T test p-value = 0.0039.

Figure 4A Data are from three independent experiments. MMTV-PyVT/$Hdh^{Q7/Q7}$: 3 tumors, 110 cells; MMTV-PyVT/$Hdh^{Q111/Q111}$: 3 tumors, 149 cells. ANOVA F[257] = 143.384, T test p-value < 0.0001.

Figure 4B Data are from three independent experiments. MMTV-PyVT/$Hdh^{Q7/Q7}$: 6 tumors, 12 inserts; MMTV-PyVT/$Hdh^{Q111/Q111}$: 4 tumors, 8 inserts. ANOVA F[18] = 23.162, T test p-value < 0.0001.

Figure 4C Data are from four independent experiments. MMTV-PyVT/$Hdh^{Q7/Q7}$: 8 tumors, 16 inserts, MMTV-PyVT/$Hdh^{Q111/Q111}$: 7 tumors, 14 inserts. ANOVA F[28] = 25.589, T test p-value < 0.0001.

Figure 4D Data are from four independent experiments. MMTV-PyVT/$Hdh^{Q7/Q7}$: 4 tumors; MMTV-PyVT/$Hdh^{Q111/Q111}$: 5 tumors. Live cells, ANOVA F[12] = 9.370, T test p-value = 0.0099. Apoptotic cells, ANOVA F[12] = 3.562, T test p-value = 0.0835. Dead cells, ANOVA F[12] = 7.677, T test p-value = 0.0169.

Figure 4E Metastatic foci quantification on lungs from MMTV-PyVT/$Hdh^{Q7/Q7}$ and MMTV-PyVT/$Hdh^{Q111/Q111}$ mice at 12 weeks of age. MMTV-PyVT/$Hdh^{Q7/Q7}$: 5 lungs, MMTV-PyVT/$Hdh^{Q111/Q111}$: 5 lungs. ANOVA F[8] = 13.727, T test p-value = 0.0060.

Figure 4F Metastatic foci quantification on lungs from Swiss-nude mice grafted with MMTV-PyVT tumors, one experiment. MMTV-PyVT/$Hdh^{Q7/Q7}$: 5 mice, MMTV-PyVT/$Hdh^{Q111/Q111}$: 4 mice. ANOVA F[7] = 7.827, T test p-value = 0.0266. A second experiment gave similar results.

Figure 5B ErbB2/tubulin ratio, immunoblotting quantification. MMTV-PyVT/$Hdh^{Q7/Q7}$: 9 tumors; MMTV-PyVT/$Hdh^{Q111/Q111}$: 9 tumors. ANOVA F[16] = 10.168, T test p-value = 0.0057. Phospho-Akt/Akt ratio, immunoblotting quantification. MMTV-PyVT/$Hdh^{Q7/Q7}$: 5 tumors; MMTV-PyVT/$Hdh^{Q111/Q111}$: 5 tumors. ANOVA F[8] = 6.637, T test p-value = 0.0328.

Figure 5C *ErbB2* mRNA levels were quantified from RNA isolated from 4 MMTV-PyVT/$Hdh^{Q7/Q7}$ and 3 MMTV-PyVT/$Hdh^{Q111/Q111}$ independent tumors. ANOVA F[5] = 0.122; T test p-value = 0.7441.

Figure 6A Geldanamycin induced HER2 degradation quantification data are from three independent experiments. DMSO: 27 cells; Geldanamycin: 24 cells; pARIS-httQ23, DMSO: 33 cells; pARIS-Qhtt23, Geldanamycin: 27 cells; pARIS-httQ100, DMSO: 28 cells; pARIS-httQ100, Geldanamycin: 26 cells. ANOVA F[58] = 53.771. PSLD Fisher test: Q23 DMSO vs Q23 Geldanamycin p-value < 0.0001. Q100 DMSO vs Q100 Geldanamycin p-value < 0.0001. DMSO vs Geldanamycin p-value < 0.0001. Geldanamycin vs Q23 Geldanamycin p-value = 0.2377. Q23 Geldanamycin vs Q100 Geldanamycin p-value = 0.0006. Geldanamycin vs Q100 Geldanamycin p-value < 0.0001. Q23 DMSO vs Q100 DMSO p-value = 0.0204. Q23 DMSO vs DMSO p-value = 0.4335. Q100 DMSO vs DMSO p-value = 0.1502.

Figure 6B Geldanamycin induced HER2 degradation quantification data are from three independent experiments. 10,000 cells were analyzed per condition and experiment. ANOVA F[8] = 91.837. PSLD Fisher test: Q23 DMSO vs Q100 DMSO p-value = 0.0141. Q23 DMSO vs Q23 Geldanamycin p-value < 0.0001. Q23 DMSO vs Q100 Geldanamycin p-value = 0.0001. Q100 DMSO vs Q23 Geldanamycin p-value < 0.0001. Q100 DMSO vs Q100 Geldanamycin p-value < 0.0001. Q23 Geldanamycin vs Q100 Geldanamycin p-value = 0.0009.

Figure 6C Data are from three independent cytomety experiments. 10,000 cells were analyzed per condition and experiment. ANOVA F[12] = 33.463. PSLD Fisher test: DMSO vs Geldanamycin, p-value < 0.0001; DMSO vs Dyn2 K44A DMSO, p-value > 0.9999; DMSO vs Dyn2 K44A Geldanamycin, p-value = 0.0002; Geldanamycin vs Dyn2 K44A

DMSO p-value < 0.0001; Geldanamycin vs Dyn2 K44A Geldanamycin, p-value < 0.0001; Dyn2 K44A DMSO vs Dyn2 K44A Geldanamycin, p-value = 0.0002.

Figure 6D Data are from three independent cytomety experiments with 10,000 cells were analyzed per condition and experiment. ANOVA F[12] = 19.221. PSLD Fisher test: Q23 vs Q100, p-value = 0.0491; Q23 vs Q23+Dyn2 WT, p-value = 0.002; Q23 vs Q100+Dyn2 WT, p-value = 0.0522; Q100 vs Q23+Dyn2 WT, p-value < 0.0001; Q100 vs Q100+Dyn2 WT, p-value = 0.0016; Q23+Dyn2 WT vs Q100+Dyn2 WT, p-value = 0.0136.

Figure 7C Data are from three independent experiments. PyVT/*Hdh*$^{Q7/Q7}$: 3 tumors, 118 cells not treated, 118 cells treated with Trastuzumab; PyVT/*Hdh*$^{Q111/Q111}$: 3 tumors, 118 cells not treated, 118 cells treated with Trastuzumab. ANOVA F[468] = 27.042. PLSD Fisher test: PyVT/*Hdh*$^{Q7/Q7}$ vs PyVT/*Hdh*$^{Q111/Q111}$, p-value < 0.0001; PyVT/*Hdh*$^{Q7/Q7}$ Trastuzumab vs PyVT/*Hdh*$^{Q111/Q111}$, p-value < 0.0001; PyVT/*Hdh*$^{Q7/Q7}$ vs PyVT/*Hdh*$^{Q7/Q7}$ Trastuzumab, p-value = 0.6326; PyVT/*Hdh*$^{Q111/Q111}$ vs PyVT/*Hdh*$^{Q111/Q111}$ Trastuzumab, p-value < 0.0001.

Figure 7D Data are from three independent experiments, 3 tumors, 8 inserts per genotype and condition. ANOVA F[28] = 11.857. PLSD Fisher test: PyVT/*Hdh*$^{Q7/Q7}$ vs PyVT/*Hdh*$^{Q111/Q111}$, p-value = 0.0001; PyVT/*Hdh*$^{Q7/Q7}$ Trastuzumab vs PyVT/*Hdh*$^{Q111/Q111}$, p-value = 00795; PyVT/*Hdh*$^{Q7/Q7}$ vs PyVT/*Hdh*$^{Q7/Q7}$ Trastuzumab, p-value = 0.2682; PyVT/*Hdh*$^{Q111/Q111}$ vs PyVT/*Hdh*$^{Q111/Q111}$ Trastuzumab, p-value = 0.0007.

Figure 7E Data are from three independent experiments, 3 tumors, 8 inserts per genotype and condition. ANOVA F[14] = 8.286. PLSD Fisher test: PyVT/*Hdh*$^{Q7/Q7}$ vs PyVT/*Hdh*$^{Q111/Q111}$, p-value = 0.0012; PyVT/*Hdh*$^{Q7/Q7}$ Trastuzumab vs PyVT/*Hdh*$^{Q111/Q111}$, p-value = 0.1355; PyVT/*Hdh*$^{Q7/Q7}$ vs PyVT/*Hdh*$^{Q7/Q7}$ Trastuzumab, p-value = 0.7599; PyVT/*Hdh*$^{Q111/Q111}$ vs PyVT/*Hdh*$^{Q111/Q111}$ Trastuzumab, p-value = 0.0101.

Figure 7F Data are from three independent experiments with cells derived from three independent tumors of each genotype.

48h: ANOVA F[24] = 11.176. PLSD Fisher test: PyVT/$Hdh^{Q7/Q7}$ vs PyVT/$Hdh^{Q111/Q111}$, p-value = 0.2074. PyVT/$Hdh^{Q7/Q7}$ Trastuzumab vs PyVT/$Hdh^{Q111/Q111}$, p-value = 0.0023; PyVT/$Hdh^{Q7/Q7}$ vs PyVT/$Hdh^{Q7/Q7}$ Trastuzumab, p-value = 0.0445; PyVT/$Hdh^{Q111/Q111}$ vs PyVT/$Hdh^{Q111/Q111}$ Trastuzumab, p-value = 0.0356.

72h: ANOVA F[24] = 10.947. PLSD Fisher test: PyVT/$Hdh^{Q7/Q7}$ vs PyVT/$Hdh^{Q111/Q111}$, p-value = 0.8391; PyVT/$Hdh^{Q7/Q7}$ Trastuzumab vs PyVT/$Hdh^{Q111/Q111}$, p-value = 0.4605; PyVT/$Hdh^{Q7/Q7}$ vs PyVT/$Hdh^{Q7/Q7}$ Trastuzumab, p-value = 0.5909; PyVT/$Hdh^{Q111/Q111}$ vs PyVT/$Hdh^{Q111/Q111}$ Trastuzumab, p-value = 0.0002.

Supplementary figure 2A Tumor free survival data were analyzed by pooling the data from four different experiments. MMTV-ErbB2/$Hdh^{Q7/Q7}$: 25 mice, MMTV-ErbB2/$Hdh^{Q111/Q111}$: 9 mice. Kaplan-Meier Analysis, Logrank test: p value < 0.0001, Chi-2 =33.098.

Legends

Table S1 Genes of interest found dysregulated in MMTV-PyVT/*Hdh*$^{Q7/Q7}$ versus MMTV-PyVT/*Hdh*$^{Q111/Q111}$ mammary tumors (p-value < 0.05; 1.5 fold increase).

Table S2 Biological processes found enriched in MMTV-PyVT/*Hdh*$^{Q111/Q111}$ versus MMTV-PyVT/*Hdh*$^{Q7/Q7}$ mammary tumors (p-value < 0.05). GO analysis of microarray data.

Table S3 EMT associated genes found dysregulated in MMTV-PyVT/*Hdh*$^{Q7/Q7}$ versus MMTV-PyVT/*Hdh*$^{Q111/Q111}$ mammary tumors. The list of genes corresponds to a multi-cancer stage associated gene expression signature enriched in EMT markers (Cheng et al, 2012; Kim et al, 2010).

Figure S1. HD patients and breast cancer.

A. Twelve HD patients who developed breast cancer were identified. Their CAG number for the mutant allele, age of diagnosis for HD and breast cancer, breast cancer grade (EE, as described by (Elston et al, 1991)) and phenotype, and axillary lymph node status are listed. ER, estrogen receptor; PR, progesterone receptor; N0, node negative; N1, node invasion; n.a., not available; AS, asymptomatic.

B. Correlation between age at diagnosis of the breast cancer (BC) and CAG repeats.

C. Immunohistochemical staining of HD human breast tumor biopsies with the 4C8 antibody recognizing both the wild-type and the mutant form of huntingtin.

D. Immunohistochemical staining of human breast biopsies with the 1C2 mutant specific antibody. Several cells show a strong nuclear staining. The non HD sample is used as a negative control.

Figure S2. Mutant huntingtin increases tumorigenesis and metastasis in MMTV-ErbB2 mouse model.

A. Tumor-free survival curve of MMTV-ErbB2/*Hdh*$^{Q7/Q7}$ (*Hdh*$^{Q7/Q7}$*;* t_{50} = 167 ± 5 days; n = 25) and MMTV-ErbB2/*Hdh*$^{Q111/Q111}$ (*Hdh*$^{Q111/Q111}$; t_{50} = 79 ± 19 days; n = 9) mice. Kaplan-Meier Analysis, Logrank test: p value < 0.0001.

B. Whole mount carmine aluminum staining of MMTV-ErbB2/*Hdh*$^{Q7/Q7}$ and MMTV-ErbB2/*Hdh*$^{Q111/Q111}$ abdominal mammary glands at 24 weeks.

C. Western blot analysis of primary mammary tumors from MMTV-ErbB2/*Hdh*$^{Q7/Q7}$ and MMTV-ErbB2/*Hdh*$^{Q111/Q111}$ using antibodies against E-cadherin, β-catenin, vimentin and α-tubulin.

D. Hematoxylin and Eosin staining of lungs from nude mice grafted with MMTV-ErbB2/*Hdh*$^{Q7/Q7}$ and MMTV-ErbB2/*Hdh*$^{Q111/Q111}$ tumors.

Figure S3. Gene profile analysis using Affymetrix Mouse Exon 1.0 ST arrays on MMTV-PyVT/*Hdh*$^{Q7/Q7}$ versus MMTV-PyVT/*Hdh*$^{Q111/Q111}$ primary tumors.

A. Hierarchical clustering of regulated genes by gene intensity. WT: MMTV-PyVT/*Hdh*$^{Q7/Q7}$ tumors; polyQ: MMTV-PyVT/*Hdh*$^{Q111/Q111}$ tumors.

B. Western blot analysis of extracts from mammary tumors from MMTV-PyVT/*Hdh*$^{Q7/Q7}$ and MMTV-PyVT/*Hdh*$^{Q111/Q111}$ mice using antibodies against Cadherin 11, BARD-1, MMP3, cyclinD2 and α-tubulin as loading control.

Figure S4. ErbB2 accumulates in HD.

Western blot analysis of extracts from mammary tumors from MMTV-PyVT/*Hdh*$^{Q7/Q7}$, (*Hdh*$^{Q7/Q7}$), MMTV-PyVT/*Hdh*$^{Q7/Q111}$ (*Hdh*$^{Q7/Q111}$) and MMTV-PyVT/*Hdh*$^{Q111/Q111}$ (*Hdh*$^{Q111/Q111}$)

mice using antibodies against (**A**) huntingtin (D7F7), (**B**) ErbB2 and α-tubulin as loading control.

References

Cheng WY, Kandel JJ, Yamashiro DJ, Canoll P, Anastassiou D (2012) A multi-cancer mesenchymal transition gene expression signature is associated with prolonged time to recurrence in glioblastoma. PLoS One **7:** e34705

Elston CW, Ellis IO (1991) Pathological prognostic factors in breast cancer. I. The value of histological grade in breast cancer: experience from a large study with long-term follow-up. Histopathology **19:** 403-410

Kim H, Watkinson J, Varadan V, Anastassiou D (2010) Multi-cancer computational analysis reveals invasion-associated variant of desmoplastic reaction involving INHBA, THBS2 and COL11A1. BMC Med Genomics **3:** 51

Table S1

Gene ID	Gene Symbol	Reg	P-Value	Gene Name	ECM	Cytosk	Prolif/death
18606	**Enpp2**	2.51	3.16E-03	ectonucleotide pyrophosphatase/phosphodiesterase 2		+	
17392	**Mmp3**	2.47	1.58E-02	matrix metallopeptidase 3	+		
20307	**Ccl8**	2.39	2.86E-02	chemokine (C-C motif) ligand 8	+		
12825	**Col3a1**	2.24	1.07E-02	collagen, type III, alpha 1	+		
76477	**Pcolce2**	2.13	1.02E-03	procollagen C-endopeptidase enhancer 2	+		
13179	**Dcn**	2.11	1.07E-02	decorin	+	+	
56213	**Htra1**	2.09	5.97E-04	HtrA serine peptidase 1			+
216616	**Efemp1**	2.09	4.92E-02	epidermal growth factor-containing fibulin-like extracellular matrix protein 1	+		+
14314	**Fstl1**	2.05	1.12E-03	follistatin-like 1	+	+	+
71712	**Dram1**	2.05	3.58E-03	DNA-damage regulated autophagy modulator 1			+
21961	**Tns1**	2.02	1.19E-02	tensin 1	+	+	
56318	**Acpp**	2.02	4.21E-02	acid phosphatase, prostate			+
72169	**Trim29**	2.02	1.59E-02	tripartite motif-containing 29			+
13479	**Dpep1**	2.01	4.08E-03	dipeptidase 1 (renal)	+	+	
12833	**Col6a1**	1.99	9.88E-03	collagen, type VI, alpha 1	+		
208936	**Adamts18**	1.99	2.67E-02	a disintegrin-like and metallopeptidase (reprolysin type) with thrombospondin type 1 motif, 18	+		+
18113	**Nnmt**	1.95	1.75E-02	nicotinamide N-methyltransferase	+		
22061	**Trp63**	1.95	2.42E-02	transformation related protein 63			+
17022	**Lum**	1.91	2.73E-02	lumican		+	
11727 // 58809	**Ang // Rnase4**	1.88	2.46E-03	angiogenin, ribonuclease, RNase A family, 5 // ribonuclease, RNase A family 4	+		+
116847	**Prelp**	1.82	9.08E-03	proline arginine-rich end leucine-rich repeat	+	+	
18595	**Pdgfra**	1.81	2.08E-03	platelet derived growth factor receptor, alpha polypeptide	+	+	+
11475	**Acta2**	1.80	4.52E-02	actin, alpha 2, smooth muscle, aorta		+	
12444	**Ccnd2**	1.76	1.21E-02	cyclin D2			+
227753	**Gsn**	1.75	4.24E-02	gelsolin		+	+
216725	**Adamts2**	1.73	4.02E-03	a disintegrin-like and metallopeptidase (reprolysin type) with thrombospondin type 1 motif, 2	+		+
21916	**Tmod1**	1.73	4.35E-02	tropomodulin 1		+	
58223	**Mmp19**	1.72	3.35E-02	matrix metallopeptidase 19	+		
16000	**Igf1**	1.71	2.74E-02	insulin-like growth factor 1	+		+
81877	**Tnxb**	1.69	4.74E-02	tenascin XB	+		+
18073	**Nid1**	1.67	8.22E-03	nidogen 1	+		
16773	**Lama2**	1.67	1.24E-03	laminin, alpha 2	+	+	
93737	**Pard6g**	1.67	6.14E-03	par-6 partitioning defective 6 homolog gamma (C. elegans)		+	
20292	**Ccl11**	1.66	3.73E-02	chemokine (C-C motif) ligand 11	+	+	
17390	**Mmp2**	1.66	3.92E-03	matrix metallopeptidase 2	+	+	
17132	**Maf**	1.65	4.66E-03	avian musculoaponeurotic fibrosarcoma (v-maf) AS42 oncogene homolog			+
170643	**Kirrel**	1.64	3.98E-02	kin of IRRE like (Drosophila)		+	
16949	**Loxl1**	1.63	7.82E-03	lysyl oxidase-like 1	+		+
16971	**Lrp1**	1.62	1.34E-03	low density lipoprotein receptor-related protein 1		+	+
50530	**Mfap5**	1.62	3.87E-02	microfibrillar associated protein 5	+	+	
57266	**Cxcl14**	1.62	4.90E-02	chemokine (C-X-C motif) ligand 14	+	+	
12111	**Bgn**	1.61	1.02E-02	biglycan	+		
20692	**Sparc**	1.60	2.08E-02	secreted acidic cysteine rich glycoprotein	+		
12552	**Cdh11**	1.60	7.61E-04	cadherin 11		+	+

20315	**Cxcl12**	1.57	3.58E-02	chemokine (C-X-C motif) ligand 12	+	+	
18542	**Pcolce**	1.57	1.92E-02	procollagen C-endopeptidase enhancer protein	+		
23794	**Adamts5**	1.57	4.26E-02	a disintegrin-like and metallopeptidase (reprolysin type) with thrombospondin type 1 motif, 5	+		
14114	**Fbln1**	1.57	4.42E-03	fibulin 1	+	+	
241639	**Fermt1**	1.57	1.63E-02	fermitin family homolog 1 (Drosophila)	+	+	+
20564	**Slit3**	1.56	1.99E-02	slit homolog 3 (Drosophila)	+	+	
114893	**Dcun1d1**	1.54	5.70E-03	DCN1, defective in cullin neddylation 1, domain containing 1 (S. cerevisiae)	+		
20317	**Serpinf1**	1.53	4.34E-03	serine (or cysteine) peptidase inhibitor, clade F, member 1	+		+
13555	**E2f1**	1.53	3.95E-02	E2F transcription factor 1			+
219151	**Scara3**	1.53	2.16E-03	scavenger receptor class A, member 3			+
233744	**Spon1**	1.52	2.54E-03	spondin 1, (f-spondin) extracellular matrix protein	+		
12021	**Bard1**	1.51	6.02E-03	BRCA1 associated RING domain 1			+
13732	**Emp3**	1.51	2.27E-02	epithelial membrane protein 3			+
12834	**Col6a2**	1.50	1.20E-02	collagen, type VI, alpha 2	+		
71785	**Pdgfd**	-1.52	1.59E-02	platelet-derived growth factor, D polypeptide	+		+
58214	**Cst10**	-1.56	1.60E-03	cystatin 10 (chondrocytes)			+
69993	**Chn2**	-1.58	5.14E-03	chimerin (chimaerin) 2		+	+
16859	**Lgals9**	-1.61	2.17E-02	lectin, galactose binding, soluble 9			+
64075	**Smoc1**	-1.62	2.81E-02	SPARC related modular calcium binding 1	+	+	
19126	**Prom1**	-1.70	1.35E-02	prominin 1			+
18750	**Prkca**	-1.74	2.33E-02	protein kinase C, alpha			+
20698	**Sphk1**	-1.96	2.72E-03	sphingosine kinase 1	+		+
227485	**Cdh19**	-2.40	8.54E-03	cadherin 19, type 2		+	

Table S2

GO Term	P-value	Sample frequency	Background frequency	Genes
GO:0006955 immune response	8.15e-07	18/163 (11.0%)	566/33893 (1.7%)	C5ar1 Ccl11 Dhx58 Ccl8 Serping1 Cxcl14 C1s Ifih1 Oasl2 Oas1a Cd8a Col3a1 Cd1d2 Enpp2 Irf7 H2-K1 Cxcl12 Irgm1
GO:0022610 biological adhesion	8.69e-07	20/163 (12.3%)	721/33893 (2.1%)	Tnxb Cdh19 Nid1 Col6a2 Lyve1 Pde3b Mfap4 Fermt1 Antxr1 Smoc1 Gsn Lgals9 F5 Col3a1 Cdh11 Svep1 Mmp2 Lama2 Spon1 Col6a1
GO:0007155 cell adhesion	8.69e-07	20/163 (12.3%)	721/33893 (2.1%)	Tnxb Cdh19 Nid1 Col6a2 Lyve1 Pde3b Mfap4 Fermt1 Antxr1 Smoc1 Gsn Lgals9 F5 Col3a1 Cdh11 Svep1 Mmp2 Lama2 Spon1 Col6a1
GO:0002376 immune system process	1.69e-04	21/163 (12.9%)	1086/33893 (3.2%)	C5ar1 Ccl11 Dhx58 Ccl8 Serping1 Cxcl14 C1s Ifih1 Oasl2 Oas1a Cd8a Col3a1 Cd1d2 Enpp2 Irf7 Bst2 Ifi204 H2-K1 Cxcl12 Irgm1 Prkca
GO:0032963 collagen metabolic process	2.59e-04	6/163 (3.7%)	47/33893 (0.1%)	Tnxb Mmp3 Adamts2 Col3a1 Mmp19 Mmp2
GO:0009611 response to wounding	2.59e-04	14/163 (8.6%)	483/33893 (1.4%)	Ccl11 Ccl8 Serping1 Igf1 Slc1a3 Serpinf1 Anxa5 Papss2 F5 Col3a1 Dysf Mmp2 Sphk1 Prkca
GO:0044259 multicellular organismal macromolecule metabolic process	2.94e-04	6/163 (3.7%)	48/33893 (0.1%)	Tnxb Mmp3 Adamts2 Col3a1 Mmp19 Mmp2
GO:0030198 extracellular matrix organization	3.90e-04	8/163 (4.9%)	123/33893 (0.4%)	Tnxb Nid1 Fbln1 Smoc1 Adamts2 Col3a1 Pdgfra Eln
GO:0006952 defense response	4.07e-04	16/163 (9.8%)	667/33893 (2.0%)	C5ar1 Ccl11 Dhx58 Ccl8 Serping1 C1s Serpinf1 Ifih1 Cd1d2 Irf7 Sphk1 Bst2 H2-K1 Irgm1 Prkca Lsp1
GO:0040011 locomotion	4.09e-04	17/163 (10.4%)	756/33893 (2.2%)	Ang C5ar1 Ccl11 Ccl8 Igf1 Tns1 Slit3 Fermt1 Pdgfra Enpp2 Mmp2 Lama2 Sphk1 Cxcl12 Prkca Lsp1 Enpep
GO:0090066 regulation of anatomical structure size	6.72e-04	13/163 (8.0%)	444/33893 (1.3%)	Ccl11 Igf1 Acta2 Htra1 Slit3 Emp3 Gsn Mmp2 Trp63 Sphk1 Eln Cxcl12 Cda
GO:0044236 multicellular organismal metabolic process	9.33e-04	6/163 (3.7%)	58/33893 (0.2%)	Tnxb Mmp3 Adamts2 Col3a1 Mmp19 Mmp2
GO:0001944 vasculature development	1.05e-03	12/163 (7.4%)	386/33893 (1.1%)	Ang C5ar1 Ccl11 Igf1 Serpinf1 Osr1 Col3a1 Mmp19 Mmp2 Sphk1 Cxcl12 Enpep
GO:0016477 cell migration	1.16e-03	14/163 (8.6%)	547/33893 (1.6%)	Ang C5ar1 Ccl11 Igf1 Tns1 Fermt1 Pdgfra Enpp2 Mmp2 Lama2 Sphk1 Cxcl12 Prkca Enpep
GO:0043062 extracellular structure organization	1.55e-03	9/163 (5.5%)	201/33893 (0.6%)	Tnxb Nid1 Fbln1 Smoc1 Adamts2 Col3a1 Pdgfra Eln Prkca
GO:0048646 anatomical structure formation involved in morphogenesis	2.73e-03	14/163 (8.6%)	588/33893 (1.7%)	Ang C5ar1 Ccl11 Serpinf1 Prom1 Osr1 Tmod1 Mmp19 Mmp2 Trp63 Sphk1 Htt Cxcl12 Enpep
GO:0006508 proteolysis	2.81e-03	16/163 (9.8%)	772/33893 (2.3%)	Pcolce Hp Serping1 C1s Mmp3 Htra1 Dpp10 Adamts5 Cpa3 Dpep1 Adamts18 Adamts2 Ece1 Mmp19 Mmp2 Enpep
GO:0048870 cell motility	3.14e-03	14/163 (8.6%)	595/33893 (1.8%)	Ang C5ar1 Ccl11 Igf1 Tns1 Fermt1 Pdgfra Enpp2 Mmp2 Lama2 Sphk1 Cxcl12 Prkca Enpep
GO:0051674 localization of cell	3.14e-03	14/163 (8.6%)	595/33893 (1.8%)	Ang C5ar1 Ccl11 Igf1 Tns1 Fermt1 Pdgfra Enpp2 Mmp2 Lama2 Sphk1 Cxcl12 Prkca Enpep
GO:0001501 skeletal system development	3.48e-03	10/163 (6.1%)	286/33893 (0.8%)	Igf1 Efemp1 Hoxc8 Cst10 Papss2 Pdgfra Mmp2 Maf Trp63 Prkca
GO:0032535 regulation of cellular component size	3.51e-03	11/163 (6.7%)	357/33893 (1.1%)	Ccl11 Igf1 Htra1 Slit3 Emp3 Gsn Trp63 Sphk1 Eln Cxcl12 Cda

GO:0030574 collagen catabolic process	3.76e-03	4/163 (2.5%)	18/33893 (0.1%)	Mmp3 Adamts2 Mmp19 Mmp2
GO:0010646 regulation of cell communication	3.99e-03	18/163 (11.0%)	994/33893 (2.9%)	Igf1 Slc1a3 Htra1 Slit3 Pde3b Lgals9 Cd8a Ece1 Lama2 Trp63 Sphk1 Bst2 Htt Apcdd1 Cxcl12 Prkca Lrp1 Cilp
GO:0001568 blood vessel development	4.02e-03	11/163 (6.7%)	362/33893 (1.1%)	Ang C5ar1 Ccl11 Serpinf1 Osr1 Col3a1 Mmp19 Mmp2 Sphk1 Cxcl12 Enpep
GO:0008219 cell death	5.88e-03	20/163 (12.3%)	1239/33893 (3.7%)	C5ar1 Hp Igf1 Xrcc2 Anxa5 Osr1 Ifih1 Gsn Bard1 Ece1 Mmp2 Trp63 Sphk1 Ifi204 Htt E2f1 Dram1 Dapl1 Prkca Lsp1
GO:0048468 cell development	6.05e-03	18/163 (11.0%)	1024/33893 (3.0%)	Igf1 Slc1a3 Serpinf1 Slit3 Xrcc2 Cst10 Osr1 Antxr1 Tmod1 Mmp2 Maf Abcb1b Trp63 Sphk1 Htt Cxcl12 Trip13 Prkca
GO:0006928 cellular component movement	6.21e-03	14/163 (8.6%)	631/33893 (1.9%)	Ang C5ar1 Ccl11 Igf1 Tns1 Fermt1 Pdgfra Enpp2 Mmp2 Lama2 Sphk1 Cxcl12 Prkca Enpep
GO:0016265 death	6.48e-03	20/163 (12.3%)	1247/33893 (3.7%)	C5ar1 Hp Igf1 Xrcc2 Anxa5 Osr1 Ifih1 Gsn Bard1 Ece1 Mmp2 Trp63 Sphk1 Ifi204 Htt E2f1 Dram1 Dapl1 Prkca Lsp1
GO:0044243 multicellular organismal catabolic process	7.27e-03	4/163 (2.5%)	21/33893 (0.1%)	Mmp3 Adamts2 Mmp19 Mmp2
GO:0006790 sulfur compound metabolic process	8.45e-03	7/163 (4.3%)	130/33893 (0.4%)	Igf1 Gstm2 Dcn Gstp2 Papss2 Hs3st3b1 Bgn
GO:0009888 tissue development	9.26e-03	17/163 (10.4%)	950/33893 (2.8%)	Ccl11 Igf1 Efemp1 Cst10 Osr1 Adamts2 Col3a1 Mmp2 Maf Abcb1b Trp63 Nup133 Htt Apcdd1 Eln Cxcl12 Prkca
GO:0001525 angiogenesis	9.38e-03	9/163 (5.5%)	250/33893 (0.7%)	Ang C5ar1 Ccl11 Serpinf1 Mmp19 Mmp2 Sphk1 Cxcl12 Enpep
GO:0006915 apoptosis	9.60e-03	19/163 (11.7%)	1168/33893 (3.4%)	C5ar1 Igf1 Xrcc2 Anxa5 Osr1 Ifih1 Gsn Bard1 Ece1 Mmp2 Trp63 Sphk1 Ifi204 Htt E2f1 Dram1 Dapl1 Prkca Lsp1
GO:0050679 positive regulation of epithelial cell proliferation	9.70e-03	6/163 (3.7%)	86/33893 (0.3%)	Ang Ccl11 Igf1 Osr1 Ccnd2 Cxcl12
GO:0072359 circulatory system development	1.07e-02	13/163 (8.0%)	570/33893 (1.7%)	Ang C5ar1 Ccl11 Igf1 Serpinf1 Osr1 Col3a1 Ece1 Mmp19 Mmp2 Sphk1 Cxcl12 Enpep
GO:0072358 cardiovascular system development	1.07e-02	13/163 (8.0%)	570/33893 (1.7%)	Ang C5ar1 Ccl11 Igf1 Serpinf1 Osr1 Col3a1 Ece1 Mmp19 Mmp2 Sphk1 Cxcl12 Enpep
GO:0012501 programmed cell death	1.15e-02	19/163 (11.7%)	1183/33893 (3.5%)	C5ar1 Igf1 Xrcc2 Anxa5 Osr1 Ifih1 Gsn Bard1 Ece1 Mmp2 Trp63 Sphk1 Ifi204 Htt E2f1 Dram1 Dapl1 Prkca Lsp1
GO:0023051 regulation of signaling	1.88e-02	20/163 (12.3%)	1340/33893 (4.0%)	Ccl11 Tnxb Igf1 Slc1a3 Htra1 Slit3 Pde3b Lgals9 Cd8a Ece1 Lama2 Trp63 Sphk1 Bst2 Htt Apcdd1 Cxcl12 Prkca Lrp1 Cilp
GO:0010941 regulation of cell death	2.26e-02	17/163 (10.4%)	1017/33893 (3.0%)	C5ar1 Hp Igf1 Xrcc2 Anxa5 Osr1 Ifih1 Bard1 Cd1d2 Mmp2 Trp63 Sphk1 Ifi204 Htt H2-K1 Prkca Lrp1
GO:0050678 regulation of epithelial cell proliferation	2.69e-02	7/163 (4.3%)	155/33893 (0.5%)	Ang Ccl11 Igf1 Serpinf1 Osr1 Ccnd2 Cxcl12
GO:0040012 regulation of locomotion	3.94e-02	9/163 (5.5%)	299/33893 (0.9%)	C5ar1 Ccl11 Igf1 Pdgfra Enpp2 Lama2 Sphk1 Cxcl12 Prkca
GO:0048585 negative regulation of response to stimulus	4.28e-02	11/163 (6.7%)	465/33893 (1.4%)	Hp Serping1 Igf1 Htra1 Serpinf1 Slit3 Col3a1 Apcdd1 Prkca Lrp1 Cilp
GO:0008283 cell proliferation	4.71e-02	17/163 (10.4%)	1077/33893 (3.2%)	Ang Ccl11 Igf1 Sparc Serpinf1 Slit3 Ifitm3 Fermt1 Osr1 Ccnd2 Pdgfra Trp63 Sphk1 Cxcl12 Prkca Lrp1 Enpep
GO:0040017 positive regulation of locomotion	4.71e-02	7/163 (4.3%)	169/33893 (0.5%)	C5ar1 Ccl11 Igf1 Pdgfra Sphk1 Cxcl12 Prkca
GO:0048584 positive regulation of response to stimulus	4.84e-02	14/163 (8.6%)	757/33893 (2.2%)	C5ar1 Serping1 Igf1

Table S3

Rank	Gene	reg.	p-value
1	COL11A1	-1.38	1.34E-01
2	THBS2	+1.09	6.14E-01
3	COL10A1	-1.10	7.15E-01
4	COL5A2	+1.20	1.48E-01
5	INHBA	+1.05	7.04E-01
6	LRRC15	+1.45	1.79E-01
7	COL5A1	+1.37	7.60E-02
8	VCAN	+1.59	1.53E-01
9	FAP	+1.07	6.31E-01
10	COL1A1	+1.42	5.99E-02
11	**MMP11**	**+1.36**	**3.92E-02**
12	POSTN	+1.13	5.04E-01
13	COL1A2	+1.48	5.36E-02
14	**ADAM12**	**+1.25**	**2.45E-02**
15	**COL3A1**	**+1.75**	**2.65E-02**
17	FN1	+1.61	1.16E-01
18	AEBP1	+1.18	2.84E-01
19	SULF1	+1.15	2.38E-01
20	FBN1	+1.58	6.88E-02
21	ASPN	+1.06	7.92E-01
22	**SPARC**	**+1.60**	**6.11E-03**
23	CTSK	+1.30	5.71E-02
24	TNFAIP6	+1.33	2.59E-01
25	HNT	-1.03	3.51E-01
26	EPYC	-1.01	8.68E-01
27	**MMP2**	**+1.72**	**9.00E-03**
28	PLAU	-1.01	8.68E-01
29	GREM1	+1.05	6.94E-01
30	**BGN**	**+1.45**	**9.06E-03**
31	OLFML2B	+1.23	**1.53E-01**
32	**LUM**	**+1.98**	**3.93E-02**

Rank	Gene	reg.	p-value
33	LOXL2	+1.50	1.08E-01
34	COL6A3	+1.32	4.06E-01
35	IgsF10	+1.50	1.23E-01
36	**MFAP5**	**+1.55**	**3.08E-02**
37	NUAK1	+1.20	1.44E-01
38	RAB31	+1.19	6.98E-02
39	TIMP3	+1.12	6.10E-01
40	CRISPLD2	-1.14	5.31E-01
41	**ITGBL1**	**+1.49**	**1.23E-02**
42	**CDH11**	**+1.24**	**8.70E-03**
43	TMEM158	+1.01	9.37E-01
44	SPOCK1	-1.02	7.31E-01
45	SFRP4	+1.44	2.21E-01
46	**SERPINF1**	**+1.81**	**3.78E-05**
47	**DCN**	**+1.96**	**4.75E-03**
49	COPZ2	-1.09	5.68E-01
50	NOX4	+1.08	7.01E-01
51	EDNRA	+1.16	6.48E-01
52	ACTA2	+1.66	7.66E-02
53	**PDGFRB**	**+1.31**	**2.84E-03**
54	**RCN3**	**+1.95**	**7.10E-03**
55	SNAI2	+1.43	1.02E-01
56	C1QTNF3	-1.29	2.10E-01
57	COMP	-1.61	1.53E-01
58	**LGALS1**	**+1.38**	**8.03E-03**
59	THY1	+1.35	1.53E-01
60	**PCOLCE**	**+1.39**	**3.84E-02**
61	**COL6A2**	**+1.62**	**1.23E-02**
62	GTL8D2	+1.05	5.99E-01
63	**NID2**	**+1.27**	**9.82E-03**
64	**PRRX1**	**+1.43**	**3.01E-02**

Figure S1

A

Patient	CAG number	Age of diagnosis: HD	Age of diagnosis: Cancer	Grade (EE)	Phenotype	Axillary lymph node status
1	45	47	39	II	ER+ PR+ HER2-	N0
2	44	49	54	III	ER- PR- HER2-	N1
3	43	45	59	III	ER+ PR+ HER2+	N1
4	42	60	50	n.a.	n.a.	n.a.
5	41	56	62	II	n.a.	N1
6	41	45	54	III	ER- PR- HER2+	N1
7	40	64	74	n.a.	n.a.	n.a.
8	45	50	53	III	ER- PR+ HER2-	n.a.
9	45	43	49	III	ER- PR- HER2+	N1
10	42	AS	56	II	n.a.	N0
11	42	65	68	n.a.	n.a.	n.a.
12	42	60	40	n.a.	n.a.	n.a.

B

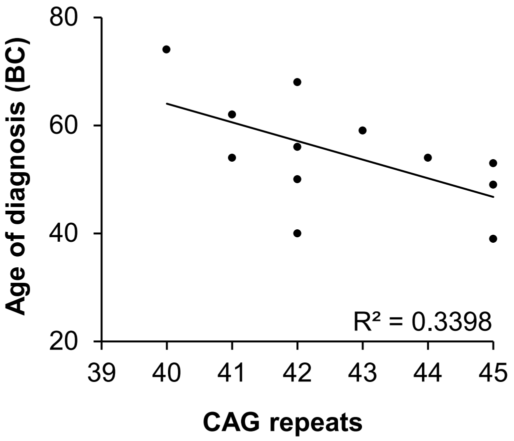

C

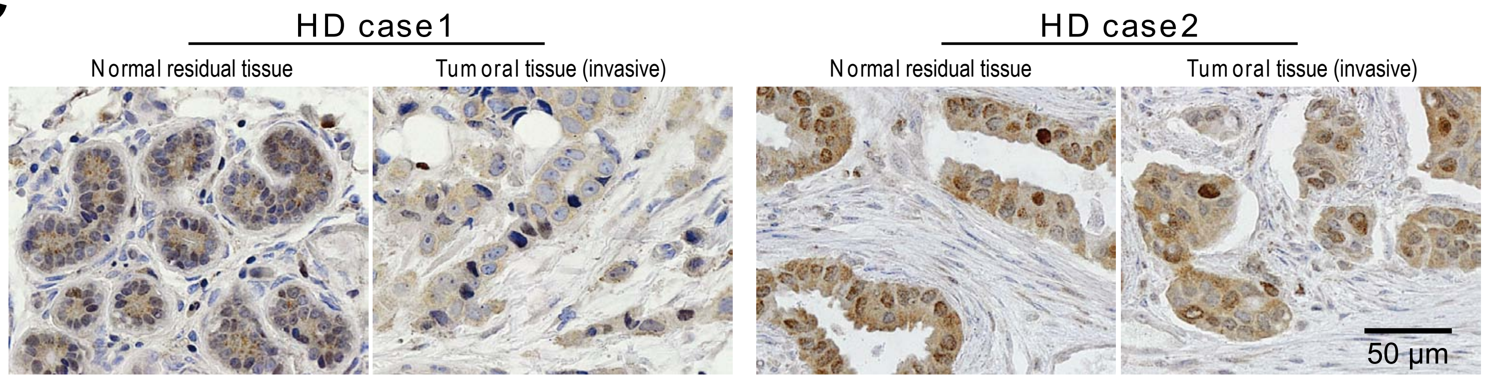

D

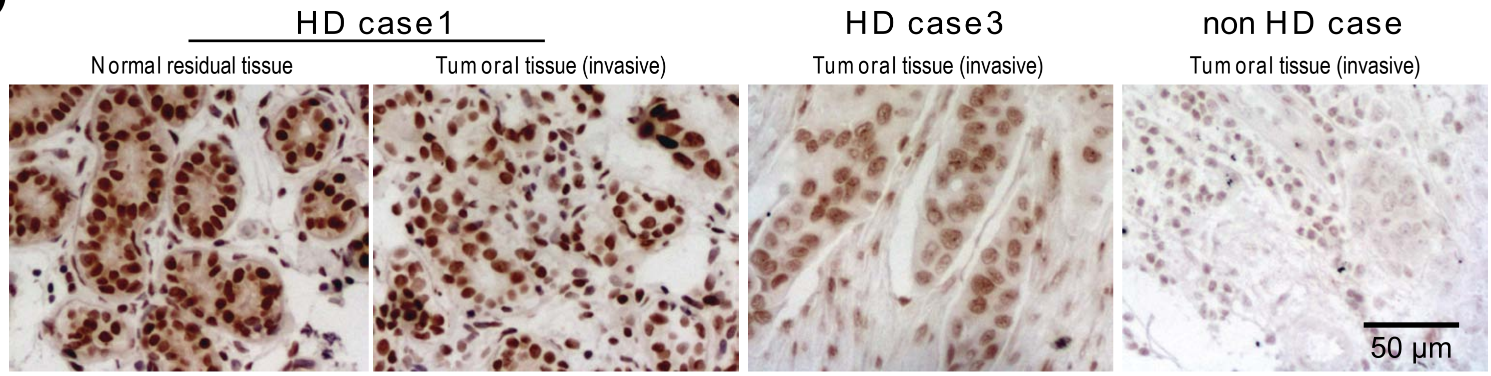

Figure S2

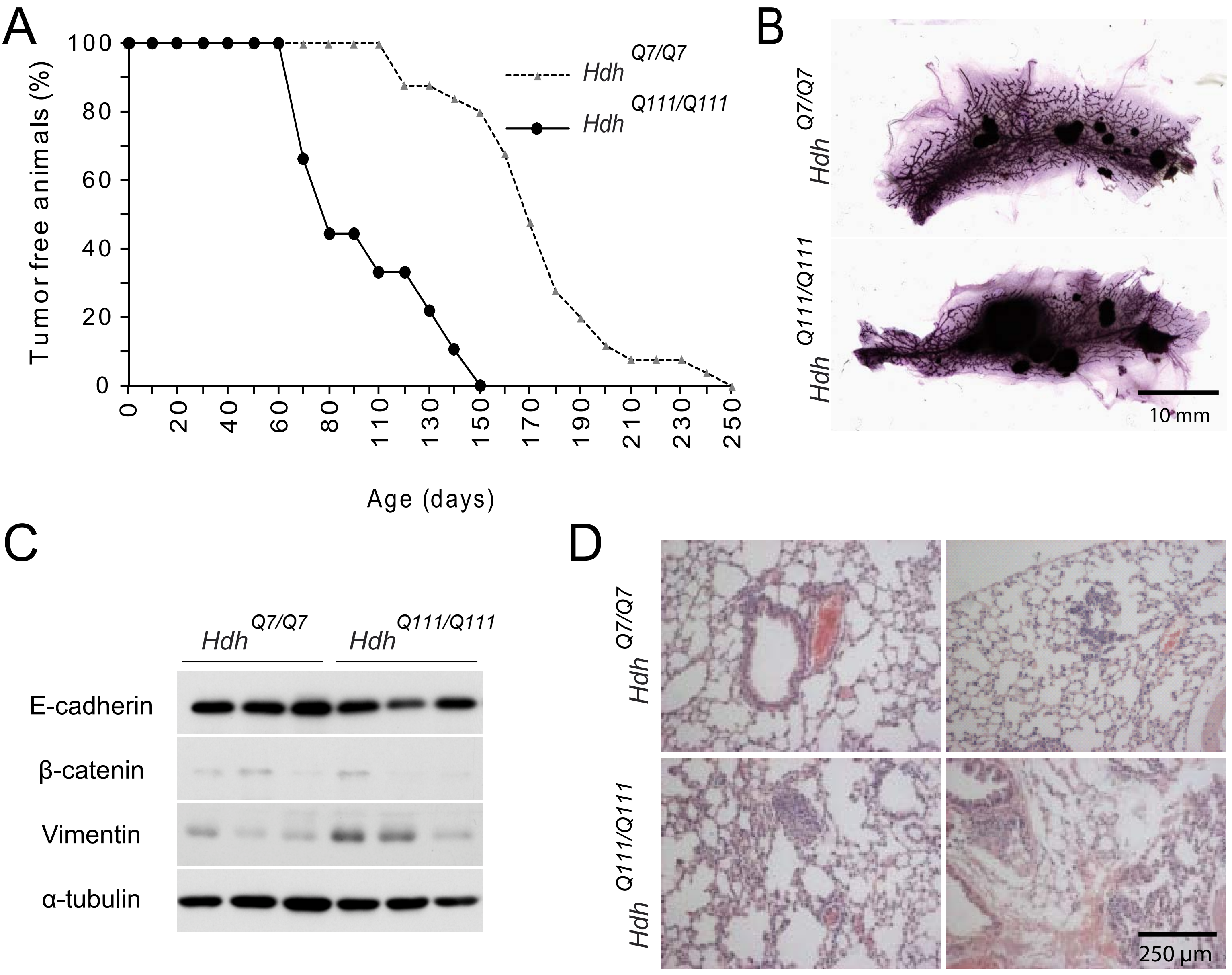
A
Tumor free animals (%)
100
80
60
40
20
0
0
20
40
60
80
110
130
150
170
190
210
230
250
Age (days)
Hdh Q7/Q7
Hdh Q111/Q111
B
Hdh Q7/Q7
Hdh Q111/Q111
10 mm
C
Hdh Q7/Q7
Hdh Q111/Q111
E-cadherin
β-catenin
Vimentin
α-tubulin
D
Hdh Q7/Q7
Hdh Q111/Q111
250 μm

Figure S3

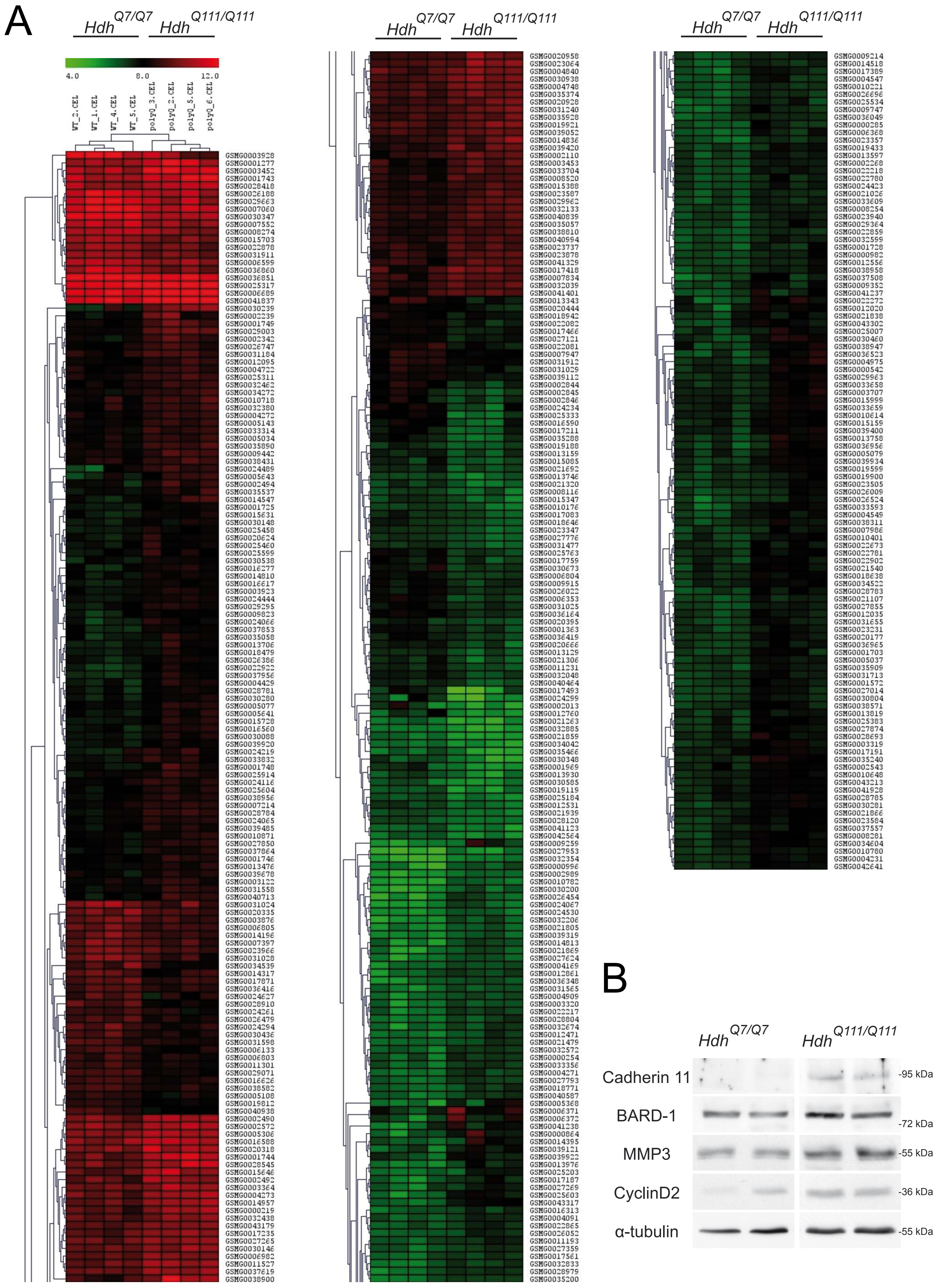
A
Hdh Q7/Q7
Hdh Q111/Q111
4.0
8.0
12.0
WT_2.CEL
WT_1.CEL
WT_4.CEL
WT_5.CEL
polyQ_3.CEL
polyQ_2.CEL
polyQ_5.CEL
polyQ_6.CEL
B
Cadherin 11
BARD-1
MMP3
CyclinD2
α-tubulin
-95 kDa
-72 kDa
-55 kDa
-36 kDa
-55 kDa

Figure S4

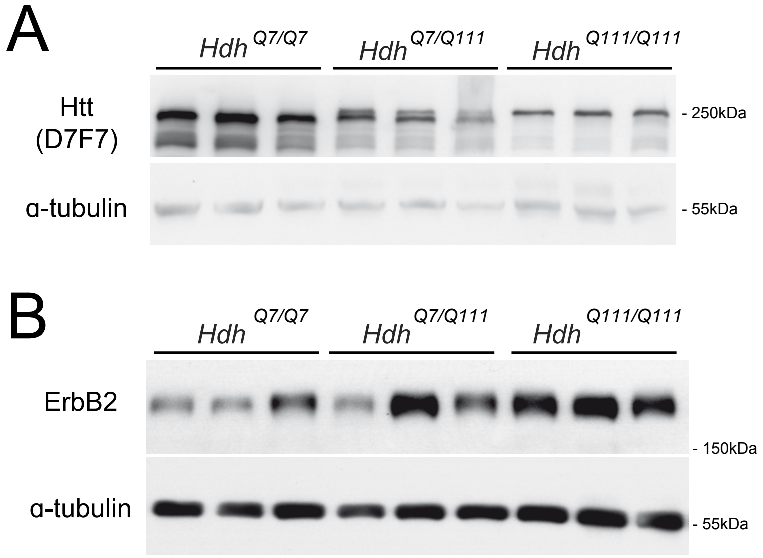
A
Hdh Q7/Q7
Hdh Q7/Q111
Hdh Q111/Q111
Htt (D7F7)
α-tubulin
- 250kDa
- 55kDa
B
Hdh Q7/Q7
Hdh Q7/Q111
Hdh Q111/Q111
ErbB2
α-tubulin
- 150kDa
- 55kDa
